# Unveiling the Uncharted Potential of 2D Materials in Li/Na–S Batteries: A Paradigm Shift From Graphene

**DOI:** 10.1002/advs.77187

**Published:** 2026-08-18

**Authors:** Naveen Kumar T. R, Xuebing Zhu, Yao‐Jie Lei, Lei Zhang, Hua Kun Liu, Weihong Lai, Guoxiu Wang, Yun‐Xiao Wang

**Affiliations:** ^1^ Institute of Energy Materials Science (IEMS) University of Shanghai for Science and Technology Shanghai People's Republic of China; ^2^ Centre For Clean Energy Technology, School of Mathematical and Physical Sciences, Faculty of Science University of Technology Sydney Sydney New South Wales Australia; ^3^ Centre For Catalysis and Clean Energy, Gold Coast Campus Griffith University Southport Queensland Australia; ^4^ Australian Institute for Innovative Materials (AIIM), Innovation Campus University of Wollongong (UOW) North Wollongong New South Wales Australia; ^5^ Laboratory of Advanced Materials, Shanghai Key Laboratory of Molecular Catalysis and Innovative Materials, School of Chemistry and Materials Fudan University Shanghai People's Republic of China

**Keywords:** 2D materials, alkali metal‐sulfur batteries, Li/Na–S, operando techniques, machine learning

## Abstract

Rechargeable metal‐sulfur batteries (MSBs) are promising next‑generation energy storage systems because of their high theoretical energy density and the abundance of sulfur. However, their commercialization is impeded by persistent challenges, including shuttle effect, dendrite formation, volume expansion, and sluggish redox kinetics. To mitigate these issues, two‑dimensional (2D) materials, which possess tunable physicochemical, electronic, and mechanical properties, have emerged as promising candidates. Despite significant progress, many conventional 2D host and interfacial materials still exhibit weak polysulfide adsorption and inadequate interfacial compatibility, limiting the efficiency of MSBs. This review provides a comprehensive overview of emerging 2D materials beyond graphene, including graphdiyne, phosphorene, borophene, siloxene, silicene, MBene, antimonene, and germanene. The functional roles of these materials in battery components sulfur hosts, anode protection layers, separators, and electrolytes are systematically analyzed in terms of their ability to suppress polysulfide migration, suppress dendrite growth, and stabilize electrode interfaces. Special emphasis is placed on the integration of machine learning (ML) and advanced operando characterization techniques to accelerate the discovery of 2D materials for MSBs. Finally, the review assesses practical barriers, such as scalable synthesis of 2D materials and industrial adoption of ML‑driven design workflows, and outlines next‑generation 2D material strategies to bridge fundamental research and real‑world deployment.

## Graphical abstract

This review explores emerging 2D materials beyond graphene, including graphdiyne, phosphorene, borophene, siloxene, MBene, antimonene, and germanene for Li/Na–S batteries. It analyzes their roles as sulfur hosts, metallic anode protectors, separators, and electrolyte fillers, emphasizing polysulfide suppression, dendrite inhibition, and interfacial stability. Machine learning and in situ characterization strategies are also highlighted.

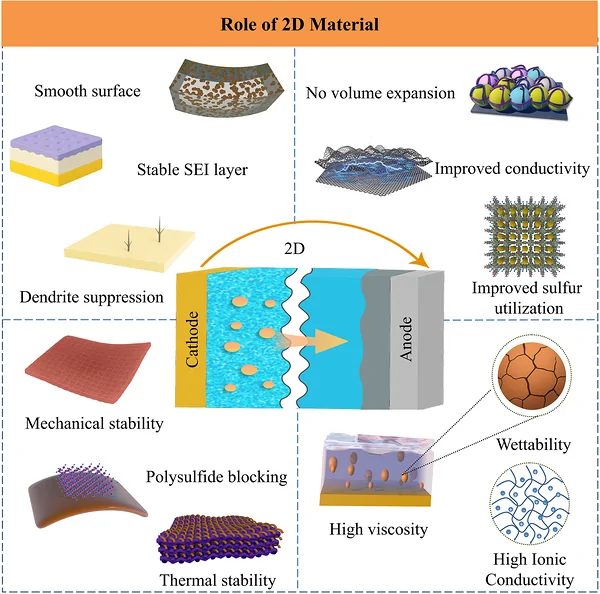

## 1 Introduction

Lithium‐ion batteries (LIBs) have emerged as vital components in the global transition from fossil‐fuel‐based energy systems to emission‐free electrification. LIBs dominate the market because of their high gravimetric and volumetric energy densities, fast‐charging capability, and long cycle life [1, 2, 3, 4, 5, 6, 7]. However, conventional LIB chemistries, such as LiFePO_4_ and NCM, are approaching their theoretical energy‐density limits of 300 Wh kg^−1^ because of the intrinsic limitations of the Li^+^ insertion/extraction process. In contrast, the energy requirements of aviation, automobiles, and power grids require batteries with much higher energy densities, approaching 500 Wh kg^−1^ [8, 9, 10]. This limitation has shifted research interest from intercalation‐type LIBs to high‐energy alkali‐metal batteries (AMBs). AMB systems that use lithium (Li), sodium (Na), or potassium (K) metal anodes have attracted considerable attention because of their multi‑electron redox chemistry. Their low electrochemical potentials (−3.04 V for Li, −2.71 V for Na, and −2.93 V for K versus the standard hydrogen electrode) and high theoretical specific capacities (3.86, 1.16, and 0.68 Ah g^−1^ for Li, Na, and K, respectively) further enhance their appeal. Moreover, alkali‑metal anodes can be paired with a variety of high‑energy‑density cathodes, including sulfur (S), oxygen (O_2_), and carbon dioxide (CO_2_), making them promising candidates for next‑generation rechargeable batteries. Despite decades of research, AMBs continue to face persistent challenges [10, 11, 12, 13, 14, 15, 16].

**FIGURE 1:**
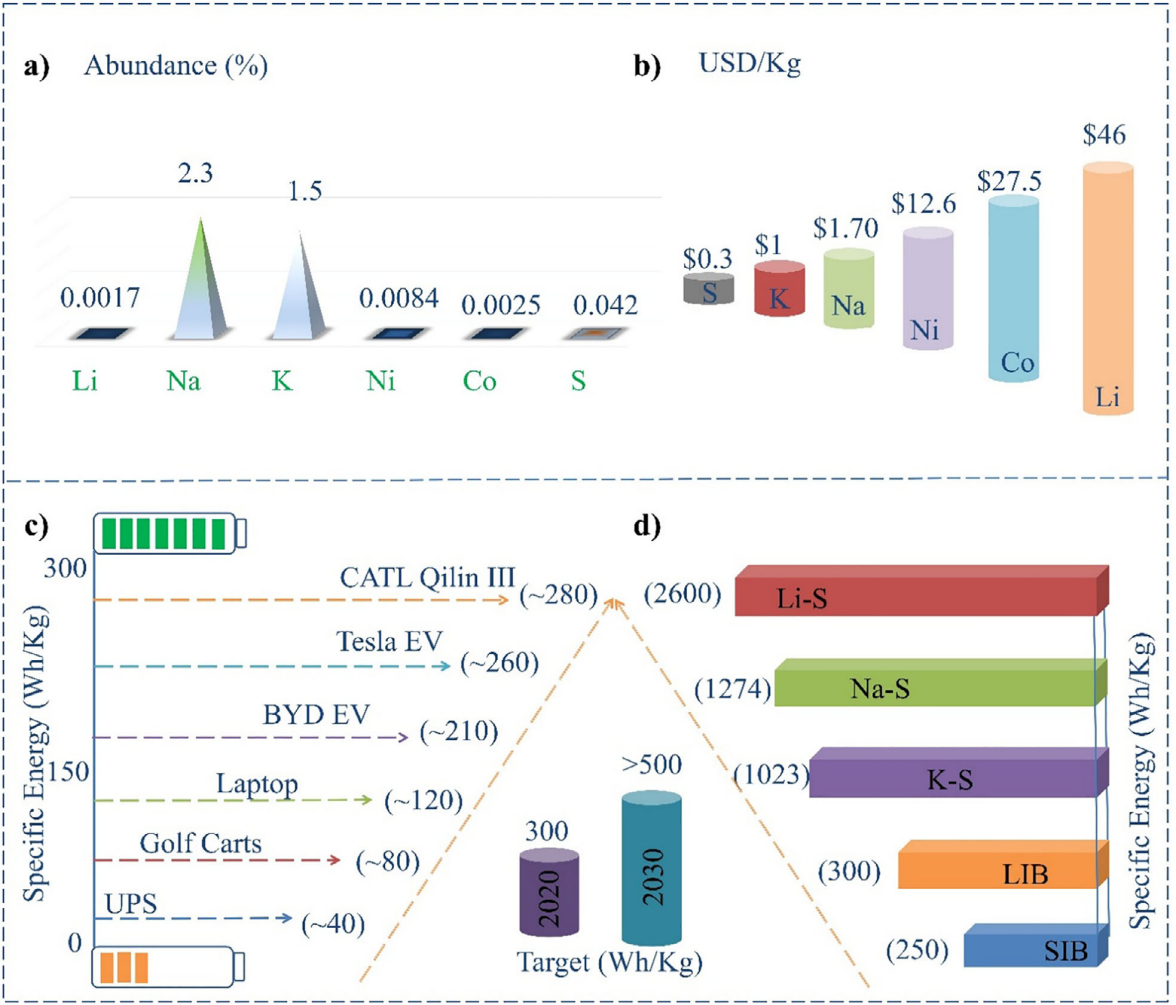
(a) Abundance (PPM), (b) price comparison of S, Na, Ni, Li, Co and K elements in USD/kg, (c) current specific energy (Wh/Kg) for different application and required specific energy in 2030 and (d) theoretical ceiling energy densities of various batteries (SIB, LIB, K–S, Na–S, and Li–S), highlighting importance of MSB.

Although metal‐oxygen (M–O_2_) batteries offer high theoretical energy densities 3456 Wh kg^−1^ for Li–O_2_, 1105 Wh kg^−1^ for Na‐O_2_, 2205 Wh kg^−1^ for Mg‐O_2,_ and 820 Wh kg^−1^ for Zn‐O_2_ [17, 18] their practical feasibility is severely limited by poor reversibility, parasitic side reactions, and unstable discharge products. In Li–O_2_ systems, the formation of superoxide ions (O_2_^−^) leads to the accumulation of irreversible Li_2_O_2_/Li_2_O species and promotes dendritic growth, thereby reducing coulombic efficiency (CE). These systems are highly susceptible to contamination from water and ambient CO_2_. Water acts as a soluble catalyst, lowering charge polarization and increasing discharge capacity. However, it ultimately degrades rate capability and cycling stability. Meanwhile, CO_2_ dissolves in organic electrolytes and reacts with O_2_^−^ to form insulating metal carbonates, which increase overpotential, hinder reversibility, and reduce voltage efficiency and cycle life [19, 20, 21]. Similarly, metal‐carbon dioxide (M‐CO_2_) batteries suffer from high polarization, sluggish reaction kinetics, and severe parasitic reactions, leading to cathode corrosion and electrolyte decomposition. Moreover, their relatively high operating voltages (≈4 V) increase the risk of thermal runaway [22, 23, 24]. In contrast, metal–sulfur batteries (MSBs), such as Lithium Sulfur (Li–S) and Sodium Sulfur (Na–S) systems, represent a promising alternative due to their multi‐electron redox reactions, low toxicity, and environmental sustainability. Compared with M‐O_2_ and M‐CO_2_ systems, MSBs exhibit improved electrolyte compatibility and reversibility. They also provide high theoretical energy densities of 2600 Wh kg^−1^ (Li–S), 1274 Wh kg^−1^ (Na–S), and 1023 Wh kg^−1^ (Potassium Sulfur (K–S). Figure 1a–d highlights the significance of MSBs for next‐generation energy storage. Specifically, Figure 1a,b illustrates the elemental abundance and cost advantages of active materials, while Figure 1c presents future application targets and current specific energy requirements for backup/UPS systems (∼40 Wh kg^−1^) to electric vehicle battery packs (∼220–280 Wh kg^−1^). Figure 1d compares the theoretical energy density limits of various battery chemistries (Sodium Ion Batteries (SIB), Lithium Ion Batteries (LIB), K–S, Na–S, and Li–S), further emphasizing the potential of MSBs [24, 25, 26, 27, 28]_._ However, the commercialization of MSBs remains hindered by several interconnected challenges, including the insulating nature of sulfur and its discharge products, significant volume expansion of the sulfur cathode, and the formation of unstable solid‐electrolyte interphase (SEI) layers on alkali metal anodes. Additionally, the polysulfide shuttle effect, dendrite growth, and low ionic conductivity lead to rapid capacity fading, low coulombic efficiency (CE), and self‐discharge [24, 29, 30, 31].To date, various bulk materials have been employed in different components of MSBs. However, they fail to overcome the multifaceted challenges. These include the insulating nature of sulfur, shuttle effect, volume expansion of the sulfur cathode, formation of unstable solid‐electrolyte interphase (SEI) layers on alkali metal anodes, dendrite growth, and low ionic conductivity [24, 29, 30, 31]. This inadequacy stems from the isotropic properties of bulk materials. It is worth noting that these bulk materials lack directional pathways for electron and ion transport, that result in increased internal resistance and hinders uniform ion migration. Further, its low surface area reduces its active sites, which leads to sluggish redox reactions [32]. Bulk material also fails to accommodate volume expansion due to its rigid structure, which leads to particle pulverization, loss of electrical contact, and rapid capacity fade over prolonged cycling. Moreover, its weak and non‐selective interaction with intermediate species fails to suppress the shuttle effect, which results in continuous active material loss and low Coulombic efficiency (CE). On the anode, a non‐conformal interface forms between the bulk host and metal surface due to its poor wettability, surface roughness mismatch, and non‐uniform metal deposition, which promote localized nucleation and dendrite formation [32, 33, 34].

Two‐dimensional (2D) materials circumvent this limitation by offering a structurally anisotropic platform with orthogonal functionalities. Their in‐plane covalent or metallic bonds offer continuous electron transport that overcomes sulfur's insulation [35]. Further it reduced dimensionality enables redox‐active sites that remain electrochemically accessible even at high mass loadings. Furthermore, its interlayer spacing can be expanded or contracted to match the solvated diameter of Li^+^ or Na^+^. This accelerates the charge‐transfer kinetics beyond the diffusional limits of porous carbons. Lastly, upon Li/Na deposition, the layers dissipate local stress concentrations and limit dendrite growth, while the in‐plane stiffness resists electrode cracking. For example, graphene exhibits an exceptional surface‐area‐to‐volume ratio, increasing the number of electrochemically active sites. Its porous and conductive network enables uniform sulfur dispersion and creates tortuous diffusion pathways that confine M_2_S_x_ species via van der Waals interactions. Furthermore, functionalization or heteroatom doping introduces polar and coordination sites that enhance adsorption energies, enabling strong chemisorption of polysulfides and promoting their catalytic conversion to M_2_S, while its high electrical conductivity ensures efficient charge transport within MSBs [36, 37, 38, 39]. In contrast, g‐C_3_N_4_ contains abundant pyridinic and graphitic nitrogen sites that form M–N (M═Li, Na) coordination interactions, enabling binding of polysulfide species and significantly improving cycling stability when used as cathode hosts or separator coatings. Additionally, g‐C_3_N_4_ exhibits tunable mechanical and thermal properties that mitigate resistive heat generation during cycling, thereby enhancing CE [40, 41]. Layered MXenes and transition metal dichalcogenides (TMDCs) can accommodate volume changes during repeated charge/discharge cycles, preserving electrode structural integrity [36, 37, 38]. Recent research has expanded beyond conventional materials to include emerging 2D systems, such as graphdiyne (GDY), phosphorene, borophene, siloxene, silicene, MBenes, antimonene, and germanene. GDY combines high electrical conductivity with mechanical flexibility, making it suitable for both electrode frameworks and separator reinforcement [34]. Antimonene demonstrates superior electrochemical stability compared to traditional catalysts, which often suffer from capacity degradation due to side reactions. Borophene, with its negative Poisson's ratio and high anisotropic Young's modulus (280 GPa), resists structural degradation and maintains electrode integrity during long‐term cycling [42, 43]. Despite these advances, current research remains heavily focused on graphene‐based materials.

This review comprehensively examines the potential applications of MSBs, emphasizing their intrinsic challenges, including sulfur cathodes, metallic anodes, separators, and electrolytes. Significant focus has been placed on emerging 2D materials (GDY, phosphorene, borophene, siloxene, silicene, MBene, antimonene and germanene) in addition to traditional materials (graphene, g‐C_3_N_4_, MXene, 2D‐MOF, 2D‐COF and 2D‐TMDCs). Furthermore, through a judicious combination of theoretical and experimental analyses, we highlight the key attributes of these 2D materials as robust cathodes, particularly in addressing the shuttle effect and enhancing kinetic reactions. Second, we evaluated the effectiveness of 2D materials in metallic anodes by examining their roles in artificial SEI layers and mechanical buffering via composite and alloy‐based hosts. Third, we discuss separator modifications, including polysulfide blocking and enhanced thermal and mechanical stabilities. We also emphasize the significance of incorporating 2D fillers into electrolyte formulations, which enables the modulation of ion transport improve the mechanical properties. In addition, this review highlights the importance of advanced in situ techniques and machine‐learning (ML) methods for accelerating the discovery and 2D material design. Finally, to stimulate further development, we present future perspectives on emerging 2D materials in multivalent battery chemistry and highlight the scalability, cost, environmental impact, industrial adoption, and ML integration. We believe that this review will provide a foundation for developing more efficient and effective energy storage solutions in the evolving landscape of battery technology.

## 2 Fundamental Challenges in MSBs

**FIGURE 2:**
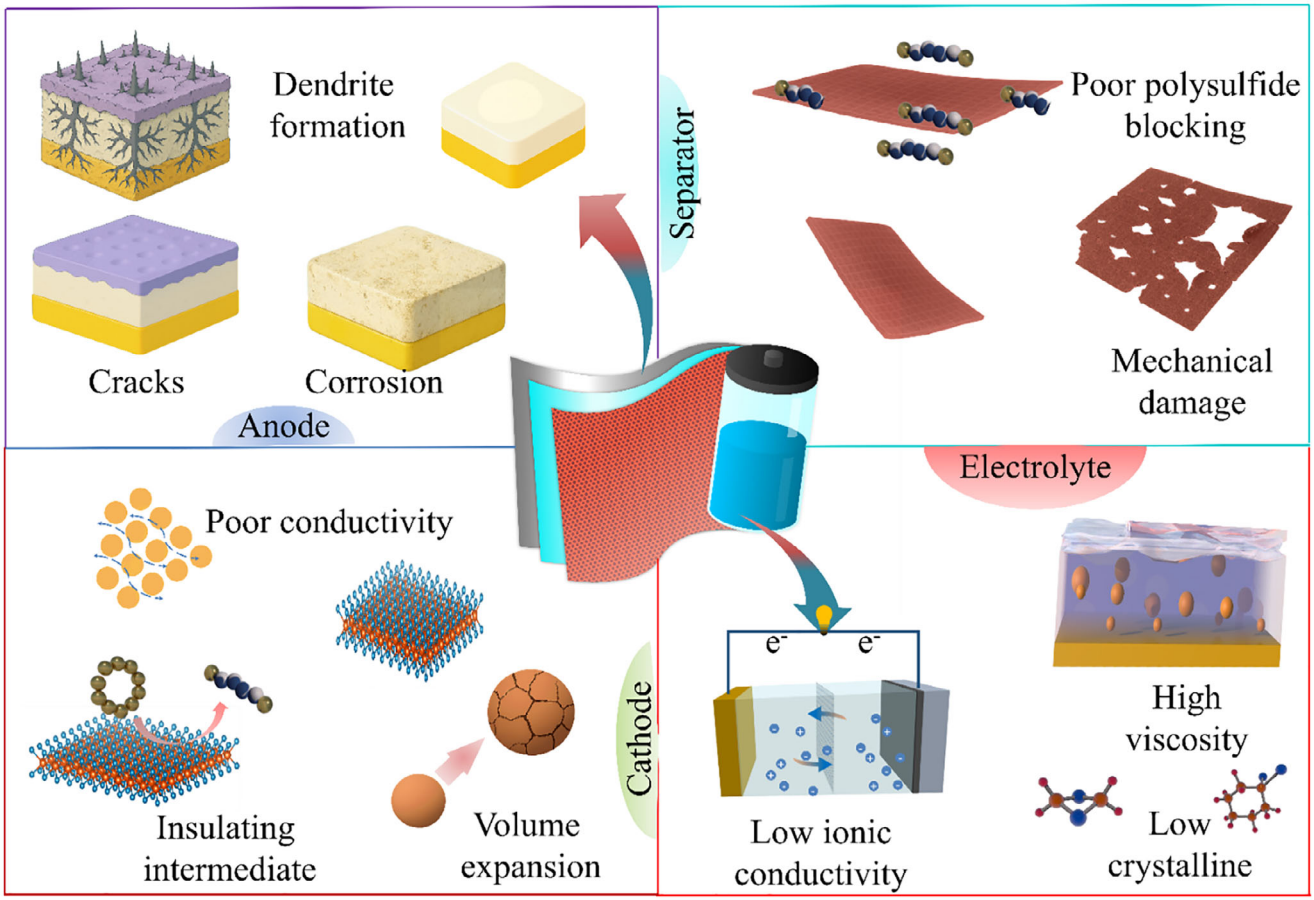
Schematic illustration of MSB challenges. Key issues include cracks, dendritic growth, corrosion in the anode, poor polysulfide blocking, mechanical damage in the separator, low ionic conductivity, high viscosity, low crystallinity of electrolyte, insulating intermediate, and poor conductivity at the sulfur cathode.

Although MSBs hold immense promise for high‐energy storage, their practical feasibility is impeded by several interdependent challenges associated with the sulfur cathodes, metallic anodes, electrolytes, and separators (Figure 2). These limitations collectively result in poor cycling stability, limited reversibility, and limited CE values. Understanding these bottlenecks is crucial for developing rational material and system‐level design strategies, particularly for advanced 2D frameworks.

### 2.1 Sulfur Cathode

The sulfur cathode faces three interconnected challenges that hinder the MSB performance. First, elemental sulfur (∼5 × 10^−30^ S cm^−1^) and its fully discharged products (Li_2_S and Na_2_S) are intrinsically insulating, which severely impedes electron and ion transport. This poor conductivity results in sluggish reaction kinetics, poor electrochemical reversibility, and reduced capacity utilization. Second, soluble long‐chain polysulfide intermediates (Li_2_S_x_, Na_2_S_x_) generated during the discharge cycle dissolve into the electrolyte and migrate toward the metal anode under an electrochemical potential gradient. This parasitic “shuttle effect” induces undesirable redox reactions at the anode, forming insoluble metal sulfides (M_x_S) that passivate the electrode surface, deplete active material, and diminish CE. The continuous dissolution‐migration‐reprecipitation cycle further exacerbates the spatial redistribution of active species, promoting heterogeneous deposition, increased internal resistance, and severe electrochemical polarization. Third, the sulfur cathode undergoes significant volumetric expansion during cycling due to the density disparity between the sulfur and its discharge products. Elemental sulfur (S_8_, 2.07 g cm^−3^) converts to Li_2_S (∼1.66 g cm^−3^) or Na_2_S (∼1.86 g cm^−3^), resulting in volume expansions of ∼80% in Li–S and ∼170% in Na–S batteries. These large mechanical stresses cause particle pulverization, loss of electrical contact with conductive additives, and accelerated capacity fading. Moreover, repeated expansion‐contraction cycles expose fresh sulfur surfaces to the electrolyte, promoting additional polysulfide dissolution and aggravating the shuttle effect [44, 45, 46].

### 2.2 Alkali Metal Anode

Challenges at the anode interface are critical. During repeated metal plating and stripping, the alkali metal anode undergoes substantial volumetric fluctuations, leading to SEI fracturing, continuous electrolyte consumption, and dendritic growth. These mechanical instabilities are exacerbated by the continuous metal deposition process, resulting in the formation of ‘dead’ metal and elevated interfacial resistance. Unlike the cathode, where volume changes arise from phase transformations of sulfur species, the anode volume expansion is primarily governed by non‐uniform current distribution and heterogeneous nucleation during electrodeposition. The fragile SEI undergoes repetitive reconstruction and thickening, which further diminishes CE and accelerates electrolyte depletion. The continuous dendrite growth degrades the cycling stability and poses severe risks, including internal short circuits and thermal runaway, which result in shorter lifespan [44, 45, 46].

### 2.3 Electrolyte Challenges

Electrolytes represent a critical bottleneck in MSBs owing to their limited chemical compatibility with sulfur species, insufficient ionic transport, and inadequate interfacial stability with alkali–metal anodes. In practical Li–S and Na–S systems, the major electrolyte‐related challenges arise from parasitic reactions with dissolved polysulfides, unstable solid‐electrolyte interphase (SEI) formation on reactive metal anodes, and sluggish ion transport under lean‐electrolyte or high‐loading conditions. These issues further aggravate and cannot regulate interfacial uniformity during repeated metal plating/stripping. As a result, electrolytes strongly influence polysulfide shuttling, interfacial degradation, non‐uniform ion flux, continuous electrolyte consumption, and poor CE, ultimately reducing battery lifespan and compromising safety concerns [47, 48, 49, 50].

### 2.4 Separator Challenges

Although electrolyte instability poses significant challenges, separators have their own set of limitations. A separator with poor ion conductivity limits the battery performance. Throughout cycling, separators are subjected to repeated volume fluctuations associated with metal intercalation and deintercalation and dynamic electrode expansion. Maintaining the structural integrity of the separator under harsh conditions is of paramount importance. A thin or brittle separator with insufficient tensile strength or elasticity leads to tearing, pore collapse, or deformation, resulting in a short circuit. Furthermore, exposure of the separator to reactive polysulfide species in aggressive electrolyte environments degrades the separator matrix, resulting in undesirable side reactions.

To overcome these challenges, holistic design strategies that are capable of tuning multiple interfaces are required. 2D materials are compelling candidates for engineering each component of MSBs owing to their unique physicochemical features. The properties of 2D materials, including their large surface area, robust mechanical strength, tunable electronic structure, and rich surface chemistry, mitigate the drawbacks of MSBs, such as anode dendrite formation, cathode polysulfide dissolution, and separator interfacial instability. In the following sections, we explore the diverse roles of 2D materials in overcoming these limitations.

## 3 Engineering Capabilities of 2D Materials

2D materials, with strong in‐plane covalent bonds and weak out‐of‐plane van der Waals (VDW) interactions, are versatile candidates for overcoming MSB limitations [51]. Their structural anisotropy offers several benefits, including an optimized surface‐to‐volume ratio, selective ion storage/transport, high charge transfer, a dendrite‐free anode, high redox reaction, enhanced electrochemical activity, and superior mechanical and thermal stability. 2D materials were considered ideal cathode matrices owing to their high redox reaction, large surface area, and electrical conductivity [52, 53, 54]. For instance, the integration of sulfur into 2D matrices has numerous advantages. First, the excellent conductivity of the 2D material (graphene) overcomes the limitations of sulfur's insulating nature, thereby improving redox kinetics and rate capability [36]. Second, functionalized 2D materials, such as MXene and 2D‐MOFs enriched with functional groups (OH, ─COOH, etc.) facilitate stronger chemical interactions with polysulfide intermediates and accelerate sluggish reaction kinetics [32, 59]. Third, porous 2D frameworks, namely Covalent Organic Frameworks (COFs), offer a high surface area that improves sulfur loading, increasing the energy density of the battery [55]. Fourth, the diverse architectures of 2D materials, including buckling, planar, and puckered structures, increase the interlayer distance. These enable larger ions to be accommodated, buffer the volume expansion, and decrease the energy barrier for ion transport [54, 56, 57, 58, 59]. In the case of the anode, 2D materials with superior mechanical properties, such as Young's modulus, bulk modulus, Shear Modulus, and Tensile strength, prevent electrode cracking and restrict the growth of metallic dendrites in anodic electrodes [54, 60, 61, 62, 63]. By modulating Poisson's ratio, the lateral expansion and axial stiffness of the electrodes are engineered, further enhancing the structural resilience during repeated cycling [64, 65, 66]. As a result, 2D materials increase the energy density and specific capacity of MSBs. Importantly, the crystalline symmetry and anisotropy of 2D materials provide routes for tailoring their physicochemical properties for the different components of MSBs. For instance, phonon propagation is confined within a 2D plane, suppressing cross‐plane heat transfer and reducing the risk of thermal runaway [65]. In contrast, the thermal conductivity of 2D materials is enhanced via strong electron‐phonon coupling, which benefits heat dissipation [67, 68]. Furthermore, 2D materials with insulating characteristics (boron nitride) serve as separator coatings to inhibit polysulfide conversion and minimize the shuttle effect, which degrades the battery capacity [54, 69, 70].

Beyond electrodes and separators, 2D materials also enhance electrolytes by modifying their key physicochemical properties. 2D additives/fillers alter the absorption/diffusion coefficient of the electrolyte, which plays a pivotal role in polysulfide ion absorption and conversion kinetics. For example, additives interact with polysulfides based on diffusion coefficients. These interactions change the ionic species content, ultimately altering conductivity. 2D materials with different interlayer spacings (2D‐MOF), when added as fillers, alter the crystallinity of the electrolyte (PEO matrix), directly affecting the ionic conductivity. The presence of 2D fillers improves the interfacial interactions between the solvents, resulting in fast ion transport [71]. In addition, 2D materials tune the mechanical properties of electrolytes, which influences the dissolution of metallic ions and the electrode‐electrolyte interface [72, 73, 74, 75]. Furthermore, 2D materials, namely 2D‐TMDCs, are also beneficial in inhibiting the shuttle effect in MSBs [76]. It is also important to note that 2D materials with hierarchical porous structures, such as micro (<2 nm), meso (2–50 nm), and macro‐pores (> 50 nm), resulting from a large aspect ratio (lateral to thickness), display numerous benefits, such as interconnected mesopores and macro‐pores, which promote more active sites for ion adsorption and storage. In addition, by altering the porosity of 2D materials, ion transport is improved by restricting electrolyte penetration. Table 1 highlights the limitations of MSBs and shows how properties of 2D materials overcome those challenges. Overall, 2D materials overcome the daunting challenges of MSBs, and researchers have explored numerous 2D materials. Despite these advancements, most reviews emphasized traditional 2D catalysts.

**TABLE 1:** Multifunctional roles of 2D materials in MSBs.

Battery challenges	2D materials	2D material properties	2D solution	Refs.
Dendrite growth	g‐C_3_N_4_	Uniform nanopores	Guides uniform plating	[77]
SEI instability	2D Mxene/ g‐ C_3_N_4_	Chemical stability	Forms Artificial SEI	[78]
Diffusion barrier	Graphene	Expanded interlayer	Fast Li^+^/Na^+^ sodiation highways	[79]
Volume expansion	2D (Py‐COF)	Low crystallinity (defects)	Buffers 80% sulfur swelling	[80]
Insulating sulfur	Graphdyiene	High electrical conductivity	Enables fast electron access	[81]
Slow redox kinetics	2D MoS_2_	Active edges	Catalyze SRR/ORR	[82]
Weak PS trapping	2D siloxene	Polar sites	Strong adsorption	[83]
Polysulfide dissolution	2D Mxene	Hydrophobic layer	Prevents shuttle effect	[84]
Mechanical stability separator	2D HUST‐1	High tensile strength	Reinforcement prevents tearing	[85]
Ion crossover	boron nitride	Tunable interlayer	Blocks anions, sieves M^+^	[86]
Low ionic conductivity	Phosphorene	Anisotropic ion‐transport pathways	Fast Li^+^/Na^+^ transport	[87]
Thermal runaway	boron nitride	High thermal conductivity	Heat dissipation	[88]
Poor electrolyte wetting	2D COF‐TpPa‐SO_3_​H	Hydrophilic channels	Complete electrode wetting	[89]
High electrolyte viscosity	2D CoFe‐BTC	2D nanochannels, surface‐functional regulation,	Enhances ion mobility conductivity	[90]

## 4 Classes of 2D Materials for MSBs

**FIGURE 3:**
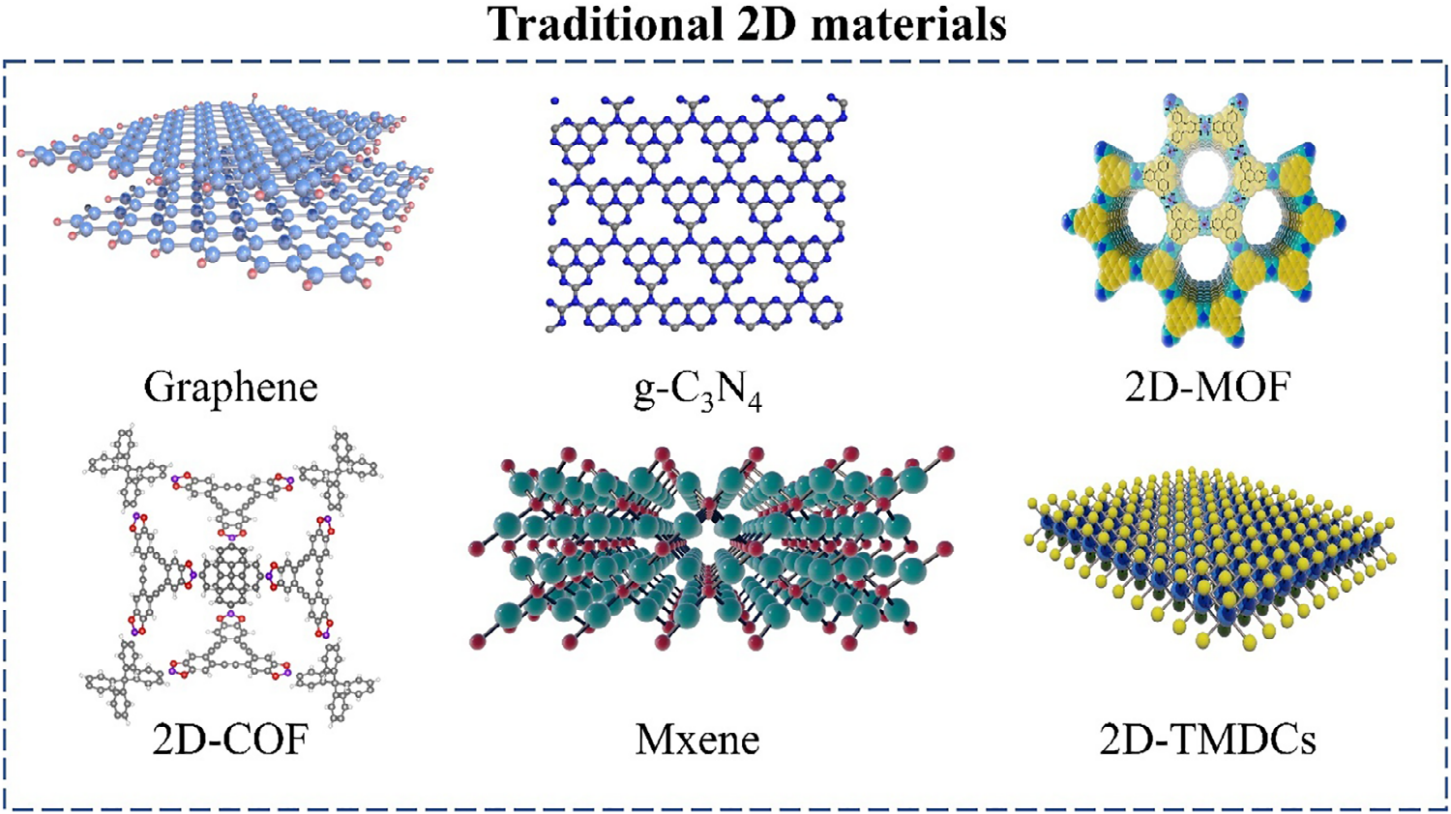
Graphical representation of traditional materials, including graphene, g‐C_3_N_4_, 2D‐ MOF, 2D‐COF, MXene, and 2D‐TMDCs that are utilized in MSB components.

2D materials improve the performance of MSBs by addressing key challenges, including poor conductivity, dendrite formation, and the shuttle effect. These materials are broadly categorized into traditional and next‐generation classes based on their structural evolution and functional versatility. Traditional 2D materials, such as graphene, g‐C_3_N_4_, MXenes, 2D MOFs, 2D COFs, and 2D‐TMDCs, offer high physicochemical properties, including conductivity and mechanical stability (Figure 3). In contrast, next‐generation 2D materials, such as GDY, phosphorene, and borophene, introduce directional charge transport, higher binding energies and tunable mechanical properties, which drive MSB performance. In the following section, we discuss traditional catalysts and explore next‐generation materials.

### 4.1 Traditional Materials

In 2004, Geim and Novoselov pioneered a revolutionary class of atomically thin 2D graphene, offering a new paradigm for energy storage applications [91, 92, 93]. Graphene, composed of a single‐atom‐thick hexagonal carbon lattice, exhibits exceptional physicochemical properties. These include ultra‐high carrier mobility up to 10^4^ cm^2^ V^−1^ s^−1^, excellent mechanical strength (1000 GPa Young's modulus), and an exceptional surface area (2630 m^2^ g^−1^). This performance arises from its sp^2^‐hybridized carbon network with a bond length of 0.142 nm and an interlayer spacing of 0.335 nm [94, 95, 96]. Graphene plays crucial roles in MSBs owing to its excellent electrical conductivity and structural robustness. The delocalized π‐electrons in its carbon lattice enable superior charge transport, improving the redox kinetics. For example, Chen et al. integrated sulfur into graphene matrices and enhanced the sulfur conductivity. The inherent porosity of graphene supports high sulfur loading, thereby improving sulfur utilization and mitigating the dissolution of polysulfides [39]. Additionally, the high surface area of graphene serves as an inorganic filler, promoting the formation of stable SEI layers that resist moisture, maintain electrolyte integrity, enhance ionic conductivity, and suppress parasitic reactions. Moreover, graphene‐containing electrolytes offer broad electrochemical stability windows, enabling operation across a wide range of temperatures and voltages and reducing the rate of decomposition during cycling. Functional groups on the graphene edges (─OH, ─COOH, and C─O─C) further enhance the ionic conductivity [97, 98, 99]. Graphene is also used to modify separators because of its (1) high electrical conductivity, which accelerates polysulfide redox reactions, (2) excellent thermal conductivity (∼5000 W m^−1^ K^−1^), which prevents thermal runaway, and (3) tunable pore volume and size, which help to sieve polysulfides. Additionally, the mechanical strength of graphene (>100 GPa tensile strength) accommodates volume changes during cycling, thus enhancing its structural stability [99, 100].

Beyond carbon‐based systems, nitrogen‐rich layered structures such as graphitic carbon nitride (g‐C_3_N_4_) offer additional anchoring chemistry for polysulfides. g‐C_3_N_4_ is a layered structure, composed of triazine (g‐C_3_N_4_) and tri‐s‐triazine/heptazine units interconnected by planar amino groups [101]. Its lamellar structure is held together by weak van der Waals (VDWs) forces, providing elasticity and preserving structural integrity. Each layer contains sp^3^‐hybridized nitrogen atoms that form covalent bonds with carbon, whereas sp^2^‐hybridized nitrogen atoms carry lone‐pair electrons that serve as chemically active sites [102, 103, 104]. Its high nitrogen content (up to 57.1 wt. %), which includes pyridinic, graphitic, and bridging nitrogen, offers abundant anchoring sites for polysulfides, thereby enhancing their binding energy [101]. Abdulmalek et al. demonstrated that pyridinic nitrogen in g‐C_3_N_4_ effectively binds Li^+^, immobilizes lithium polysulfides (LiPSs), and accelerates their redox kinetics [104]. The high surface polarization of g‐C_3_N_4_ also alters the molecular configuration of adsorbed LiPSs, further enhancing their redox activity [105, 106, 107]. The Angstrom‐scale pores (∼3 Å) of g‐C_3_N_4_ selectively allow metal‐ion passage and block soluble polysulfides, mitigating the shuttle effect [105]. Additionally, the VDW forces between the layers provide strong interlamellar binding, while the elastic nature of g‐C_3_N_4_ (210‐320 GPa) serves as a buffer matrix to accommodate volume expansion during plating and stripping cycles. Its interlayer spacing of ∼0.319 nm also facilitates improved interactions with electrolytes, boosting ionic transport and conductivity [108, 109]. Moreover, the aromatic heterocyclic rings in g‐C_3_N_4_ framework contribute to its thermal stability, allowing it to withstand temperatures of up to 600°C without degradation [105]. g‐C_3_N_4_ also demonstrates strong mechanical properties, namely a Young's modulus of 21.6 GPa, which is significantly higher than that of lithium metal (∼7.8 GPa) [40, 41]. Their Young's modulus allows it to modify the metallic anode, which leads to suppressing dendrite formation.

Another significant class of 2D materials is MXene, generally represented as M_n+1_AX_n_, where M represents the transition metal, A represents an element from the 13th or 14th group, and X can be a nitrogen or carbon atom. These materials are derived from the selective etching of MAX phases and classified based on the n value: M_2_AX (211), M_3_AX_2_ (312), and M_4_AX_3_ (413). The etching process exposes multiple polar functional groups on their surfaces, including oxygen (═O), hydroxyl (─OH), and fluorine (─F), which enhances polysulfide binding in MSBs. It is also worth noting that the tunable surface chemistry of MXenes contributes to their exceptional electronic, physicochemical, and optoelectronic properties. For example, Ti_3_C_2_T_a_ exhibits remarkable mechanical strength (Young's modulus 0.33 ± 0.03 TPa, tensile strength 15.4 GPa) and outstanding electrical conductivity (up to 20 000 S cm^−1^), which are crucial for suppressing dendrite growth and improving cathode electron transport [110, 111, 112, 113, 114, 115, 116, 117].

In addition to MXenes, 2D MOFs represent another class of hybrid materials composed of organic ligands bonded to central metal ions [118, 119]. These materials are characterized by bond energies ranging from 60 to 350 kJ mol^−1^. Owing to the choice of central metal nodes, including alkaline earth metals, transition metals, and lanthanides, and organic ligands with multidentate molecules such as N‐ or O‐donor atoms (pyridyl, carboxylates, and polyamines), 2D MOFs exhibit unique physicochemical and optoelectronic properties. For example, 2D MOFs exhibit high surface areas (up to 3679 m^2^ g^−1^), which accelerates sulfur conversion and reduces polarization [120, 121, 122, 123, 124]. In general, large pores accommodate more sulfur but struggle to confine polysulfides, whereas small pores effectively impede polysulfide shuttling but limit sulfur loading. This necessitates careful pore size engineering; 2D MOF pores can be tuned up to 9.8 nm by altering organic and metal‐containing units, allowing them to accommodate large amounts of sulfur (∼0.68 nm) [125, 126, 127]. Numerous studies have confirmed that internal voids in 2D MOFs act as molecular sieves to immobilize polysulfides, benefiting from intergranular channels between layers [128, 129]. In addition, various polar functional groups in organic ligands bind with polysulfides to confine them and mitigate the shuttle effect by reducing their mobilities. 2D MOFs high surface area also exposes abundant active sites, enhancing polysulfide adsorption and conversion kinetics [122]. Despite their advantages, the intrinsically poor electrical conductivity of 2D MOFs restricts their effectiveness as matrix hosts in sulfur batteries. Despite these limitations, 2D MOFs have been employed as electrolyte additives and separator modifications. This helps in enhancing ion transport and improving interfacial stability. The pore sizes of 2D MOFs were engineered to specific dimensions to facilitate selective permeability. This tunability allows the separator to restrict the migration of large polysulfide species while permitting the passage of metallic ions [130, 131, 132, 133, 134]. Their pore structure enables selective ion permeability, allowing cation transport while impeding polysulfide migration. Additionally, 2D‐MOFs serve as ion reservoirs and improve electrolyte ionic conductivity by reducing crystallinity, enhancing the overall battery performance [130, 131, 132, 133, 134].

Similar to 2D MOFs, 2D COFs are constructed by linking aromatic building units into layered, porous, and crystalline sheets with pronounced π–𝜋 stacked frameworks. These structural features provide ordered in‐plane pores, stacked interlayer channels, large exposed surface area, and chemically tunable active sites [80, 135, 136]. Compared with conventional porous hosts, 2D COFs offer a more accessible and continuous interface owing to their thin lamellar morphology, which promotes faster ion transport, improved electrolyte infiltration, and more efficient contact with sulfur species [137, 138, 139]. In addition, their frameworks can be rationally tailored through linker and node selection to introduce polar functionalities, such as imine, triazine, carbonyl, and other nitrogen‐rich moieties, thereby enhancing chemical affinity toward polysulfides and strengthening their confinement during cycling. The ultrathin 2D COF nanosheets expose a high density of pore walls and functional groups to the electrolyte, enabling polysulfides to be adsorbed and converted across a broad active interface rather than only at isolated buried sites. Their ordered nanopores also provide sufficient space for sulfur loading and accommodate volume changes during charge‐discharge processes, while the short diffusion pathways across thin layers facilitate Li^+^/Na^+^ transport and accelerate redox kinetics [137, 140]. On the anode side, 2D COFs regulate ion flux and homogenize lithium deposition, thereby suppressing uneven plating and improving interfacial stability [141, 142]. When applied to separators, ultrathin 2D COF coating layers combine physical sieving with chemical trapping of polysulfides. Their intrinsic lamellar architecture creates a tortuous yet ordered transport pathway that still permits Li^+^/Na^+^ migration, while larger or more strongly interacting polysulfide species are effectively adsorbed or catalytically converted before reaching the anode. As a result, the separator becomes an active functional interlayer rather than a passive barrier [138]. For example, Ye et al. employed a 2D covalent triazine framework (CTF) containing benzene derivatives and triazine rings, where the polar heteroatom‐rich structure provided strong chemical anchoring toward polysulfides together with lithiophilic interactions. This design effectively suppressed polysulfide shuttling, improved sulfur utilization, and reduced self‐discharge [85].

Exploration of 2D transition metal dichalcogenides (TMDCs), such as titanium disulfide (TiS_2_) and molybdenum ditelluride (MoTe_2_), has gained significant attention in MSB systems. TMDCs are typically layered materials with the formula MX_2_, where M is a transition metal, and X is a chalcogen (S, Se, or Te). In this configuration, a transition metal layer is sandwiched between two chalcogen layers, which imparts properties such as a direct bandgap, carrier mobility, capacity, open‐circuit voltage, and electrochemical activity [143, 144, 145, 146, 147]. 2D‐TMDCs exist in either layered (groups 4–7) or nonlayered (groups 9–10) forms, depending on the transition metal group, and their properties are tailored accordingly. A key advantage of the layered VDW structure is its ability to facilitate metal‐ion intercalation, thereby lowering the ion transport energy barriers and suppressing dendrite formation [147, 148, 149]. These materials adopt various stacking modes, including hexagonal (2H), trigonal (1T), and rhombohedral (3R), each associated with distinct electronic conductivities [150, 151]. In a recent study, Chhowalla et al. synthesized 1T‐phase MoS_2_ with octahedral coordination, which exhibited metallic conductivity, whereas the 2H phase was semiconducting. The 1T phase is approximately 10 times more conductive than the 2H phase and is often compared to graphene in terms of electron transport. These 1T MoS_2_ nanosheets accelerated the redox kinetics and electron transfer during cycling. Furthermore, MoS_2_ exhibits strong interactions with polysulfides through metal‐sulfur bonding, which suppresses polysulfide dissolution, enhances sulfur utilization, and improves Li^+^‐transport [152]. TMDCs often have large interlayer spacings (>6 Å) and weak VDW interactions in the out‐of‐plane direction. These large interspacing and negatively charged X_2_^−^ ions enable them to accommodate substantial numbers of metallic ions (such as Na^+^, K^+^ and Mg^2+^) in their structures. Combined with their chemical robustness, TMDCs are utilized in cathode matrices, separator modification, and dendrite suppression [153, 154, 155]. Additionally, d‐band center theory reveals that metal sites with higher d‐band centers have stronger binding to adsorbates. This leads to stronger polysulfide‐catalyst interactions and lower reaction barriers, thereby enhancing the battery efficiency. Furthermore, the high thermal stability and mechanical strength of 2D‐TMDCs arise from the strong covalent bonds between transition metals and chalcogen atoms. These features enable 2D‐TMDCs to maintain their structural integrity under harsh cycling [145, 156, 157].

Although traditional 2D materials, such as graphene, MXenes, g‐C_3_N_4_, MOFs, and TMDCs have gained considerable advancements in MSBs, their effectiveness is often constrained by inherent limitations, such as poor polarity, large/uneven pores, and low polysulfide affinity. For instance, graphene suffers from a zero bandgap, whereas g‐C_3_N_4_ and 2D‐MOFs lack electrical conductivity, and 2D‐TMDCs suffer from oxidative degradation. These constraints have catalyzed the exploration of a broader family of next‐generation 2D materials, many of which exhibit tunable bandgaps, enhanced chemical affinities for polysulfides, unique charge transport characteristics, and robust mechanical properties. Below, we examine next‐generation candidates that enhance the performance of MSBs.

### 4.2 Next‐Generation 2D Materials

**FIGURE 4:**
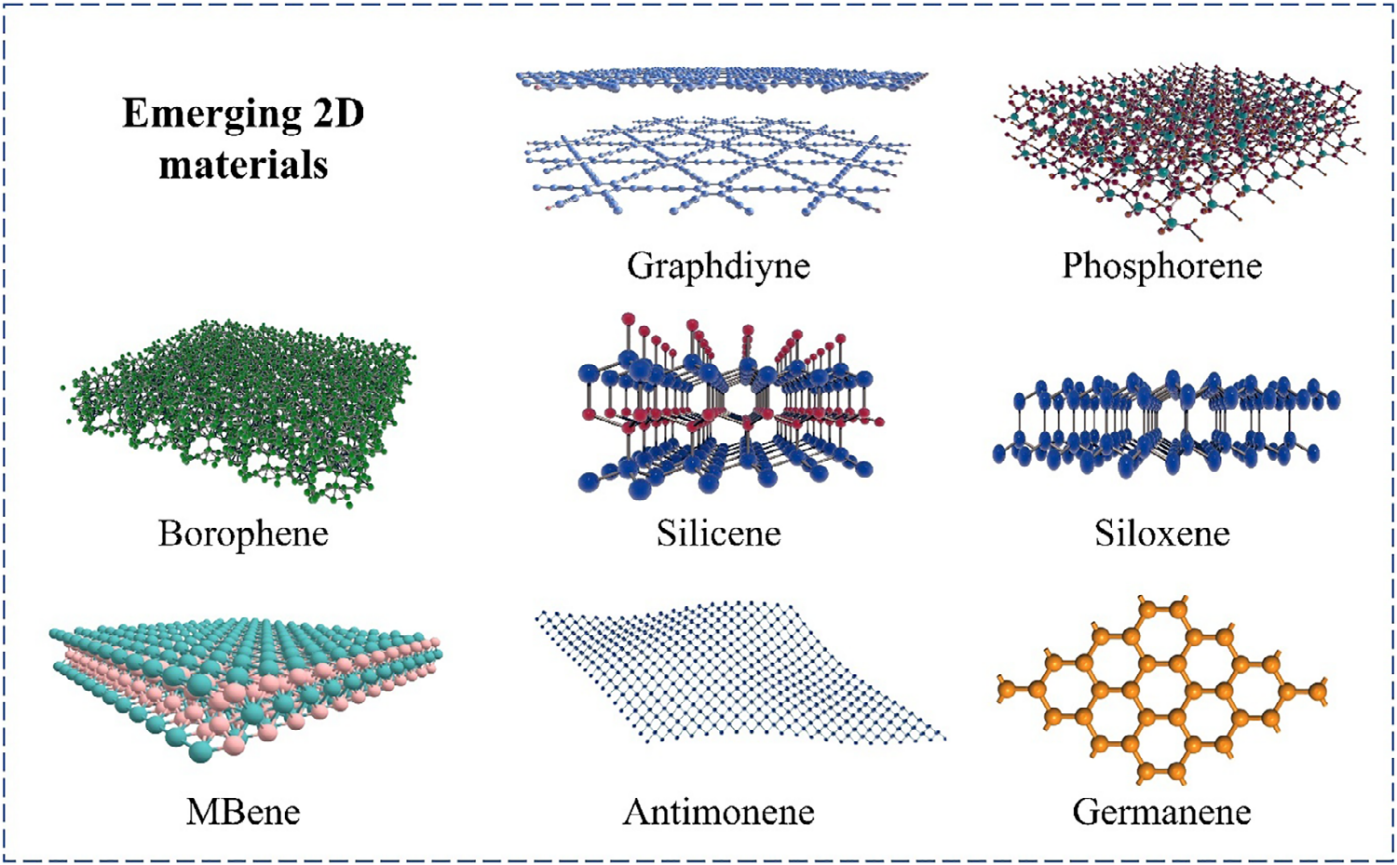
Structural representations of emerging 2D materials, including graphdiyne, phosphorene, borophene, siloxene, silicene, MBene, antimonene and germanene that are utilized in different components of MSBs.

To date, more than 100 members of the 2D family have been discovered and characterized, and this number is rapidly increasing. The extraordinary properties of these newly discovered 2D materials are driving scientific innovations towards new advancements. These materials exhibit exceptional electronic, mechanical, and structural properties that have driven innovations in energy storage. Figure 4 shows emerging 2D materials such as GDY, germanene, silicene, MBene, antimonene, siloxene, borophene, and phosphorene that are utilized in different components of MSBs. Among emerging 2D materials, GDY has attracted widespread attention [158, 159]. Researchers led by Li et al. at the University of Manchester synthesized GDY by altering the atomic arrangement of graphene carbon. The interaction between the atomic hydrogen and graphene carbon atoms leads to the formation of diacetylene units (─C≡C─) [160, 161, 162]. These diacetylene linkages impart sp/sp^2^ hybridization, creating uniformly distributed pores and exceptional charge mobility (∼10^5^ cm^2^ V^−1^ s^−1^) [163, 164]. With a tunable bandgap of 0.5–1.1 eV, GDY improves charge transport and mechanical robustness (Young's modulus: 470–580 GPa), aiding in accommodating the volume changes in the sulfur cathode. Its π‐conjugated network interacts with polysulfides, suppressing the shuttle effects and enhancing the redox kinetics [165, 166]. In addition, computational simulations have shown that GDY anisotropy results from multiple Dirac cones. Consequently, the electron flow can be controlled in one direction, resulting in a higher conductivity than graphene [167, 168]. Unlike graphene, the butadiyne linkages between hexatomic benzene matrices provide uniformly distributed pores in GDY, making it an attractive candidate for cathode matrices [168].

Another promising 2D material is phosphorene, derived from black phosphorus. Since 1669, white phosphorus has been studied extensively for various applications. However, its instability prompted Bridgman in 1914 to synthesize black phosphorus by subjecting white phosphorus to a high pressure of 1.2 GPa and an elevated temperature of 200°C. As a result, phosphorene was added to the 2D families in 2014 [169]. In phosphorene, intralayer bonding is characterized by phosphorus atoms (sp^3^ hybridization), which leads to the formation of three covalent bonds with adjacent phosphorus atoms. Consequently, the bonding between the two phosphorus atoms is facilitated by the shared lone pairs of electrons associated with each atom. The out‐of‐plane bonds in phosphorene, oriented at an angle of 45° with respect to the plane, create a unique puckered configuration that contributes to its anisotropic behavior. This results in robust interlayer connectivity and forms a stable orthorhombic crystal structure. Phosphorene possesses strong in‐plane anisotropy and contains both edge and terrace sites. The interplay between sp^3^ hybridization and the puckered structural arrangement displays a distinctive honeycomb structure, which is different from the flat lattice observed in graphene. Owing to this unique feature, it exhibits notable properties, such as carrier mobility up to 1000 cm^2^ V^−1^ s^−1^, mechanical strength with Young's modulus ranging from 44 to 166 GPa, Poisson's ratio from 0.4 to 0.93 and thermoelectric properties. Furthermore, phosphorene exhibits ambipolar semiconductor behavior, and its bandgap can be tuned from 0.3 eV (single layer) to 2.0 eV (bulk phosphorene), making it an ideal candidate for separator and cathodic materials [170, 171, 172, 173]. Moreover, computational simulations have shown that the binding energies of various LiPSs with phosphorene layers range from 1 to 2.5 eV, which is greater than that of a carbon network (∼0.5 eV). This higher binding energy indicates that phosphorene is more effective at immobilizing polysulfides, reducing the polysulfide dissolution issues [56, 174, 175]. Consequently, phosphorene‐based materials enhance the cycling stability and overall performance of battery systems.

Although graphene exhibits unique properties, its zero bandgap limits its applications. To overcome these limitations, Vogt et al. employed the molecular beam epitaxy (MBE) method in an ultrahigh vacuum (UHV) setting to deposit pure Si on silver (Ag (111)). Silicene was synthesized in 2012 with a buckled honeycomb lattice structure [176, 177]. The buckling facilitates stronger ion‐electron coupling, suppressing dendrite formation and promoting homogeneous metal deposition. For instance, electrons in the buckled state of silicene are more delocalized, producing a smaller inter‐electron repulsive energy and leading to partial sp^3^ hybridization. The silicene mixed sp^3^/sp^2^ hybrid bonds endow unique properties, including high charge‐carrier mobility and excellent electrical conductivity, enabling it to function as a conductive scaffold or polysulfide barrier [178, 179, 180, 181, 182]. In this context, Shao et al. calculated silicene electron and hole mobilities using DFT calculations and found them to be 2.57 × 10^5^ and 2.22 × 10^5^ cm^2^ V^−1^ s^−1^, respectively [183]. Although the energy gap of silicene (1.9 meV) is similar to that of graphene (0.02 meV), the silicene bandgap is tuned by various dopants, positioning silicene as an ideal candidate [184].

**FIGURE 5:**
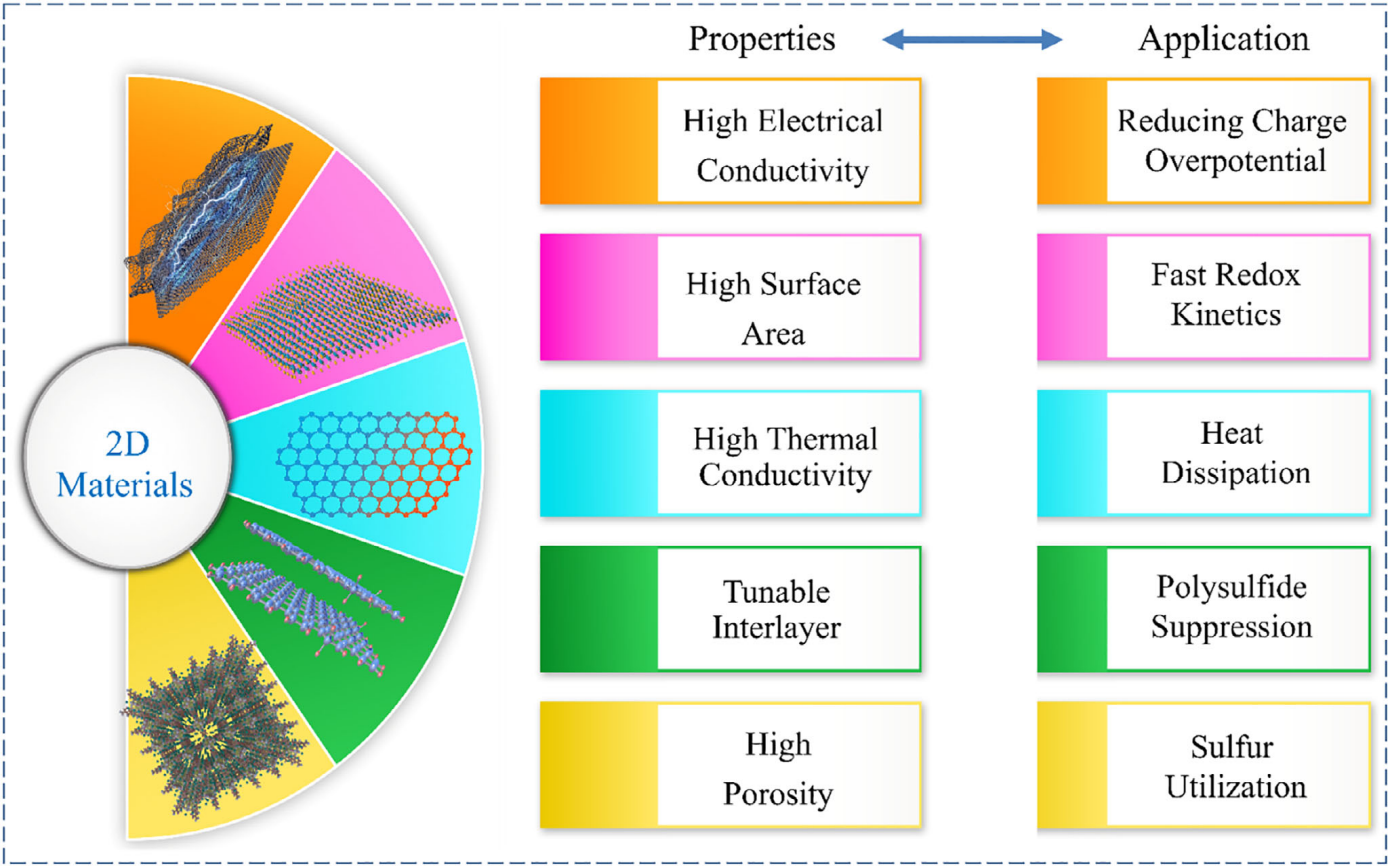
2D materials properties, including high electrical conductivity, high surface area, high thermal conductivity, tunable interlayer and high porosity, that results in reducing charge overpotential, heat dissipation, polysulfide suppression, and improving redox reaction, sulfur loading in MSBs components.

Siloxene, another silicon‐based 2D material, features silicon atoms in a planar structure with oxygen‐containing functional groups, such as Weiss‐type, Kautsky, and chain‐like structures [185, 186]. The siloxene structure is connected via Si‐O bonds, with hydrogen and hydroxyl groups on its surface in varying configurations. This structural arrangement contributes to its distinctive properties, setting it apart from its counterparts, such as silicene and graphene. For example, Siloxene has a high surface area, which adsorbs and converts polysulfides effectively. Similar to Silicene, Siloxene sp^3^ hybridization contributes to its thermal stability, allowing it to maintain structural integrity under varying temperature conditions [187]. Furthermore, Siloxene is utilized as an anode material, where its layered structure accommodates volume expansion during lithiation/delithiation, preventing electrode pulverization. Its high theoretical capacity and low Li^+^ diffusion barrier enable fast ion transport and high energy density. Additionally, its tunable surface chemistry promotes SEI formation, enhancing cycling stability and rate performance. Owing to their distinctive furrowed 2D configuration, Siloxene exhibits high chemical resistance and superior mechanical properties, making it an inevitable contender in MSBs [188, 189]. Figure 5 displays the highlighted properties of emerging 2D materials that are used in MSB components. Since the discovery of graphene, boron‐based 2D materials have attracted significant attention because of the chemical similarity between boron and carbon. In 2014, Piazza et al. successfully synthesized Borophene, a monolayer boron allotrope with unique structural and electronic properties [42, 190]. Borophene exhibits high electrical conductivity and structural stability owing to its anisotropic metallic bonding and rich polymorphism, including β_12_, X_3_, δ_6,_ and 2‐Pmmn configurations. These allotropes consist of intricate triangular and hexagonal motifs with sp^2^ hybridization, resulting in exceptional charge transport behavior. Notably, the 2‐Pmmn phase exhibits high lattice thermal conductivity (∼75.9 W m^−1^ K^−1^ in the zigzag direction and ∼147 W m^−1^ K^−1^ in the armchair direction), which is beneficial for thermal management in MSBs. Furthermore, Borophene features a negative Poisson's ratio and a high anisotropic Young's modulus (up to 280 GPa in the armchair direction), allowing it to buffer mechanical strain and maintain the electrode integrity during repeated volume changes [190, 191, 192, 193]. Combined with improved redox kinetics, sulfur utilization, and mechanical durability, borophene serves as a promising sulfur host or conductive scaffold in MSBs. For comparison, properties of various 2D materials were summarized in Table 2.

**TABLE 2:** Physicochemical properties of various 2D materials.

Catalyst	Surface area (m^2^/g)	Electrical conductivity (S/cm)	Thermal conductivity (W/m·K)	Mechanical properties (GPa)	Refs.
Graphene	∼2630	∼1 × 10^6^	∼5000	E = ∼1100 σ_t_ = ∼130	[100, 201]
g‐C_3_N_4_	∼2500	∼10^−11^	∼3.5	E = ∼21.6 σ_t_ = ∼30	[40, 41, 202, 203, 204]
2D MOF	3679 Zn+ dodeca‐chloro‐corenene	∼40 (Ni_3_(HITP) _2_)	∼0.25 (Ni_3_ (HITP)_2_)	E = 3–5 σ_t_ = ∼3–4	[105, 124, 205, 206, 207, 208]
MXene (Ti_3_C_2_T_x_)	∼517	∼1 × 10^4^	∼55	E = ∼500 σ_t_ = ∼15	[11, 47, 110, 114, 209, 210, 211]
TMDCs	Low	10‐100 (1T),	∼23 (MoS_2_)	E = ∼270 (WSe_2_) σ_t_ = ∼16 (WS_2_)	[47, 126, 212, 213, 214]
GDY	>2600	∼100	Low (∼1‐5)	E = ∼412 σ_t_ = 18‐24	[215, 216, 217, 218]
Phosphorene	>2600	∼100	13‐30	E = 44–166 σ_t_ = 8‐18	[219, 220, 221]
Silicene	Large	10^2^‐10^3^	∼9.4	E = ∼60–80 ×10^4^ σ_t_ = ∼24	[222, 223, 224]
Borophene	Large	∼10^4^	75‐147	E = 150–350 σ_t_ = ∼23	[47, 191, 192, 225]
MBene	High	∼10^4^‐10^5^	150‐200 (H type)	E = 1.1–1.9 (Cr_4_B_6_) σ_t_ = 15.8	[226, 227]
Germanene	Large	∼10^3^	∼2.4	E = 36–42 σ_t_ = ∼6	[223, 228, 229]
Antimonene	High	160	∼24	E = 15–55 σ_t_ = 5–7.5	[185, 230]

The germanene analog of graphene was first synthesized in 2014 by Guy Le Lay and colleagues. Unlike graphene, germanene lacks a VDW layered structure and is typically grown on metallic substrates as an adlayer [194]. It possesses a mixed sp^2^/sp^3^ hybridization and a pronounced buckled structure owing to the larger atomic radius of germanium. This buckling structure facilitates bandgap tunability, which overcomes the graphene zero‐bandgap limitation and enhances intrinsic carrier mobility. In MSBs, these electronic characteristics allow germanene to function as a highly conductive matrix or interfacial layer, thereby enabling efficient electron transport. Germanene's chemically active surface sites serve as strong anchors for polysulfide species, thereby suppressing the shuttle effect and stabilizing the electrode‐electrolyte interface [195, 196, 197, 198, 199, 200].

MBenes were predicted in the late 2010s and augmented into the 2D family by selectively removing the “A” element from layered MAB phases (M_n+1_AlB_n_, n = 1–3). MBenes are considered the mirror notation of MXenes with general formula M_x_B_y_ where M denotes a transition metal (Co, Cr, Fe, Hf, Ir, Mn, Mo, Nb, Ni, Os, Pd, Sc, Ta, Tc, Ti, V, W, Y, Zr) and B signifies Boron with remarkable electrical, mechanical and electrochemical properties [226]. Similar to MXenes, MBene layers form strong covalent M‐B bonds that coexist with metallic M‐M interactions, forming hexagonal/orthorhombic lattices with anisotropic in‐plane stiffness. Consequently, MBene typically exhibits metallic conductivity, high carrier density, robust mechanical properties, and good thermal transport, which are advantageous for MSBs [227]. The transition‐metal sites and tunable surface terminations of MBenes provide strong chemical interactions with LiPSs, while their metallic frameworks promote rapid charge transfer across the sulfur cathode or interlayer. In addition, their layered architecture and adjustable surface chemistry regulate ion transport and suppress polysulfide migration [231]. Further, MBenes are used as separators/interlayers owing to their polysulfide anchoring ability, and they accelerate the chain‐shortening reaction (S_8_ → S_x_^2−^ → Li_2_S/Na_2_S) [232]. The physicochemical properties of MBenes tune polysulfide affinity, improve charge transport, and provide high mechanical robustness, making them compelling 2D candidates for shuttle suppression and interfacial stabilization in Li–S and Na–S cells [233].

Antimonene, a member of the 2D pnictogen family, has emerged as a promising material for energy storage following the success of phosphorene. The most stable β‐phase of Antimonene (space group R3m) forms a buckled hexagonal lattice with high electrical conductivity (up to 1.6  × 10^4^ S m^−1^) and has a tunable bandgap up to ∼2.28 eV [234, 235, 236]. This bandgap is tuned by controlling the degree of atomic buckling within a single layer. In the context of MSBs, antimonene offers multiple functional advantages: (1) its high electrical conductivity enhances redox kinetics and rate performance, (2) its moderate thermal conductivity (∼24 W m^−1^ K^−1^) supports thermal regulation and (3) its mechanical strength (Young's modulus ∼55 GPa, bulk modulus ∼42 GPa) provides structural resilience during the expansion of sulfur volume. Antimonene's surface also exhibits strong affinity for LiPSs, enabling efficient sulfur utilization and reducing polysulfide dissolution [234, 237, 238, 239]. These combined physicochemical, electrochemical, mechanical, and thermal properties make antimonene a multifunctional candidate for MSBs. Together, these 2D materials offer unique physicochemical properties and tunable functionalities that address the critical limitations of MSBs.

## 5 Applications of 2D materials in M–S Batteries

2D materials have revolutionized the landscape of MSBs by addressing daunting challenges, such as the insulating nature of sulfur, volume expansion, polysulfide dissolution, dendrite formation, and poor electrolyte stability. Owing to their ultrathin geometry, tunable surface chemistry, mechanical robustness, and high aspect ratios, 2D materials exhibit multifunctional advantages in various battery components, including sulfur cathodes, metal anodes, electrolytes and separators. Beyond their intrinsic properties, the performance of 2D materials can be further tailored through strategies such as single‐atom doping, heteroatom incorporation, defect engineering, and the construction of homo/heterojunctions. Among these, single‐atom catalysts anchored on graphene (SAC@graphene) have emerged as a particularly powerful approach. When integrated into sulfur cathodes, SAC@graphene structures perform a dual function: (i) strong chemisorption of polysulfides through metal‐sulfur (M‐S) or Li‐N/Na‐N coordination bonds and (ii) electrocatalytic acceleration of polysulfide conversion reactions. Further, the local coordination environment of SACs, particularly the M‐Nx moieties, plays a decisive role in determining polysulfide adsorption strength and catalytic activity. For example, Wan et al. embedded Co SAC in nitrogen‐doped graphene (Co‐N/G) serve as a bifunctional electrocatalysts that facilitate both the formation and decomposition of Li_2_S during discharge and charge, respectively; the S@Co‐N/G composite delivers a gravimetric capacity of 1210 mAh g^−1^ at 90 wt% sulfur loading, with a capacity fading rate of only 0.029% per cycle [240]. Similarly, among a series of M‐NG catalysts (M═Fe, Co, Ni, Ir, Ru), Zhang et al. identified Ni‐NG catalyst in favorable pathway for reversible polysulfide conversion, improving sulfur utilization across the cathode [241]. At the anode, atomically dispersed Co‐N_x_ sites within a nitrogen‐doped carbon framework tune the local electronic structure to create abundant lithiophilic nucleation centers, guiding uniform Li deposition and suppressing dendrite growth; CoNC anodes retained stable CE over 200 cycles at 8.0 and 10.0 mA cm^−2^, respectively, outperforming bare nitrogen‐doped graphene and Cu current collectors [242]. At the separator, Chen et al. utilized nitrogen‑doped graphene scaffolds hosting atomically dispersed Ni‐N_4_ centers (Ni@NG) to convert PP membrane into an active, conductive catalytic interface. The oxidized Ni sites on PP membrane act as strong polysulfide traps (S_x_^2−^⋯Ni─N bonding) and lower the free‑energy and decomposition barriers for Li_2_S_x_ conversion, thereby accelerating SRR/SOR and suppressing shuttling. Even at ultralow Ni loadings, Li–S cells equipped with Ni@NG‑modified separators exhibit excellent rate capability and long‑term durability, with a capacity decay of only 0.06% per cycle, illustrating SAC@graphene high electronic conductivity with a finely tuned M‐N_x_ chemisorption separator can stabilize sulfur chemistry over extended cycling [243]. At the electrolyte level, transition‐metal SACs on nitrogen‐doped graphene (TM‐NG, TM═Cr, Fe, Co) exhibit adsorption energies for soluble Na_2_S_n_ species that exceed those of common ether‐type electrolyte solvents. These results indicate that M‐N_x_ sites outcompete the electrolyte in binding polysulfides before they can shuttle, effectively reducing dissolved intermediate concentration in the electrolyte phase during cycling in Na–S batteries [244]. This strategy tailors the 2D properties and increases their efficiency when used in 2D components. With this insight, below section discusses emerging and traditional 2D materials with single atoms and heteroatoms in different battery components.

### 5.1 Hosts for S Cathode

**FIGURE 6:**
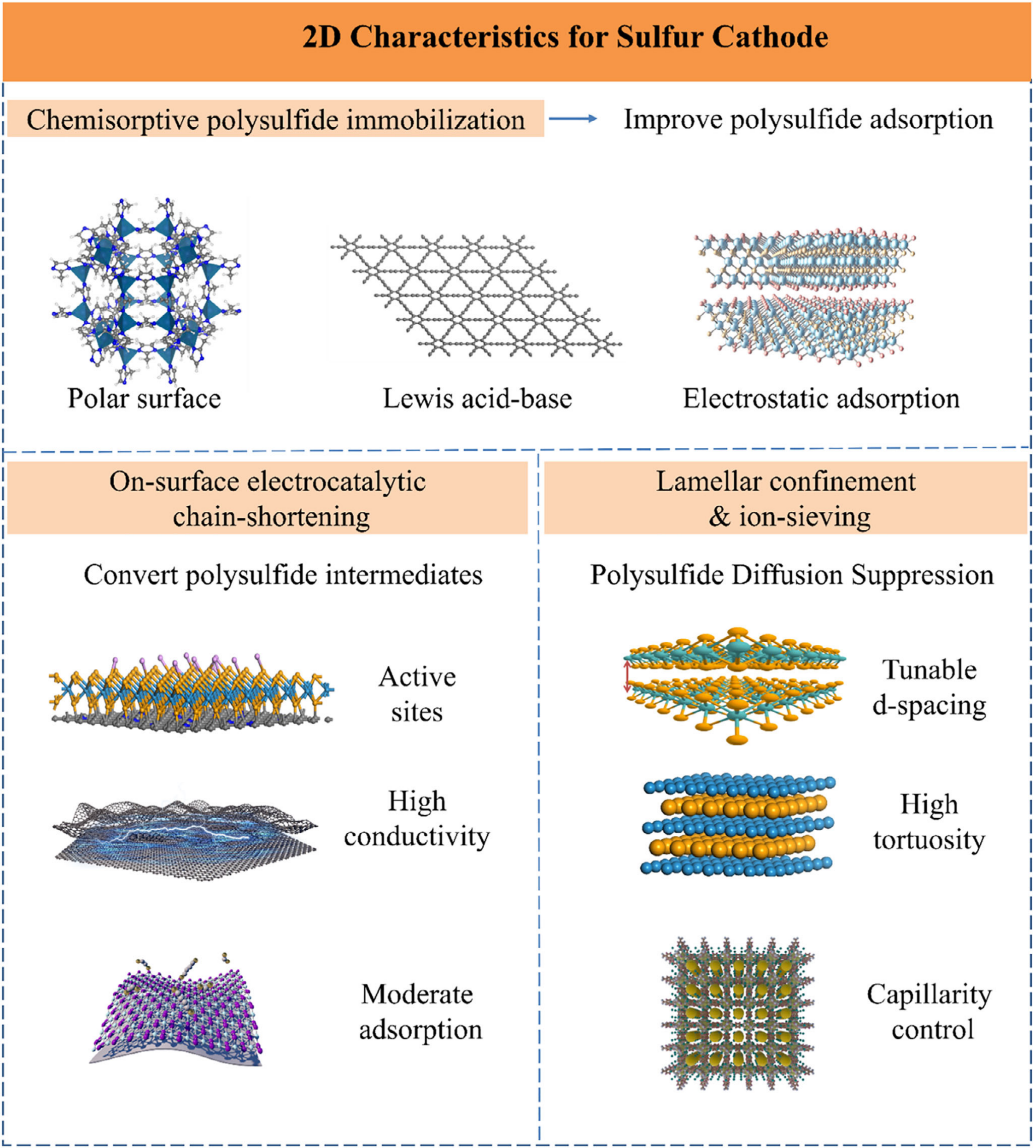
Schematic representation showing the characteristics of 2D materials in Li–S/Na–S cells. (Top) Chemisorptive polysulfide immobilization: polar surfaces and Lewis acid‐base sites (e.g., N/O/B‐functional groups, metal = O_x_ edges) and electrostatic interactions anchor Li_2_S_n_/Na_2_S_n_ to suppress shuttle; On‐surface electrocatalytic chain‐shortening: abundant active sites and high in‐plane conductivity (graphene/MXene/TMDCs) accelerate S_8_ → M_2_S_n_ → M_2_S conversion while keeping adsorption moderate for rapid release; Lamellar confinement & ion‐sieving: tunable interlayer spacing and high tortuosity confine polysulfides yet allow Li^+^/Na^+^ transport; capillary/wetting control in porous 2D frameworks improves electrolyte distribution. Collectively, these traits improve kinetics, CE, and cycling stability.

The sulfur cathode is the central component of MSBs, yet its intrinsically low electronic conductivity (∼5 × 10^−30^ S cm^−1^), the formation of soluble polysulfide intermediates, and volume fluctuations (∼80% for Li–S and ∼170% for Na–S) deteriorate electrochemical performance. To tackle these issues, 2D materials have emerged as advanced sulfur hosts because of their high conductivity, chemical tunability, and large surface area, enabling multiple, synergistic functions. In particular, 2D materials improve sulfur cathodes through (i) chemisorptive polysulfide immobilization, which involve three strategies such as polar surfaces, Lewis acid‐base interactions, and electrostatic adsorption. This helps to mitigate shuttle effect and retain active material (Figure 6); (ii) on‐surface electrocatalytic chain‐shortening, where abundant exposed active sites and efficient in‐plane charge transport along with selective adsorption accelerate polysulfide conversion kinetics, facilitating the transformation of long‐chain intermediates into short‐chain species and ultimately reducing polysulfide and enhance rate capability; and (iii) lamellar confinement/ion‐sieving, which involve stacked architectures with tunable interlayer spacing, high tortuosity and capillarity control physically suppress polysulfide diffusion while maintaining ion transport. With these insights, this section focuses on efficient 2D material hosts to improve the cathode performance of MSBs.

#### 5.1.1 Shuttle Suppression Strategies

**FIGURE 7:**
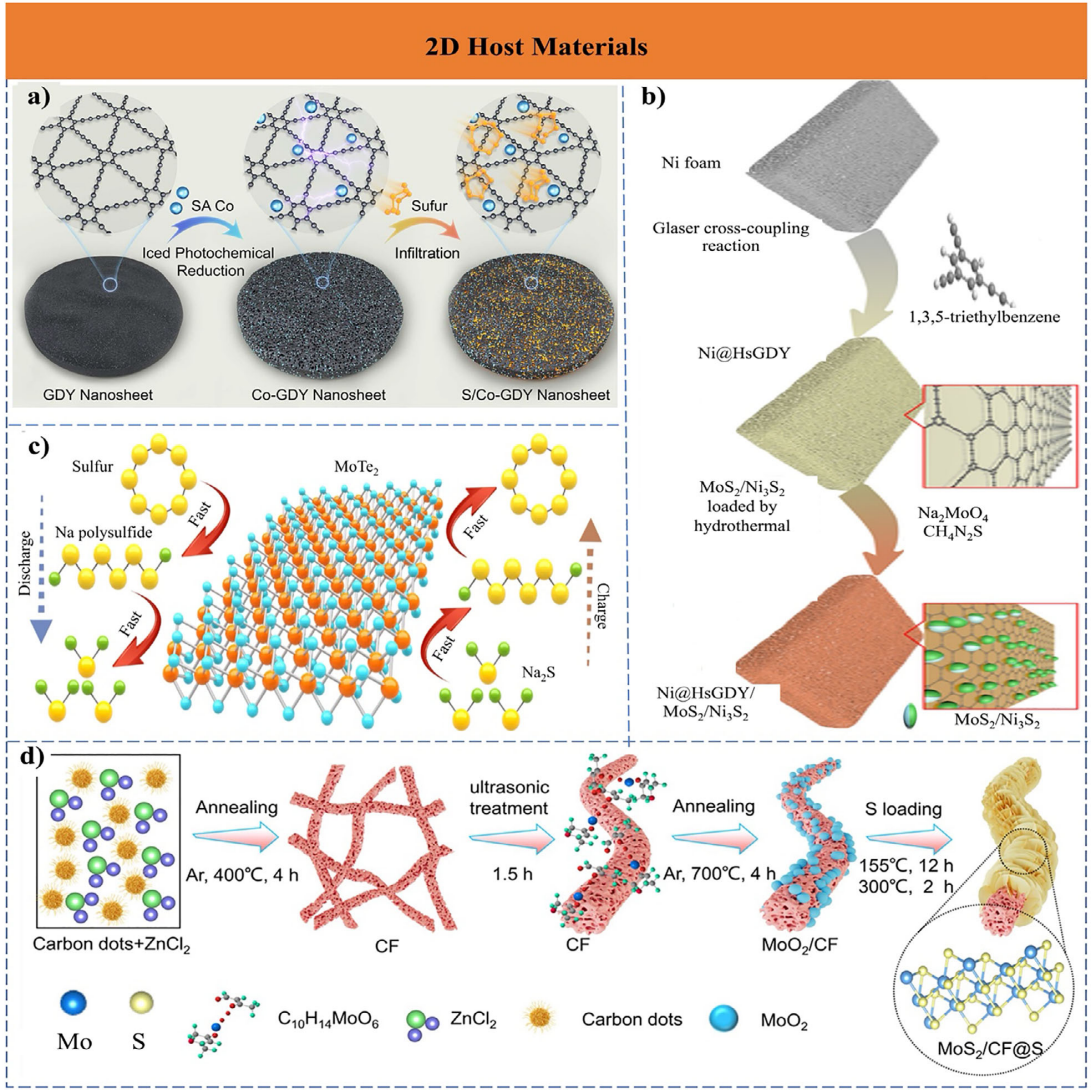
(a) Synthesis scheme of S/Co‐GDY nanosheet (reproduced with permission from ref. [81]. Copyright 2024, American Chemical Society); (b) graphical illustration of Ni@HsGDY/MoS_2_/Ni_3_S_2_. (reproduced with permission from ref. [254]. Copyright 2023, Science Partner Journal); (c) schematic illustration of MoTe_2_ synthesis (reproduced with permission from ref. [258]. Copyright 2023, Springer Nature); and (d) graphical illustration of preparation of MoS_2_/CF@S (reproduced with permission from ref. [76]. Copyright 2025, American Chemical Society).

**FIGURE 8:**
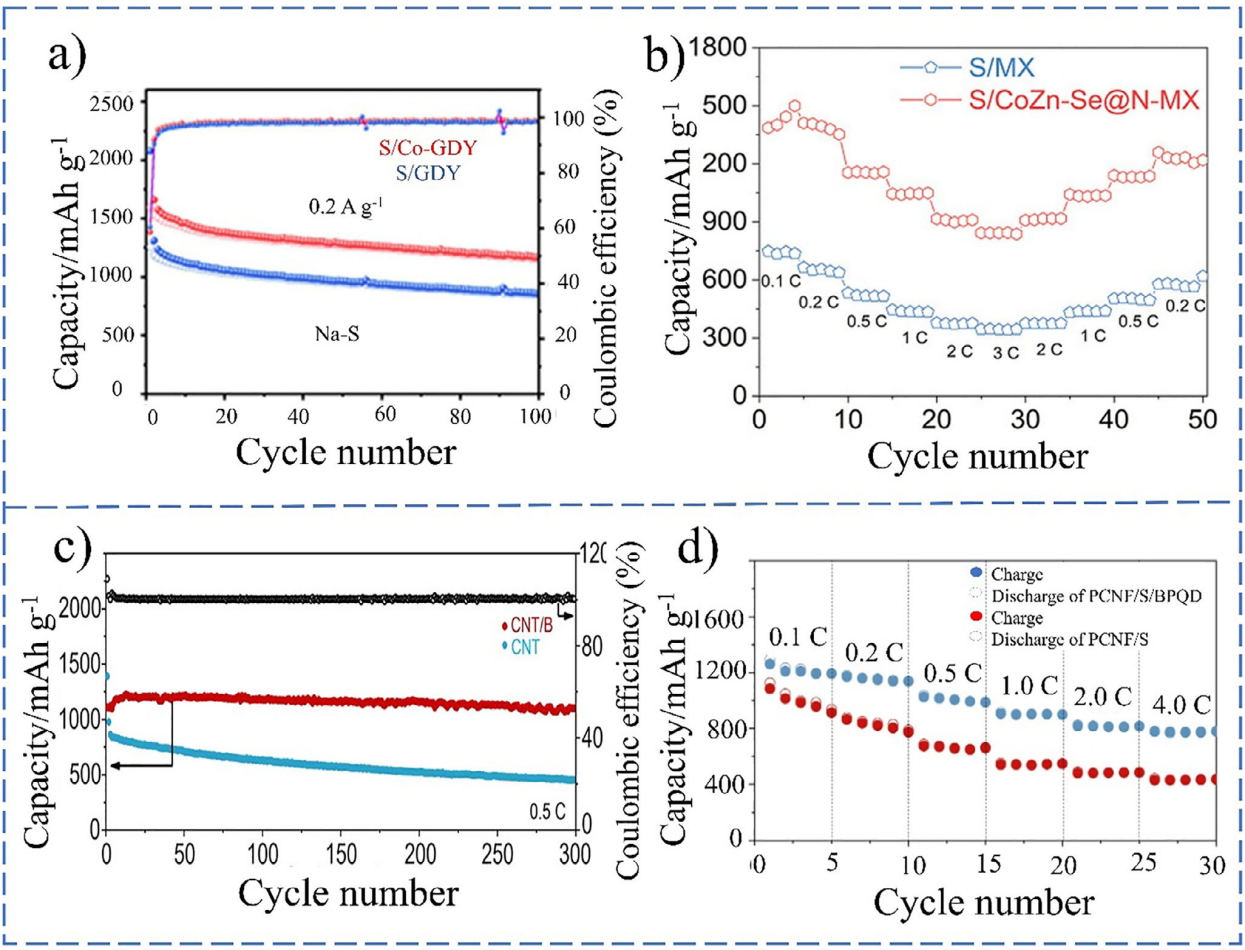
Electrochemical perfromance of (a)S/Co‐GDY and S/GDY cathodes for Na–S batteries (reproduced with permission from ref. [81]. Copyright 2024, American Chemical Society); b) S/Mx and S/CoZn = Se@N‐Mx cathodes for Li–S batteries (reproduced with permission from ref. [260]. Copyright 2021, Wiley); (c) CNT and CNT/B cathodes for Li–S batteries(reproduced with permission from ref. [261]. Copyright 2021, American Chemical Society); and (d) PCNF/S and PCNF/S/BPQD cathodes for Li–S batteries (reproduced with permission from ref. [273]. Copyright 2018, American Chemical Society).

The shuttle effect is a primary concern for MSBs. During the charge and discharge cycles, metallic ions react with cathodic sulfur and form polysulfides. This polysulfide diffuses into the electrolyte and reacts with the anodic ions, causing self‐discharge and deposition on the anodic surface, resulting in the loss of active material, capacity loss, reduced lifespan, and increased internal resistance. To mitigate the shuttle effect, researchers have explored various 2D cathodic matrices with high surface areas and conductivities [245, 246, 247]. Among 2D materials, carbon‐based materials, such as porous carbon, graphene, and reduced graphene oxide (RGO) have been explored as cathodic hosts for MSBs owing to their excellent conductivity and high porosity [248, 249, 250]. These materials reduce the shuttle effect by confining intermediates through chemical bonds, physical confinement, surface adsorption, and electrostatic interactions. For instance, Jia et al. infused sulfur moieties onto a graphene surface using a vapor‐infiltration technique. Sulfur covalently bonded to the graphene surface suppresses the formation of soluble polysulfides by generating insoluble S_2_^−^ and S_2_^2‒^ species. Owing to its high electrical conductivity and improved electrolyte wetting, the S@graphene framework inhibits the detrimental shuttle effect and volume fluctuations. The optimized sulfur cathode delivered an initial specific capacity of 1069 mAh g^−1^ and retained 492 mAh g^−1^ after 100 cycles at 0.1 C [251]. However, the weak physical interactions between nonpolar carbon structures and polysulfides remain a bottleneck [252]. To overcome these drawbacks, researchers have developed other carbon allotropes and integrated them with transition metals, single atoms, and MOFs. For example, to improve polysulfide adsorption and boost electrical conductivity, Li et al. synthesized GDY and incorporated ruthenium quantum dots (RuO_x_ QDs). Unlike other carbon allotropes, GDY features sp and sp^2^ hybridization, offering a vast surface area, high porosity, and excellent electrical conductivity. In addition, the alkyne‐alkene bonds in GDY modulate the metallic‐ion pathway, facilitating rapid kinetic reactions. Furthermore, the in‐plane cavities of GDY trap polysulfides and circumvent shuttle effects. In the S/RuOxQDs/GDY cathodic electrode, the unoccupied GDY π* orbital facilitated electron transfer from the Ru d orbital via π bonding. This strong d‐π orbital coupling regulates the Ru environment and promotes strong interactions with the sulfur atom (p‐orbital) via d‐p orbital coupling. Consequently, electrons are transferred to the cathode material. The activation of the S‐S bond resulting from the nucleophilic attack of the Ru atom increased LiPSs adsorption on the RuO_x_QDs/GDY/S catalyst. As a result, the RuO_x_QDs/GDY/S catalyst limited the shuttle effect and improved the specific capacity. Further, Li@RuO_x_QDs/GDY anodic electrode achieved a stable configuration owing to the strong affinity and displayed a cycle life over 8800 h without Li dendrite growth. In addition, Li@RuOxQDs/GDY||S@RuOxQDs/GDY full cells were tested under practical conditions with a lean electrolyte (E/S = 5 µL mg^−1^), a low N/P ratio of 1.4, and a high sulfur mass loading of 13.6 mg cm^−2^ at 0.05 C. The assembled cells delivered a high initial areal capacity of 15.8 mAh cm^−2^ and maintained an impressive areal capacity of 11.33 mAh cm^−2^ after 100 cycles, demonstrating excellent cycling stability. This exceptional stability might originate from the π backdonation from GDY sheet that regulates the d‐electron structure of the Ru centers. This interaction prevents the degradation of Ru atoms over prolonged cycles [253]. Similarly, Zhang et al. embedded a cobalt (Co) single atom on GDY sheets (Figures 7a and 8a). The diacetylene linkages in GDY regulate the Co chemical environment and alter the electron cloud distribution via d‐π orbital coupling. The strong P‐d orbital coupling between sulfur and Co atoms increased sulfur adsorption, and the porous GDY structure inhibited polysulfide dissolution [81]. GDY has an extended π‐conjugated carbon skeleton composed of butadiyne linkages and benzene rings, which facilitates polysulfide conversion [161]. To further increase polysulfide trapping,  Lu et al. hydrogenated GDY (HsGDY). In HsGDY, the P_x_‐P_y_ of the π/π* orbitals rotate perpendicularly to the diacetylene group, pointing toward the dopants and creating metal anchoring sites. Wang et al. utilized the Glaser cross‐coupling reaction to anchor molybdenum disulfide/nickel sulfide (MoS_2_/Ni_3_S_2_) on the HsGDY surface (Figure 7b). Interestingly, the Mo and Ni atoms anchored on the HsGDY surface exhibited less interfacial strain between MoS_2_/Ni_3_S_2_ and HsGDY. The porous structure of HsGDY enables fast Li_2_S adsorption, and the synergy of MoS_2_ and Ni_3_S_2_ in the fabricated electrode improved the capacity in the Li–S battery [254]. Conversely, to reduce the shuttle effect, inorganic materials have attracted considerable interest because of their polar functional groups. Their strong chemical bonds (covalent and ionic bonds) increase the interactions between the sulfur host and polysulfides, which substantially enhance the polysulfide anchoring ability, thereby inhibiting their diffusion ability [255, 256, 257]. For instance, Dey et al. proposed a 2D metallic carbon phosphorus framework (CP_3_), as a promising sulfur host material in Li–S batteries. The electrical conductivity of CP_3_ eliminates sulfur's insulating nature and improves the LiPSs affinity. Manthiram et al. synthesized an intercalation‐conversion hybrid cathode by coupling sulfur with MoTe_2_ nanosheets vertically grown on graphene (MTG) (Figure 7c). MoTe_2_ acts as an intercalation‐type catalyst that both conducts Na^+^ (via sodiation/desodiation) and catalyzes sulfur redox, while graphene boosts conductivity and exposes edge sites. As a result, MTG suppresses the shuttle effect and improves the performance. To evaluate the practical viability of the MTG/S cathode in Na–S batteries, cycling tests were conducted at elevated sulfur loadings of 4.7 and 5.8 mg cm^−2^ at 0.1 C. With a loading of 4.7 mg cm^−2^, the cathode delivered an initial capacity of 1000 mAh g^−1^ with stable cycling over 100 cycles. Remarkably, at a higher loading of 5.8 mg cm^−2^, the MTG/S cathode achieved an initial specific capacity of 887 mAh g^−1^, corresponding to an areal capacity of 5.1 mAh cm^−2^ and maintained a capacity retention of 73.8% after 100 cycles. This superior stability might be due to its wide interlayer spacing (0.69 nm), which accommodates Na^+^ ions without structural degradation during charge and discharge cycles. Further, the vertically aligned MoTe_2_ nanosheets on graphene maintain abundant active edge sites that strongly anchor Na polysulfides, preventing active material loss. Furthermore, MTG/S cathode reduces the accumulation of insulating discharge products that prevent battery degradation [258]. Similarly, Xiaobo Ji prepared 2D MoS_2_ for Na–S cathode (Figure 7d). Their high surface area and exposed edge sites in 2D MoS_2_ accelerate the Na^+^ intercalation. This intercalation expands the interlayer spacing, weakening Mo‐S bonds. As a result, more NaPSs are adsorbed on the catalyst and catalyze their reaction [76]. Beyond layered TMD hosts, scholars utilize various strategies to suppress the shuttle effect [259]. For instance, Chen et al. designed 0D‐2D heterojunction to tackle the shuttle effect in Li–S batteries. The bimetallic selenides on nitrogen‐doped MXene (CoZn‐Se@N‐MX) form double lithiophilic‐sulfifilic binding sites that effectively immobilize and catalytically convert LiPSs intermediates. Furthermore, the hierarchical porous architecture with a large active area in the 0D‐2D heterostructure catalyst enables rapid Li ion diffusion, reduces the activation energy of Li_2_S deposition, and lowers the energy barrier of Li_2_S dissolution. In addition, the assembled CoZn‐Se@N‐MX hybrid synergistically prevents the aggregation of CoZn‐Se nanoparticles and prevents the restacking of the active areas of N‐MX nanosheets during assembly and the LiPSs conversion process. As a result, the synthesized CoZn‐Se@N‐MX hinders the shuttle effect. Further, the cell was investigated at varying sulfur loadings electrodes of 3, 5, and 7.8 mg cm^−2^, with an electrolyte‐to‐sulfur (E/S) ratio of 12, 8, and 5 µL mg^−1^, respectively. Remarkably, even at an ultra‐high sulfur loading of 7.8 mg cm^−2^ with an ultra‐lean E/S ratio of 5 µL mg^−1^ and a low current density of 0.01 C, the CoZn‐Se@N‐MX‐based cathode delivered a high areal capacity of 6.6 mAh cm^−2^ and a reversible capacity of 4 mAh cm^−2^ after 30 cycles at 0.05 C (Figure 8b). The remarkable stability is attributed to the synergistic combination of 0D‐2D heterostructure. This heterostructure prevents aggregation and nanosheet restacking. As a result, CoZn‐Se@N‐MX‐based cathode maintains its active sites and structural integrity during prolonged cycling [260]. Similarly, borophene with different configurations (X_3_, β_12,_ and β‐rhombohedral) has received significant attention. Recently, Liu synthesized β_12_‐Borophene and compared its efficiency with CNT‐doped β_12_‐Borophene in Li–S batteries. Compared with borophene‐doped CNT, β_12_‐Borophene exhibited a maximum capacity with a sulfur loading of 7.8 mg cm^−2^. Computational studies were performed to elucidate the mechanism of the superior areal capacity of β_12_‐borophene. DFT calculations revealed that the polysulfide adsorption energy on β_12_‐borophene ranged from 1.23 to 3.8 eV, far exceeding that of pristine CNT (<1 eV). These studies indicate that β_12_‐borophene exhibits stronger polysulfide anchoring capabilities, effectively inhibiting the shuttle effect. The strong covalent bonds between the borophene layers retard Li^+^ migration and promote in‐plane diffusion. The enhanced in‐plane diffusion accelerates the nucleation and decomposition of Li_2_S_n_ in Li–S batteries. More impressively, the areal capacity of the CNT/Borophene (CNT/B) based cathode reached 5.2 mAh cm^−2^ at a large sulfur loading of 7.8 mg cm^−2^ in a lean electrolyte with an electrolyte‐to‐sulfur (E/S) ratio of 6.8 µL mg^−1^, which is far better than bare CNT‐based Li–S batteries (Figure 8c). The β12‐borophene sheets exhibit remarkable stability owing to their thermodynamically stable β12 phase and strong covalent B‐B bonds, which maintain structural integrity during prolonged cycling [261].

The polysulfide adsorption energy of borophene with graphene, phosophorene, and siloxene. In general, graphene exhibits the weakest polysulfide adsorption among the studied 2D materials (borophene, phosophorene and siloxene), with binding energies of −0.48 −0.57, and −0.74 eV for Li_2_S_6_, Li_2_S_4_ and Li_2_S, respectively. This weak interaction arises from graphene's chemically inert basal plane, characterized by delocalized 𝜋 electrons and the absence of polar functional groups or dangling bonds, so the adsorption mechanism is dominated by weak van der Waals forces rather than chemical bonding. Charge density difference (CDD) analysis supports this result by showing minimal charge redistribution (0.02 e for Li_2_S_6_, 0.83 e for Li_2_S_4,_ and 0.66 e for Li_2_S). Density of states (DOS) calculations confirm graphene retains its semi‐metallic character, with no new states emerging near the Fermi level upon LiPS adsorption, indicating negligible orbital hybridization. In contrast, phosphorene shows substantially stronger and progressively increasing adsorption strength from Li_2_S_6_ (−0.86 eV) to Li_2_S_4_ (−1.07 eV) to Li_2_S (−2.46 eV), driven by exposed phosphorus lone pairs that enable strong chemisorption; its CDD isosurface (0.0015 e Å^−^^3^) and DOS profile reveal semiconducting behavior with clear hybridized states forming at the interface. Borophene demonstrates the strongest chemical trapping among these four materials, with adsorption energies of −1.53, −1.45 and −3.34 eV for Li_2_S_6_, Li_2_S_4_ and Li_2_S, attributable to its electron‐deficient boron atoms that readily accept charge from sulfur species; this is reflected in the largest CDD isosurface value (0.013 e Å^−3^) and pronounced charge transfer (0.77 e, 0.18 e, and −0.90 e), respectively consistent with a metallic system where borophene‐dominated conductivity facilitates rapid electron exchange and continuous polysulfide anchoring [261]. Siloxene, bearing polar Si‐O functional groups, also shows favorable interactions with LiPS species, supported by charge transfer values of 0.22 e, 0.32 e, and 0.49 e for Li_2_S_6_, Li_2_S_4,_ and Li_2_S and a semiconducting DOS signature [83]. Taken together, this trend, graphene <phosphorene <borophene, siloxene, correlates directly with the presence or absence of structural features (P lone pairs, electron‐deficient B atoms, or polar Si‐O bonds) capable of forming strong chemical bonds with LiPSs [262, 263].

Wu et al. utilized computational simulations to determine the optimal adsorption energy of borophene with Fe, Co, and Ni single atoms and FeN_4_, CoN_4,_ and NiN_4_ clusters. Interestingly, integrating a single atom into the borophene structure shifted the d‐band center from left to right and gradually approached the Fermi level, resulting in an improved polysulfide adsorption energy compared to pristine borophene. In contrast, integration of atomic clusters resulted in a more significant shift, where the d‐band center moved closer to the Fermi level. As a result, FeN_4_ had superior sodium polysulfide adsorption owing to anti‐bonding formation [264]. In another example, Ahuja et al. employed density functional theory (DFT) to calculate the efficiency of a germanene monolayer in immobilizing LiPSs (Li_2_S_n_ (n = 1, 2, 4, 6, 8) and Li (S_8_) in Li–S batteries. The DOS revealed that when Li_2_S_n_/S_8_ species were adsorbed on the germanene layer, an additional electronic state was observed near the Fermi level, which resulted from the persistent metallic behavior of the germanene layer. For GeM‐Li_2_S_4_ and GeM‐Li_2_S_8,_ the main impurity bands were Ge(p)‐S(p), which were observed at the Fermi level. The Ge(p)‐S(p) band provides a conducting channel for electrons. In GeM‐Li_2_S and GeM‐S_8_, the metallicity results from the Ge states. Unlike GeM‐Li_2_S, GeM‐Li_2_S_4,_ and GeM‐Li_2_S_8_, very few impurity bands are observed in GeM‐Li_2_S_6. _ Among the six structures, GeM‐Li_2_S_2_ displayed more Ge(p)‐S(p) bands, which were further pushed towards the valence band region, and GeM‐Li_2_S_4_ and GeM‐Li_2_S_8_ provided more channels for electronic conduction. In addition, the partial density of states (PDOS) plot reveals that Ge 4s and 4p orbitals overlap with S‐3p in the energy window of ‐0.4 to −0.1 eV, resulting in strong s‐p and p‐p type hybridization, which is responsible for the Li_2_S_n_ adsorption on the germanene monolayer. Furthermore, increasing the sulfur content indicates that the Ge 4s and 4p orbitals in the valence band maxima of Ge‐Li_2_S are pushed towards the conduction band minimum and generate a conducting channel for Ge‐Li_2_S_8_. This result indicates that the germanene monolayer has suitable adsorption energies, ranging from −1.8 to −0.2.6 eV for Li_2_S_n_ (n = 1, 2, 4, 6, 8) and −2.3 eV (−0.8 eV) for Li (S_8_), respectively, which ensures effective anchoring of LiPSs and prevents the dissolution of polysulfides into the electrolyte. Additionally, nudged elastic band calculations indicate that the germanene monolayer exhibits excellent diffusion properties for Li, S_8,_ and LiPSs, leading to fast charge‐discharge rates, reduced agglomeration, and improved cycle life in Li–S batteries [265]. Similarly, Koratkar et al. synthesized phosphorene and integrated it with carbon nanofiber (CNF) powder. This cathodic electrode decreased polysulfide absorption and enhanced the redox reaction, as demonstrated by the galvanostatic charge and discharge voltage profiles. Furthermore, computational simulations categorized polysulfides into small (Li_2_S and Li_2_S_2_) and large (Li_2_S_3_, Li_2_S_4,_ and Li_2_S_6_) intermediates. Initially, small polysulfide molecules were bonded to phosphorene via S (polysulfide molecules) and P (phosphorene) bonds, along with electrostatic interactions between Li (lithium) and P (phosphorene) atoms. Conversely, large molecules are adsorbed via Li–P electrostatic interactions only. These adsorption energies indicate that polysulfides preferentially adsorb onto the phosphorene catalyst rather than forming large clusters. The adsorbed smaller polysulfides reduced the polarization and increased the redox reaction, along with high sulfur utilization. Owing to this advantage, the assembled cell prevents the dissolution and loss of LiPSs in the electrolyte during discharge cycles [87]. For comparison, the performance of various 2D cathodic matrices was summarized in Table 3. The weak interactions between host materials and polysulfides hinder their conversion in the long run. To address these limitations, 2D materials with defective sites were synthesized. Defective sites improve the material properties and decrease the shuttle effect. For example, catalysts with defective sites improve electron conductivity, thereby influencing polysulfide conversion [266, 267]. Zhongwei Chen's research group has systematically synthesized a 2D defective zeolitic imidazolate framework‐7 (ZIF‐7) with abundant active edges as a sulfur host in Li–S batteries. The synthesized 2D ZIF‐7 with nitrogen defects at the edge sites facilitates polysulfide conversion owing to its improved conductivity (8.24 × 10^−4^ S cm^−1^) and decreased energy barrier for Li_2_S decomposition. Furthermore, owing to its large surface area (16.9 m^2^ g^−1^) and porous structure, the 2D MOF accommodated a sulfur loading of 7.6 mg cm^−2^. Under high sulfur loading (7.6 mg cm^−2^), the S/ZIF‐7 cathode delivered an areal capacity of 4.6 mAh cm^−2^ with an E/S ratio of 5.2 µL mg^−1^ and a higher areal capacity of 5.2 mAh cm^−2^ with an ultra‐lean E/S ratio of 3.8 µL mg^−1^ after 50 cycles at 0.1 C. This exceptional stability might be due to its nitrogen defects that create unsaturated Zn‐N coordination. This unsaturated coordination catalyzes polysulfide, and its porous architecture buffers volume changes during sulfur conversion, while the in situ formed ZnO reinforces structural integrity [268]. Another indispensable case was demonstrated by Thoi et al. In this study, phosphate‐functionalized zirconium metal‐organic frameworks (MOF‐808) were synthesized to enhance the cycling stability of Li–S batteries. Benefiting from the increased ion diffusion and charge transfer kinetics, the MOF‐808‐PO4/S catalyst accelerated the charging time and reduced the discharge time. Furthermore, owing to its high porosity, 3 mg of sulfur (areal loading ≈2.4 mg cm^−2^) was loaded, which exhibited a maximum discharge capacity of 964 at 0.1 C and inhibited the shuttle effect. The exceptional stability might be due to its phosphate groups that anchor Li polysulfides through Zr‐O‐P binding sites. As a result, MOF‐808‐PO4/S catalyst suppresses the shuttle effect, minimizes the active material loss, and improves the cyclic stability [269]. Zhao et al. studied the intactness effect of LiPSs on defective borophene and compared it with the pristine structure. As revealed by ab initio calculations, LiPSs tend to anchor to the defective borophene surface through the Li atom rather than the sulfur atom, owing to differences in electronegativity. It is worth noting that electronegativity decreases in the order: sulfur (2.58) > boron (2.04) > lithium (0.98). The larger electronegativity difference between B‐Li compared with B‐S promotes charge transfer, thereby resulting in higher adsorption energies. Moreover, the borophene structure exhibiting strong VDW forces demonstrated a higher adsorption capacity for LiPSs than the borophene structure with weaker VDW forces. Furthermore, DFT studies revealed that defective borophene also exhibits metallic behavior, similar to pristine borophene, indicating that the presence of defects does not degrade the electrical conductivity in the long run. The inclusion of VDW forces in borophene prevents the LiPSs decomposition and facilitates their conversion into lithium sulfides [270]. Another indispensable case was demonstrated by the Xuan Luo research group. Luo et al. employed DFT calculations to examine the binding strength of different Li_2_S_x_ (x = 4, 6, 8) chain lengths over an antimonene layer with and without oxygen vacancies. DFT results indicate that the O‐doped antimonene layer offers a more active site for anchoring the Li_2_S_x_ species than the pristine surface. Moreover, the optimal adsorption of Li_2_S_x_ on the O‐doped antimonene layer prevents the dissolution of LiPSs in the electrolyte, thereby suppressing the shuttle effect and enhancing the cycling stability. Additionally, charge transfer analysis revealed that the binding of Li_2_S_x_ with oxygen‐impregnated antimonene facilitated electron transfer from the adsorbate (Li_2_S_x_) to the substrate [59]. Wang et al. synthesized a Li_2_S cathode with uniformly dispersed single iron atoms (Fe‐SA) supported on a porous nitrogen‐doped carbon matrix. The Fe‐SA active sites coordinate with Li_2_S molecules and weaken the Li–S bond. DFT calculations reveal that the Li^+^ detachment energy barrier was reduced to 0.81 eV from 3.4 eV. This lowered energy barrier enables much faster Li^+^ transport during the charging process. Further, In situ XANES result reveal that during charging cycle, the Fe valence state was reduced as it interacts with sulfur atom and then shifted back, confirming the regeneration of the active site. These result, confirms that Fe‐SA coordinates with Li2S and weakens the bond. Consequently, this Li_2_S@NC:SAFe cathode delivered superior electrochemical performance of 588 mAh g^−1^ at 12 C [271]. With these carbon, defective, and inorganic catalysts, the shuttle effect can be suppressed more effectively.

**TABLE 3:** Comparison of various 2D cathodic hosts.

Catalyst	Capacity mAh g^−1^ Initial/End	Cycle number/C rate	Capacity retention %	Refs.
RuO*_x_* QDs/GDY	1586/1024.3	200/0.2 C	64.6	[253]
Co‐GDY	1287	0.2 C	—	[81]
Ni@HsGDY/MoS_2_/Ni_3_S_2_	652/486.9	500/2 C	74.7	[254]
MoS_2_/MoSAC/CF	759.5/609.5	100/0.2 A g^−1^	80.3	[76]
MoTe_2_/graphene	1081/901	350/0.1 C	83.3	[258]
CoZn‐Se@N‐MX	1270/1016	100/0.2 C	80	[260]
β_12_‐Borophene/CNT	1110/1000	300/0.5 C	90	[261]
Phosphorene@CNF	1262/600	500/0.2 C	51	[87]
ZIF‐7 600/S	610/520	50/0.1 C	85.2	[268]
MOF‐808‐PO4	964/907	20/0.1 C	84	[269]
Ni@HGDY/Li_2_S	556/516	125/1 C	92	[272]
PCNF/S/BPQD	440/400	200/0.3C	90.9	[273]
c‐FBP‐NC	1224/1015	100/0.2C	82.6	[274]
EBP/EGr	1234/1024	100/0.2 C	82.9	[275]
MoSe_2_/MXene	1221.3/784	500/0.2 C	64	[276]
MoTe_2_	683.5/498	500/1 C	72.8	[277]
TiO_2_/TiS_2_	500/455	100/0.19 C	91	[278]
CoP@BP@C	1119.7/1058	100/0.2 C	94.5	[279]
2D siloxene	462/383	100/0.1 C	82.9	[83]
Ti_4_O_7_/TiN/C	844.6/791.3	450/1 C	93.6	[280]

#### 5.1.2 Kinetics Enhancement Via Active Sites and Electron Mobility

Sluggish kinetic reactions are another critical barrier in MSBs, which directly affect the energy density. The sluggish kinetic reaction is primarily attributed to the inadequate reduction of sulfur to polysulfide conversion, which is driven by a multistep reaction pathway with slow nucleation and low active‑site density. The sluggish kinetic conversion leads to a large voltage hysteresis upon charge and discharge cycles, thereby limiting battery performance. Thus, addressing these challenges requires efficient 2D materials [281, 282, 283]. To lower the overpotential and enhance redox kinetics in Li–S batteries, Yu Chi and colleagues incorporated single nickel (Ni) atoms onto a hydrogen‐substituted graphdiyne support (HGDY). The synergy between the π‐conjugated network of HGDY and the lowered d‐band center of the Ni atom enhanced the electronic conductivity and facilitated ion transport in the Ni@HGDY catalyst. Furthermore, the resulting Ni‐C bond between the HGDY and Ni single atoms regulates the electronic properties of the Ni molecule, thereby enhancing the polysulfide adsorption and conversion. Acetylene linkages and large pores within the HGDY structure effectively hindered the diffusion of polysulfides and improved sulfur confinement. Consequently, the overpotential of the Ni@HGDY catalyst was reduced to 353 mV. Notably, Chi et al. fabricated an anode‐free cell by assembling Cu foil as an anodic electrode and Ni@HGDY/Li_2_S as a cathodic electrode. The assembled device showed a maximum capacity of 664.9 mAh g^−1^ at 0.1 C, whereas the pristine cathodic electrode (Li_2_S) exhibited a capacity of 431.6 mAh g^−1^. After a three‐cycle activation at 0.05 C, the high mass loading (5.5 mg cm^−2)^ cell could maintain over 500 mAh g^−1^ for at least 35 cycles at 0.1 C. The Ni@HGDY catalyst delivers superior stability owing to the strong Ni‐C bonding that embeds Ni single atoms and clusters to the HGDY support. As a result, the catalyst suppresses metal agglomeration and preserves active sites during prolonged cycling [272]. Another example was demonstrated by Lu et al. The sluggish kinetic reaction was hindered by embedding siloxane into the graphene@sulfur catalyst. The synthesized siloxane catalyst exhibited a Kautsky structure with a Si‐O‐Si bridge grafted onto the Si rings. The hydroxyl‐functionalized group exhibited a strong polysulfide anchoring capability, as revealed by DFT calculations. Further, DFT calculations showed that Silicene has a low affinity towards Li–S molecules owing to its weak electrostatic interactions. The highly electronegative oxygen atoms and hydroxyl groups on the siloxene surface regulate ion flux by promoting uniform Li^+^ distribution and lowering the migration energy barrier [186].

**FIGURE 9:**
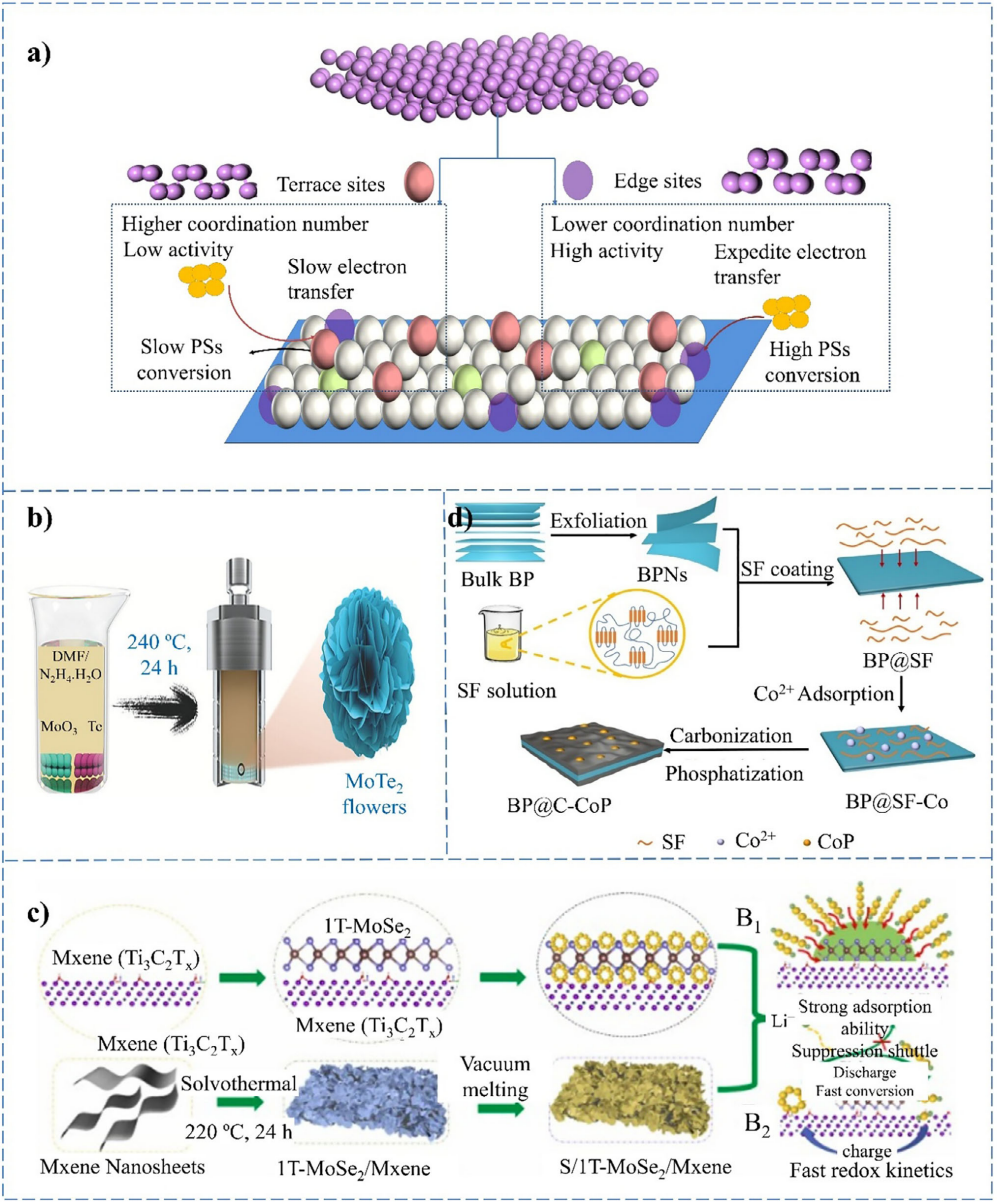
(a) Pictorial representation of edge selective catalyst with highlighted properties, (b) synthesis scheme of MoTe_2_ (reproduced with permission from ref. [277]. Copyright 2024, Wiley); c) Synthesis and adsorption/catalytic conversion mechanism of 1T‐MoSe_2_/MXene representing Adsorption mechanism (B_1_) and catalytic conversion mechanism for the sulfur species (B_2_) (reproduced with permission from ref. [276]. Copyright 2023, Elsevier); and (d) graphical representation of BP@C‐CoP synthesis scheme (reproduced with permission from ref. [279]. Copyright 2024, Wiley).

In general, redox reactions occur at the edge sites of the cathodic surface, and by selectively targeting these edge sites, the catalysts expedite electron transfer and sulfur conversion. Recent studies also demonstrated that edge‐selective catalysts are more favorable for polysulfide confinement than terrace‐site catalysts. Figure 9a displays a pictorial representation of the edge‐selective catalyst with highlighted properties [284, 285, 286]. In this context, Lau et al. successfully synthesized black phosphorus quantum dots (BPQDs) and seamlessly integrated them with sulfur particles on porous carbon nanofibers (PCNFs). Their DFT simulations indicated that decreasing the BPQDs size increased the binding strength of polysulfides. Furthermore, they decisively demonstrated that the binding strength of polysulfides is substantially greater at the edge sites of BPQDs than at the terrace sites. This might be due to the presence of more coordinated edge sites. Meanwhile, the large surface area (1277 m^2^/g) and pore volume (0.82 cm^3^/g) of PCNFs accommodate more sulfur particles and BPQDs. Owing to these advantages, the BPQD/PCNFs/S electrode showed low polysulfide diffusion, improved redox kinetics, and exhibited a higher capacity than those of other edge‐selective catalytic materials, such as tungsten disulfide (WS_2_) and molybdenum disulfide (MoS_2_). The PCNF/S/BPQD electrodes accumulated 8 mg cm^−2^ of sulfur loading and attained an areal capacity of 4.4 mA h cm^−2^ after 200 cycles, outperforming commercial LiCoO_2_ cathodes (4.0 mA h cm^−2^) in Li–S batteries. The electrochemical performance of PCNF/S/BPQD electrodes (Figure 8d) was systematically investigated at varying sulfur areal loadings (4, 6, and 8 mg cm^−2^) at a current rate of 0.1 C. The PCNF/S/BPQD cathode delivered impressive initial specific capacities of approximately 1160, 1020, and 850 mAh g^−1^ for sulfur loadings of 4, 6, and 8 mg cm^−2^, with e/s ratios of 13, 8.7, and 6.5 µL mg^−1^, respectively. After 200 discharge/charge cycles, the reversible capacities remained at approximately 1100, 920, and 760 mAh g^−1^, corresponding to exceptional capacity retentions of ∼95%, ∼90%, and ∼89%, respectively. It is worth noting that the PCNF/S/BPQD electrodes with a high sulfur loading of 8 mg cm^−2^ attained an areal capacity of 4.4 mAh cm^−2^ after 200 cycles, outperforming commercial LiCoO_2_ cathodes (4.0 mAh cm^−2^) in Li–S batteries. This outstanding performance highlights the practical viability of the BPQD/PCNF/S electrode for high‐energy‐density Li–S batteries. The excellent cycling stability under these stringent conditions is attributed to the edge‐selective catalytic properties of the BPQDs, which effectively immobilize and convert Li polysulfides, thereby suppressing the shuttle effect and maintaining structural integrity even under high sulfur loading and lean electrolyte conditions [273].

On the other hand, to improve the redox reaction kinetics, Tang et al. utilized heterointerface/heterojunctions formation in the cathodic host material. Heterojunctions combining conductive materials with polar/nonpolar compounds show high electrical conductivity and active sites that chemically bind polysulfides and increase transport. Tang et al. covalently bonded nitrogen‐doped carbon‐coated multiwalled carbon nanotubes (NC) (denoted as c‐FBP‐NC) with a fully exposed 2D black phosphorus atom. Benefiting from the polarized P‐N covalent bonds, the fabricated cathodic matrix alters the electron distribution between the heterointerfaces, thereby promoting electron transfer from NC to FBP. Additionally, by utilizing the interwoven 1D‐2D structure of the c‐FBP‐NC electrode, FBP stacking was minimized, resulting in a highly porous substrate that facilitated rapid electron and ion transfer. Consequently, LiPSs were chemisorbed, and crossover was limited. The fabricated pouch cell with a sulfur loading of 4 mg cm^−2^ and an electrolyte/sulfur ratio of 3.5 µL mg^−1^ also exhibited a discharge capacity of 983 mAh g^−1^ at 0.1 C with a stable cyclic performance of 70% for 200 cycles. At a sulfur loading of 5.1 mg cm^−2^ with an E/S ratio of 6.4 µL mg^−1^, the c‐FBP‐NC/S cathode delivered a stable areal capacity of 4.01 mAh cm^−2^ after 100 cycles, with a high CE exceeding 98%. Remarkably, even when the sulfur loading was increased to 8.74 mg cm^−2^ with an extremely lean E/S ratio of 5.7 µL mg^−1^, the electrode still achieved a high reversible areal capacity of 7.69 mAh cm^−2^ and maintained 6.37 mAh cm^−2^ after 50 cycles, demonstrating fast reaction kinetics and good electrochemical reversibility. To further validate scalability, a Li–S pouch cell was assembled with a 5.2 cm × 4.4 cm cathode (sulfur loading of 4 mg cm^−2^, double‐sided coating; total sulfur mass of 400 mg) and an ultra‐lean E/S ratio of 3.5 µL mg^−1^. The pouch cell delivered a high specific capacity of 982.8 mAh g^−1^ at 0.1 C and 566 mAh g^−1^ at 1 C, with stable cycling performance. These results confirm that the c‐FBP‐NC catalyst enables excellent sulfur utilization and fast redox kinetics under practically relevant conditions. Further, it is also worth noting that c‐FBP‐NC stability stems from polarized P‐N covalent bonds that regulate electron redistribution by creating electron‐rich FBP planes. As a result, it adsorbs LiPSs convert their intermediate species, preventing shuttle effects during cycling [274]. Similarly, Chou et al. fabricated a graphene/phosphorene heterostructure (EBP/EGr) as a cathodic electrode in Li–S batteries. This 2D/2D heterostructure provides a large interface area to facilitate ionic and electronic migration. Consequently, electrons migrated from EGr to EBP owing to the low Fermi level of the EBP, thereby improving the reaction kinetics. The EBP/EGr@S cathode was tested under high sulfur loadings of 6.1 mg cm^−2^, E/S ratio of 5.7 µL mg^−1^ at 0.2 C. the EBP/EGr@S cathode achieved a high reversible areal capacity of 5.57 mAh cm^−2^ at 0.1 C and maintained 3.21 mAh cm^−2^ at 1 C. More impressively, the cathode delivered an areal capacity of 6.56 mAh cm^−2^ at 0.05 C. The remarkable stability might stem from the electronic interaction between EBP and EGr catalyst. As a result, electrons will migrate from EGr to EBP and form electron‐rich planes that enable strong LiPS adsorption, convert the polysulfide, and prevent shuttle effects during prolonged cycling. Further, this 2D/2D heterostructure confines sulfur species and accommodates volume changes, while EGr prevents EBP degradation, preserving catalytic activity [275].

Although 2D crystalline materials improve the kinetic reaction, their structural collapse in the long run, which hinders their practical feasibility, limits cycling stability and sulfur utilization in real devices [287, 288]. Considering these challenges, researchers have used structure‐dependent catalysts to enhance the reaction kinetics. A recent study demonstrated that morphology‐based catalysts can withstand stress under long run and also, they improve the redox reaction, reduce agglomeration, and accommodate more sulfur [289]. These tailored architectures enhance interfacial contact and facilitate uniform metal deposition, thereby boosting both capacity retention and Coulombic efficiency. For example, Wu et al. synthesized flower‐like MoTe_2_ nanosphere for Na–S cathode (Figure 9b). The assembled MoTe_2_/S cathode showed a robust rate capability of 414 mAh g^−1^ at 2 C. This high capacity is ascribed to the structural advantages of MoTe_2_, which accelerates Na^+^ diffusion and shortens the Na^+^ transport distance. In addition, the abundant active sites of the 2D MoTe_2_ nanosheets and the mixture of mesopores and macropores increase NaPSs adsorption and improve its conversion capability, resulting in higher sulfur utilization. The large interlayer spacing (0.699 nm) of MoTe_2_ and weak VDW interactions on the Te‐Mo‐Te bond allow the easy accommodation of larger ions, which effectively suppresses NaPSs and maintains structural integrity after prolong cycle [277]. Similarly, Lee et al. synthesized an urchin‐like TiO_2_/TiS_2_ heterostructure. In the initial stage of delithiation, Li ions are intercalated into TiS_2,_ which tends to form lithiated TiS_2,_ and sulfur is converted to LiPSs and anchored on TiO_2_. DFT results revealed that TiO_2_ could serve as an adsorbent for polysulfides. TiS_2_ and lithiated TiS_2_ enhanced the sulfur conversion rate. In the discharge cycle, Li ions further intercalate into lithiated TiS_2,_ and LiPSs are reduced to Li_2_S. The kinetics of LiPSs conversion were enhanced owing to the catalytic activity of lithiated S_2._ Consequently, the fabricated cell with the S/TiO_2_/TiS_2_ cathode exhibited a maximum volumetric energy density of 730 Wh/L. Further, S/TiO_2_/TiS_2_ cathode was tested at a high sulfur loading of 7.5 mg cm^−2^ and a E/S ratio of 2.5 µL mg^−1^. Impressively, the reversible areal capacities of the S/TiO_2_/TiS_2_ cell were 6.82, 5.93, 5.36, 4.73, 4.18, and 3.48 mAh cm^−2^ at current densities of 0.5, 1, 2, 3, 4, and 5 mA cm^−2^, respectively. This superior stability arises from the combination of TiO_2_ (strong LiPS adsorption) and TiS_2_ (bidirectional electrocatalysis), which accelerate sulfur conversion during prolonged cycling. It is also worth noting that the intercalated Li^+^ forms lithiated TiS_2_, which further reduces the decomposition barrier of Li_2_S and suppresses shuttle effects. Further, a pouch cell with 6 mg sulfur loading delivered a high capacity of 5.8 mAh cm^−2^ and a capacity retention of 91% after 30 cycles at 0.5 mA cm^−2^ [278]. Wang et al. constructed few‐layer 1T‐MoSe_2_ on MXene surface by one‐step solvothermal reaction. The practical application value of the S/1T‐MoSe_2_/MXene cathode was tested with a high sulfur loading of 6.5 mg cm^−2^ at 0.1 C. The cathode presented a high initial areal capacity of 6.9 mAh cm^−2^ with a capacity retention of 73.1% after 200 cycles. Simultaneously, a favorable areal capacity of 4.9 mAh cm^−2^ was maintained after 200 cycles. This might be due to the metal‐like conductivity of octahedrally coordinated 1T‐MoSe_2._ Further, it exposes active edges/defects. These active sites lower the Li_2_S nucleation and oxidation barriers (Figure 9c). In addition, the intimate face‐to‐face contact between adjacent nanosheets suppresses polysulfide shuttling more effectively and improves sulfur redox reversibility. Meanwhile, the open 2D porous pathways facilitate faster electrolyte penetration and shorten ion transport pathways, leading to higher rate capability and better cycling stability in Li–S batteries. Furthermore, polar terminal groups (─O/─OH/─F) on 2D Ti_3_C_2_T_x_ layers chemically adsorb LiPSs and catalyze their conversion [276]. Similarly, Xu et al. synthesized ultrafine cobalt phosphide (CoP) nanoparticles and uniformly distributed them over the nitrogen‐doped carbon shells of 2D black phosphorus (BP) nanosheets (Figure 9d). The electrostatic repulsion between the BP nanosheets prevented the agglomeration of silk fibroin (SF) (carbon source) and CoP. Consequently, CoP with an average size of 2.33 nm was uniformly distributed over BP@C. Furthermore, the transition of amorphous carbon via sp^2^ in SF increased the decomposition temperature of BP from 380°C to ∼ 450°C. In addition, CoP is expected to provide more active catalytic sites and adsorb a wide range of sulfur species. As expected, CoP@BP@C sublimated 80.3% of sulfur, accelerated the redox kinetics, and improved the ion/electron transport capability [279]. Similar to Tang et al. [274] and Chou et al. [275], Young‐Si Jun and co‐workers synthesized siloxane and incorporated it into different carbon‐based substrates. Interestingly, owing to the multiple effects of siloxane, the fabricated Li–S battery demonstrated an areal capacity of 7.49 mAh cm^−2^ and a high volumetric energy density of 612 Wh/L under 10 mg S cm^−2^ sulfur loading. This exceptionally high energy density is attributed to the abundance of Lewis acid sites in siloxene. Furthermore, the hydroxyl groups of siloxene are aligned perpendicular to the silicon skeleton, enabling linear alignment of LiPSs molecules in their interlayers. Additionally, the siloxene hydroxyloup form a strong polysulfide‐philic surface, which improves the wettability of organic electrolytes and also increases their mechanical strength. Siloxene reacts with polysulfide and generates insoluble thiosulfate, which further strengthens polysulfides by revealing a fresh Si surface, minimizing the sulfur loss [83].

Among the above‐discussed 2D materials, we have quantitatively compared the performance of emerging 2D materials in Li–S batteries with traditional material. As shown in Table 4, emerging 2D host shown higher potential in retaining performance. In addition we have also compared single 2D material (GDY) in both Li and Na–S system [247, 286]. Compared with Li–S battery, Na–S cell shows higher initial capacity (1250 mAh g^−1^), which can be attributed to its much higher sulfur content (53.6% vs. 30.2%). However, the Li–S cell demonstrates superior stability, retaining 88.3% of its capacity after 100 cycles compared to only 64% for the Na–S cell. This stark difference in cycling stability is the most critical point and highlights the fundamental limitation of a single 2D material across different alkali metal systems. The poor stability in the Na–S system is primarily due to the severe “shuttle effect” of sodium polysulfides. The larger sodium ions (Na^+^: 0.204 nm vs. Li^+^: 0.152 nm) interact differently with the sulfur cathode during cycling, making the intermediate polysulfide species more soluble in the electrolyte. This causes them to migrate away from the cathode, leading to a rapid loss of active material and steep capacity decline. Furthermore, the larger ionic radius of Na^+^ causes more significant volume expansion and structural strain during cycling, which accelerates cathode degradation. The deposition of sodium sulfide (Na2S_x_) during discharge is characterized by a high interfacial energy barrier and the formation of small, inhomogeneous particles, which limits reaction kinetics. To overcome these limitations, SAC doping and heteroatom doping have proven to be effective approaches.

**TABLE 4:** Comparison of emerging 2D host with tradition material at 0.5C.

Material	Capacity mAh g^−1^ (Initial/End)	Cycle No/C rate	Refs.
Graphene	1000/886	1000/0.5	[290]
2D Py‐COF	929/633	200/0.5	[80]
2D MoS_2_	1271.8/676.1	73/0.5	[291]
GDY	850/850	800/0.5	[292]
Phosphorene	1092	0.5	[87]
Borophene	1110/1000	300/0.5	[261]
Siloxene	749/539	200/0.5	[83]

### 5.2 Anode Protection and Dendrite Inhibition

Despite the appealing properties of metallic anodes, their practical application is severely hindered by dendritic growth, low CE, and poor cycling stability. These issues originate from the heterogeneous nucleation and uneven deposition of alkali metals (Na or Li) on the anode surface during plating and stripping. To address these challenges, various anode protection strategies have been explored, including artificial solid‐electrolyte interphase (SEI) layers and electrolyte additives. For instance, Duan et al. synthesized nitrogen‐doped 3D porous carbon nanofibers embedded with zinc nanoparticles (3D ZNNCS) to guide uniform Li nucleation. The resulting 3D ZNNCS reduced the local current density. Furthermore, the abundant lithiophilic sites with low nucleation barriers exhibited a low overpotential of 20 mV at 3 mA cm^−2^ and enabled a stable Li plating/stripping process for 3200 h in symmetric cells [273, 293]. Ping et al. developed a polyvinyl alcohol (PVA) layer on a Li host. The abundant hydroxyl (‐OH) polar groups in the PVA layer showed strong binding energy with Li atoms and effectively regulated Li plating/stripping behaviors. Consequently, the fabricated cell displayed superior long‐term stability for over 1000 h with a low average voltage hysteresis of 17 mV at 1 mA cm^−2^ [294]. Similarly, fluorinated coatings and pre‐patterned or porous surfaces can promote uniform nucleation, redistribute ion flux, reduce local current density, and suppress dendrite growth [266, 267, 268]. Although studies demonstrate that such protective layers enable stable cycling over hundreds of cycles, they often fall short in long‐term performance or fail under aggressive cycling conditions. To overcome these drawbacks, 2D materials have emerged as promising candidates.

#### 5.2.1 Artificial SEI Layers

**FIGURE 10:**
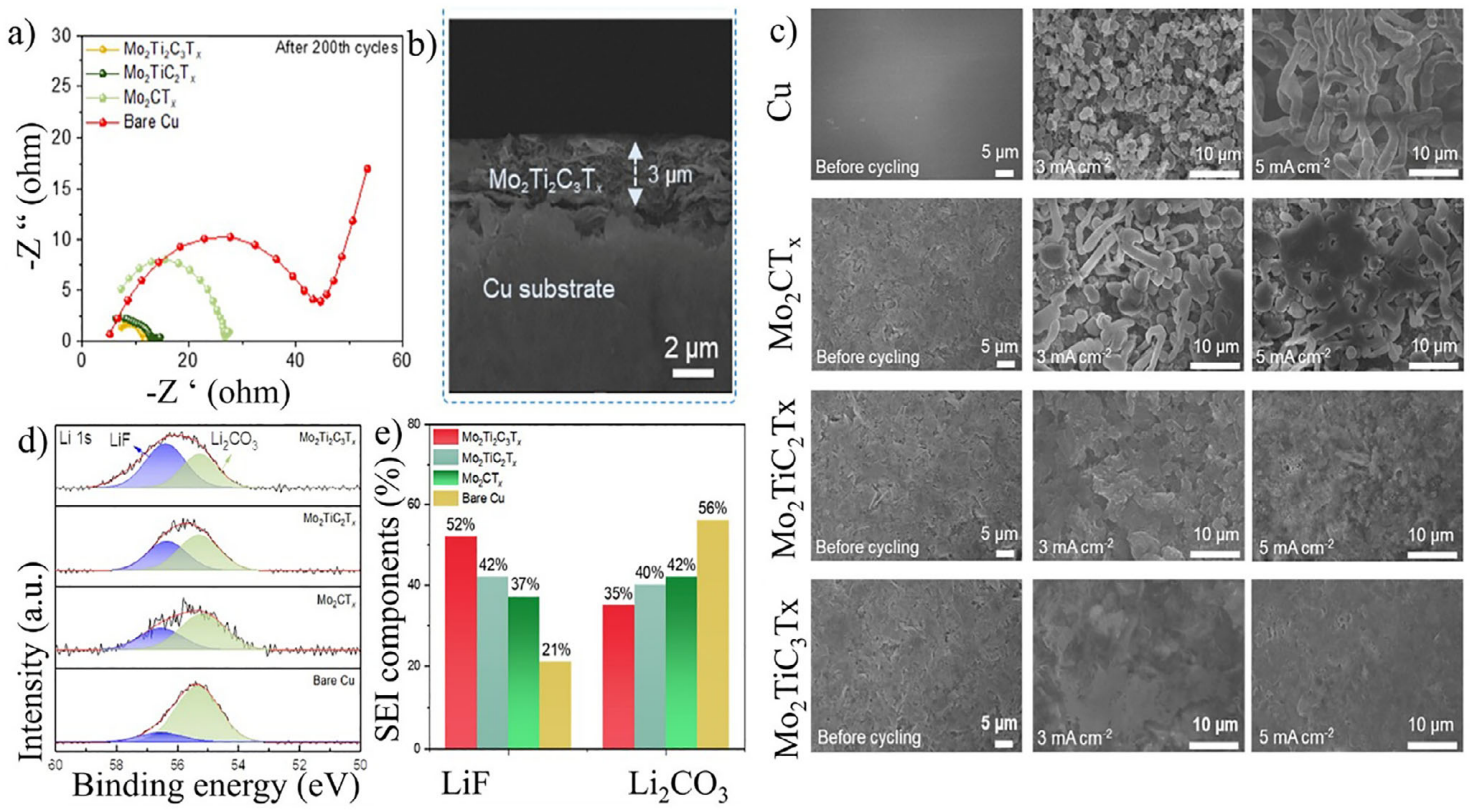
(a) EIS spectra of bare Cu, Mo_2_CT_x_ Mo_2_TiC_2_T_x,_ and Mo_2_Ti_2_C_3_T_x,_ (b) SEM image showing Mo_2_Ti_2_C_3_T_x_ thickness on Cu substrate, (c) SEM images (d) XPS; and (e) SEI composition of bare Cu, Mo_2_CT_x_ Mo_2_TiC_2_T_x,_ and Mo_2_Ti_2_C_3_T_x_ (reproduced with permission from ref. [303]. Copyright 2025, Elsevier).

Artificial SEI layers induced by 2D materials have shown tremendous potential in anode protection beyond the natural SEI layer. Artificial SEI layers form a protective barrier between the alkali metal and the electrolyte. This reduces unwanted reactions, stabilizes the interface, and deposits uniform metal [295, 296]. To suppress dendritic growth, an artificial SEI layer should have the shear modulus (G) at least twice that of the underlying metal anode (4.2 GPa for Li and 3.3 GPa for Na) [297, 298]. For instance, High shear moduli of 2D materials such as MXenes and TMDCs enable effective mechanical reinforcement for metallic anodes by preventing SEI fracture through the alleviation of stress concentrations. The robust 2D layers act as a flexible yet stiff scaffold that accommodates volume changes during cycling while suppressing crack propagation in the solid‐electrolyte interphase [299, 300]. 2D materials with high ductility further enhance the SEI resilience by accommodating volumetric expansion and contraction during cycling. Further, 2D materials such as MXenes and TMDCs demonstrate strain tolerances, allowing them to flex elastically without cracking, preventing continuous SEI fragmentation and associated parasitic reactions. The chemical stability of the artificial SEI is paramount, given its proximity to highly reactive metal anodes and polysulfide intermediates [296]. 2D materials, such as g‐C_3_N_4_ and fluorinated graphene, are chemically inert and thermally stable. Similarly, the robust TM‐X covalent bonds (bond energy ∼200‐400 kJ/mol) underpin TMDCs exceptional thermal stability (>800°C) and prevent the SEI layer from degrading [105, 145, 301]. For instance, Wang et al. chemically exfoliated MoS_2_ nanosheets (EMoS_2_) and used them as an artificial SEI on Li‐anode (EMoS_2_@Li). Furthermore, the ample sulfur vacancies on EMoS_2_ provide lithiophilic sites, and the 1T crystal boosts interfacial conductivity and acts as a lithiophilic interfacial ion‐transport skin to reduce the Li nucleation overpotential and regulate Li^+^ flux. Further, EMoS_2_@Li Young's modulus and its continuous layered structure, the proposed EMoS_2_@Li suppresses the growth of Li dendrites and repeat breaking/reforming of the SEI [302].  Similarly, Zaman et al., showed that among three Mo‐based MXenes modified Li anode, bimetallic Mo_2_Ti_2_C_3_T_x_ forms the most fluorine‐rich, LiF‐dominant SEI layer, enabling uniform Li deposition and suppressing dendrite growth. It is also worth noting after 200 cycles, EIS spectra (Figure 10a) of Mo_2_TiC_2_Tx had the lowest charge‐transfer resistance (∼11.5 Ω) compared with Mo_2_TiC_2_Tx (13 Ω), Mo_2_CT_x_ (26 Ω) and bare Cu (44 Ω) confirming its most stable interface. Further, Figure 10b,c SEM images reveal that after cycling Mo_2_Ti_2_C_3_T_x_ maintained a smooth, dendrite‐free surface at 3–5 mA/cm^2^, while Cu and Mo_2_CT_x_ showed severe dendritic/mossy growth. Lastly, SEI composition was quantified by deconvoluting the Li 1s XPS peaks into LiF (∼56.4 eV) and Li_2_CO_3_ (∼55.3 eV) components (Figure 10d,e), with peak‐area percentages giving Mo_2_Ti_2_C_3_T_x_ the highest LiF content (52%) versus 21% for bare Cu, directly linking its high surface ‐F termination to superior SEI stability. As a result, the Mo2TiC2Tx‐based device showed 99.79% CE at 3 mA cm^−2^ even after 544 cycles and 70% capacity retention after 100 cycles at 10 mg cm^−2^ cathode loading. This superior stability stems from its abundant fluorine terminations, which induce the formation of a LiF‐rich SEI layer. This stable inorganic SEI displays high ionic conductivity, low diffusion barrier, and robust mechanical strength, enabling uniform Li deposition without dendrite growth. As a result, they suppress parasitic reactions and maintains electrode integrity during prolonged cycling [303]. Guo et al. synthesized a conductive, ultrathin 2D COF that acts as an artificial SEI, delivering long‐life, low‐polarization Li plating/stripping at high current and capacity. The π‐conjugated 2D lattice improves the interfacial conductivity, which reduces the interface impedance and facilitates the uniform distribution of Li^+^ flux. Further, the porous 2D COF sheet guides Li^+^ uniformly (high apparent t_l__i_^+^), resists fracture, and deposits Li densely and smoothly rather than forming dendrites (Figure 12a) [304]. Hu et al. build an in situ interlayer on sodium metal by reacting Na with g‐C_3_N_4_. This reaction yields a nitrogen‐rich, mixed ionic‐electronic conducting skin on Na‐an ionic‐electronic dual‐conducting interlayer. These N‐rich sites in the interlayer are highly sodiophilic, lowering the Na nucleation barrier and homogenizing the Na^+^ flux at the metal surface. This ionic‐electronic dual‐conducting interlayer provides continuous paths for both electrons and Na^+^, reducing interfacial resistance and suppressing dendrite growth/SEI cracking during plating‐stripping (Figure 12b) [305]. As a result, artificial SEI layers induced by 2D materials suppress dendrite growth and cracks on the anode surface. Table 5 compares the electrochemical performance of various 2D modified anodes.

**TABLE 5:** Comparison of electrochemical performance of the 2D modified Na anode.

Materials	Current density mA cm^−2^	Capacity mAh cm^−2^	Cycle number	Refs.
Na_15_Sn_4_/ Cu_6_Sn_5_	0.5	1.0	110	[309]
Mxene/Na_3_Ti_5_O_12_ Nanowires	5	20	300	[310]
Ti_3_C_2_T_x_‐CC	1	5	400	[311]
CT‐Sn(II)@Ti_3_C_2_	3	1	200	[312]
Cu@Sn‐NPs	2	1	2000	[313]
Cu@Sb‐MPs	2	1	1700	[313]
Cu‐Cu@C	1	1	600	[314]

#### 5.2.2 Mechanical Buffering by 2D Composite

**FIGURE 11:**
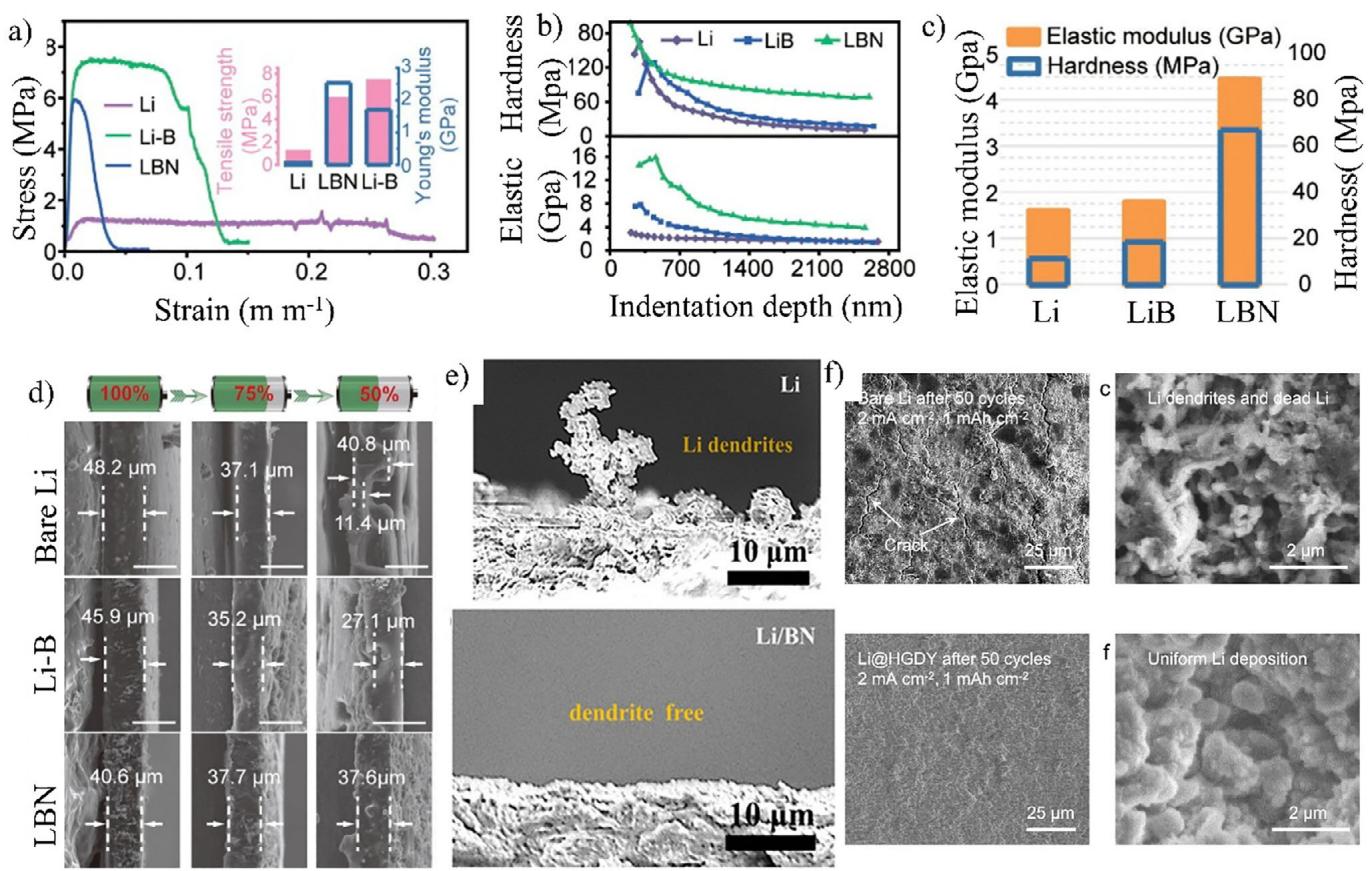
(a) Stress–strain curves (b, c) nanoindentation measurements including elastic modulus and hardness (d) Cross‐sectional SEM images of Li, Li‐B and LBN after stripping 0%, 25% and 50% of dendrite free images (reproduced with permission from ref. [306]. Copyright 2024, Elsevier); (e) SEM images of Li and Li/BN at 1.0 mA cm^−2^. (Reproduced with permission from ref. [308]. Copyright 2021, American Chemical Society; and (f) Low‐magnification and high‐magnification SEM images of pure Li electrodes after 50 cycles of stripping/plating in carbonate electrolytes at the cycling condition of 2 mA/cm^2^, 1 mAh/cm^2^compared with Low‐magnification and high‐magnification SEM images of the Li@HGDY electrode after 50 cycles of stripping/plating in carbonate electrolytes at 2 mA/cm^2^, 1 mAh/cm^2^ (reproduced with permission from ref. [307]. Copyright 2024, American Chemical Society).

Significant volume changes during Li and Na plating/stripping (BCC lattice parameters ≈ 4.29 Å for Na and 3.51 Å for Li) cause interfacial cracking, SEI fracture, and uneven stress distribution, triggering localized nucleation, dendrite growth, and rapid capacity decay in AMBs [15]. To alleviate this mechanical instability, 2D composite hosts with high elastic and tensile strength frameworks buffer volume expansion while homogenizing ion flux. In these designs, robust 2D materials (graphene, MXenes, phosphorene and 2D carbons) are combined with porous scaffolds to form mechanically integrated networks: the 2D component provides high Young's modulus, shear strength and continuous conduction pathways, while the porous phase offers internal voids and lithiophilic/sodiophilic sites to accommodate plated metal without pulverization [281, 282, 283, 284, 285, 286, 287]. For instance, chen et al., doped 2D BN and BN fiber with Li anode (Li, Li–B, and LBN) and compared stress–strain curves. As shown in Figure 11a, Li‐B and LBN displayed tensile strengths of 7.47, 5.9 MPa more than quadruple of bare Li (1.27 MPa). Whereas LBN's Young's modulus (2534 MPa) far exceeding Li‐B's (1705 MPa). Figure 11b,c presents nanoindentation‐derived elastic modulus and hardness, showing LBN achieves superior values (3.37 MPa hardness, 4.5 GPa modulus) compared to Li‐B's near‐Li‐like properties (0.95 MPa, 1.8 GPa). These results indicate that 2D‐modified Li anodes increase the mechanical properties and suppress the dendrite formation. Further, Figure 11d displays cross‐sectional SEM images of Li, Li–B, and LBN electrodes after stripping 0%, 25%, and 50% of free Li, demonstrating that LBN retains over 85% of its initial thickness. In contrast, bare Li and Li‐B show severe local thickness variations (up to 29.4 µm and 40.9%), respectively. As a result, LBN anode shown superior stability, exceeding 2500 h in symmetric cells, with a high average CE of 99.69%. The remarkable stability stems from its dual‐phase architecture (LiB/Li_3_BN_2_). Initially, Li_3_BN_2_ displayed high ionic conductivity (2.85 mS cm^−1^) and strong Li affinity, whereas LiB fiber displayed high electronic conductivity and lithiophilicity. This synergistic combination accelerates the Li^+^ diffusion and induces uniform dendrite‐free Li deposition even under high current densities [306]. Similarly, BN‐modified Li (Figure 11e) and HGDY‐modified Li (Figure 11f) anode reduced dendrite formation [307, 308].

Recently, Li et al. engineered a 1D/2D Na_3_Ti_5_O_12_/MXene hybrid host by growing Na_3_Ti_5_O_12_ nanowires between Ti_3_C_2_T_x_ MXene nanosheets, creating a layered composite that enhances Na^+^ diffusion and provides abundant sodiophilic sites. In this architecture, Na preferentially deposits within the interlayer voids, allowing the host to accommodate large Na inventories and regulate nucleation, enabling dendrite‑free Na plating at current densities up to 10 mA cm^−2^. To validate its practical impact, Li et al. assembled full cells using Na_3_Ti_5_O_12_/MXene/Na anodes paired with both C/S and Na_3_V_2_(PO_4_)_3_ (NVP) cathodes. With a C/S cathode in 1 M NaPF_6_/diglyme electrolyte, the Na_3_Ti_5_O_12_/MXene/Na∥C/S cell delivers a high reversible capacity of 456 mAh g^−1^ and retains 76% of its capacity after 500 cycles at 0.5 C. Further, the hybrid host improves Na plating/stripping kinetics and mitigates the shuttle effect. A similar trend is observed in Na‐NVP full cells using 1 M NaClO_4_ in 1:1 (v/v) EC/PC: compared with NVP/bare Na cells (66 mAh g^−1^ after 140 cycles, 96.1% CE), the NVP Na_3_Ti_5_O_12_/MXene /Na configuration maintains 80 mAh g^−1^ after 140 cycles at 1 C with an average CE of 97.3%. The remarkable stability stems from its unique 1D/2D Na_3_Ti_5_O_12_/MXene matrix, which facilitates the formation of a stable SEI on the Na surface and suppresses dendrite growth and dead‑Na formation. By contrast, bare Na anodes suffer from unstable SEI, electrolyte consumption, and Na loss due to the highly reactive, dendritic Na surface, leading to inferior cell performance [310].

**FIGURE 12:**
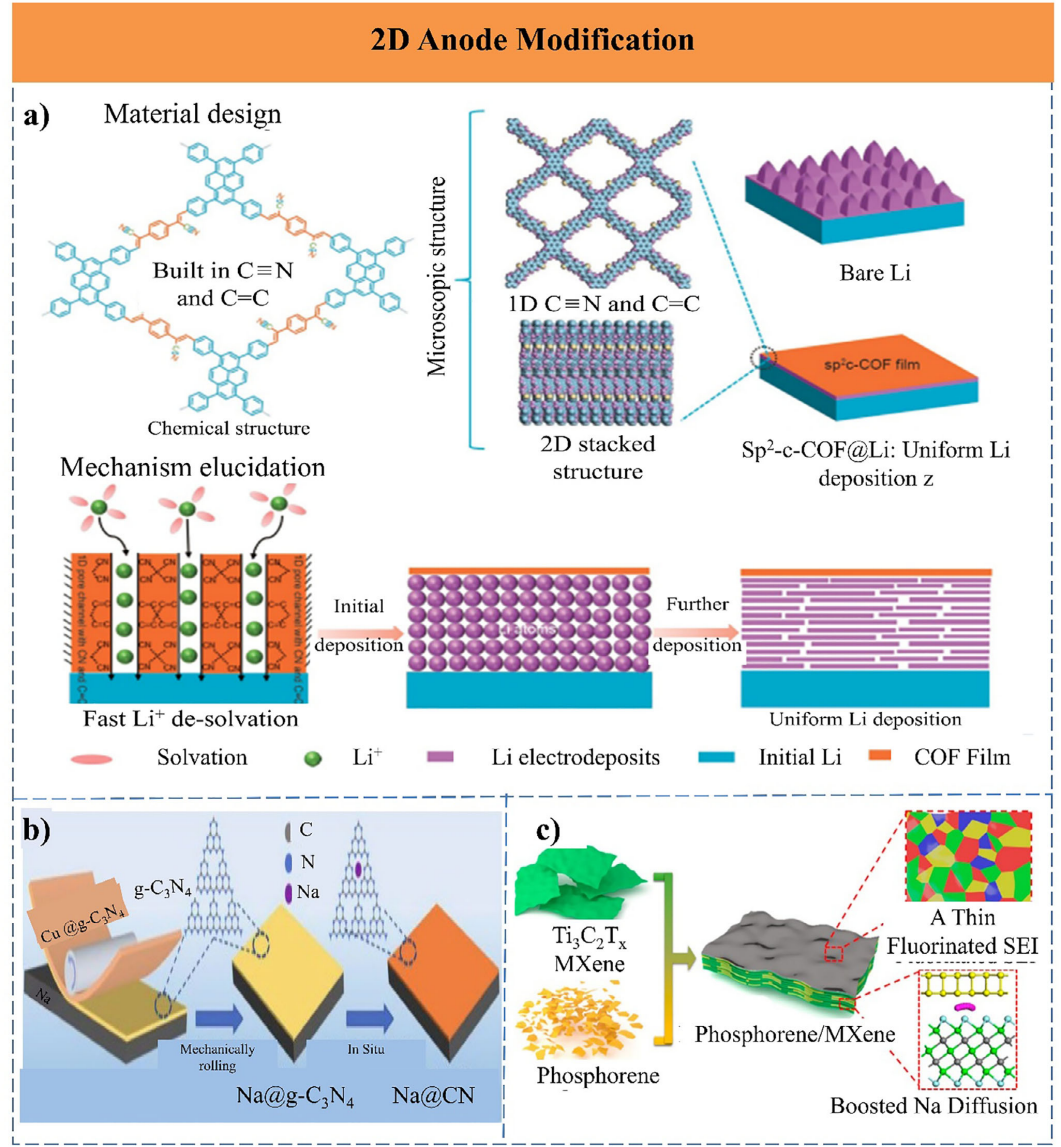
(a) structural design and mechanism of sp^2^‐COF‐modified Li anodes for uniform Li deposition (reproduced with permission from ref. [304]. Copyright 2023, Springer Nature); (b) schematic illustration on fabrication process of Na@g‐C_3_N_4_ and Na@CN composites (reproduced with permission from ref. [305]. Copyright 2023, Elsevier); and (c) schematic illustration of a phosphorene/MXene heterostructure that has a thin fluorinated SEI layer, boosting Na diffusion (reproduced with permission from ref. [315]. Copyright 2020, American Chemical Society).

On the other hand, Phosphorene, with its shorter ion diffusion pathways, high surface‑to‑volume ratio, and dense active sites, has also attracted considerable attention for anode protection and interfacial stability (Figure 12c). However, phosphorene suffers from severe restacking during electrode fabrication and large volume changes during alloying/de‑alloying, which undermine structural integrity and rapidly deteriorate battery performance. To overcome these drawbacks, Wang et al. stacked phosphorene with Ti_3_C_2_T_x_ MXene. In this hybrid, F‑terminated MXene surface groups spontaneously form an F‑rich (NaF‑dominated) interphase on the anode during cycling, which stabilizes the SEI and enables fast, uniform Na^+^ transport. The fluorinated interphase suppresses side reactions, lowers nucleation barriers, and buffers phosphorene volume changes, thereby improving CE and cycle life in Na‑ion cells [309]. Similarly, Cao et al. constructed a Ti_3_C_2_T_x_ ‑modified carbon cloth (Ti_3_C_2_T_x_‑CC) as a flexible host for Na metal. The sodiophilic Ti_3_C_2_T_x_ coating provides abundant, uniformly distributed nucleation sites and a continuous, highly conductive surface, promoting homogeneous Na^+^ flux and planar, laterally oriented Na deposition; Na thus grows as a smooth, dense layer rather than mossy dendrites, significantly reducing local stress concentration and interfacial cracking in both ether‐ and carbonate‑based electrolytes. To demonstrate practicality, Na‐Ti_3_C_2_T_x_‐CC was paired with an NVP cathode to assemble Na‐Ti_3_C_2_T_x_ ‑CC∥NVP full cells. Compared with conventional Na∥NVP cells, Ti_3_C_2_T_x_‐CC‑based full cells deliver higher capacities and lower polarization at high current densities, owing to the stabilized Na metal interface and improved electron/ion transport through the Ti_3_C_2_T_x_‐CC skeleton. A flexible full cell using an NVP cathode and a Na‐Ti_3_C_2_T_x_‐CC anode achieved an areal capacity of 15 mAh cm^−2^. These flexible Na‐Ti_3_C_2_T_x_‐CC∥NVP batteries exhibit stable charge‐discharge plateaus with low polarization over 2.0–4.3 V and show excellent rate performance with average CE of ∼98%. Under bending at 0°, 90°, and 270° (500 mA g^−1^), the flexible cells retain ∼88%, 88%, and 89% of their initial capacity after 300 cycles, respectively, confirming that the MXene‑modified carbon cloth architecture can mechanically buffer Na volume changes while preserving electrochemical performance in flexible Na metal full cells [311]

Li et al. further demonstrated a CT‑Sn(II)@Ti_3_C_2_ electrode, comparing its performance with Cu foil, Ti_3_C_2,_ and CT‑Ti_3_C_2_ hosts. Sn^2+^ dopants confined between Ti_3_C_2_ layers enhance Na nucleation and generate Na–Sn alloy seeds within the interlayers; by guiding initial Na nucleation and confining growth to the Ti_3_C_2_ galleries, the CT‑Sn(II)@ Ti_3_C_2_ host minimizes hot spots and favorable sites for dendrite formation, thereby delivering remarkable stability and high Coulombic efficiency under elevated current densities and high capacities. Full cells were fabricated using CT‑Sn(II)@Ti_3_C_2_/Na and bare Na anodes with NVP cathodes in 1 M NaClO_4_/EC‐DEC (1:1 v/v). Compared with NVP/bare Na, the NVP/CT‑Sn(II)@Ti_3_C_2_/Na full cell exhibits superior performance, maintained a reversible capacity of 95 mAh g^−1^ at 1 C over 200 cycles. In contrast, NVP/bare Na cells show similar discharge capacities (∼92 mAh g^−1^ at 1 C) with CE below 50%, due to nonuniform Na deposition. This superior stability stems from the intercalated Sn^2+^ species between Ti_3_C_2_ layers. During plating, Sn^2+^ first reacts with Na to form Na–Sn alloy seeds, which guide uniform Na deposition and accommodate deposited Na due to its wide interlayer spacing 2.5 nm. Consequently, CT‑Sn(II)@Ti_3_C_2_/Na anode maintains its structural integrity throughout prolonged cycling [312]. Liu et al. and co‑workers designed hybrid 2D phosphorene/Ti_3_C_2_T_x_ MXene architectures that exploit complementary planar conduction pathways and intimate interfacial contact to boost electronic and Na^+^ transport; these hybrid alloy hosts stabilize the 2D heterostructure and mitigate volume expansion during cycling in Na metal anodes. Wang et al. constructed a protective layer comprising 2D Na_15_Sn_4_/Cu_6_Sn_5_ alloys on Cu foil via electrodeposition and sodiation. The Na_15_Sn_4_/Cu_6_Sn_5_‑modified Cu electrode reduces electrolyte consumption during Na plating/stripping, and asymmetric Na∥Na_15_Sn_4_/Cu_6_Sn_5_/Cu cells with 100/200 µL electrolyte exhibit stable cycling for 110/180 cycles at 0.5 mA cm^−2^ and 1 mAh cm^−2,^ with an average CE of 94.9%. In Na metal full cells, Na_15_Sn_4_/Cu_6_Sn_5_/Cu electrode enable highly efficient and long‑term operation: a Na_3_V_2_(PO_4_)_3_∥Na_15_Sn_4_/Cu_6_Sn_5_/Cu anode preloaded with 3 mAh cm^−2^ retains 96% of its capacity after 200 cycles at 1 C and maintains an average CE above 99.9%, directly evidencing that the two‑alloy‑layer anode can sustain practical Na metal operation with minimal electrolyte consumption. This exceptional stability might be due to its Na15Sn4 surface that promotes dense 2D Na growth and provides a stable SEI layer during sodiation that effectively suppresses continuous electrolyte decomposition. This dual‐alloy design limits the exposure of Cu surfaces, reducing electrolyte consumption and maintaining stable electrode/electrolyte interfaces throughout prolonged cycling [290]. Overall, integration of 2D materials provides a multifaceted approach to stabilize alkali metal anodes. These strategies effectively suppress dendrite growth, accommodate volume expansion, and enhance interfacial stability, thereby paving the way for long‐term AMBs.

### 5.3 Separator Modifications

**FIGURE 13:**
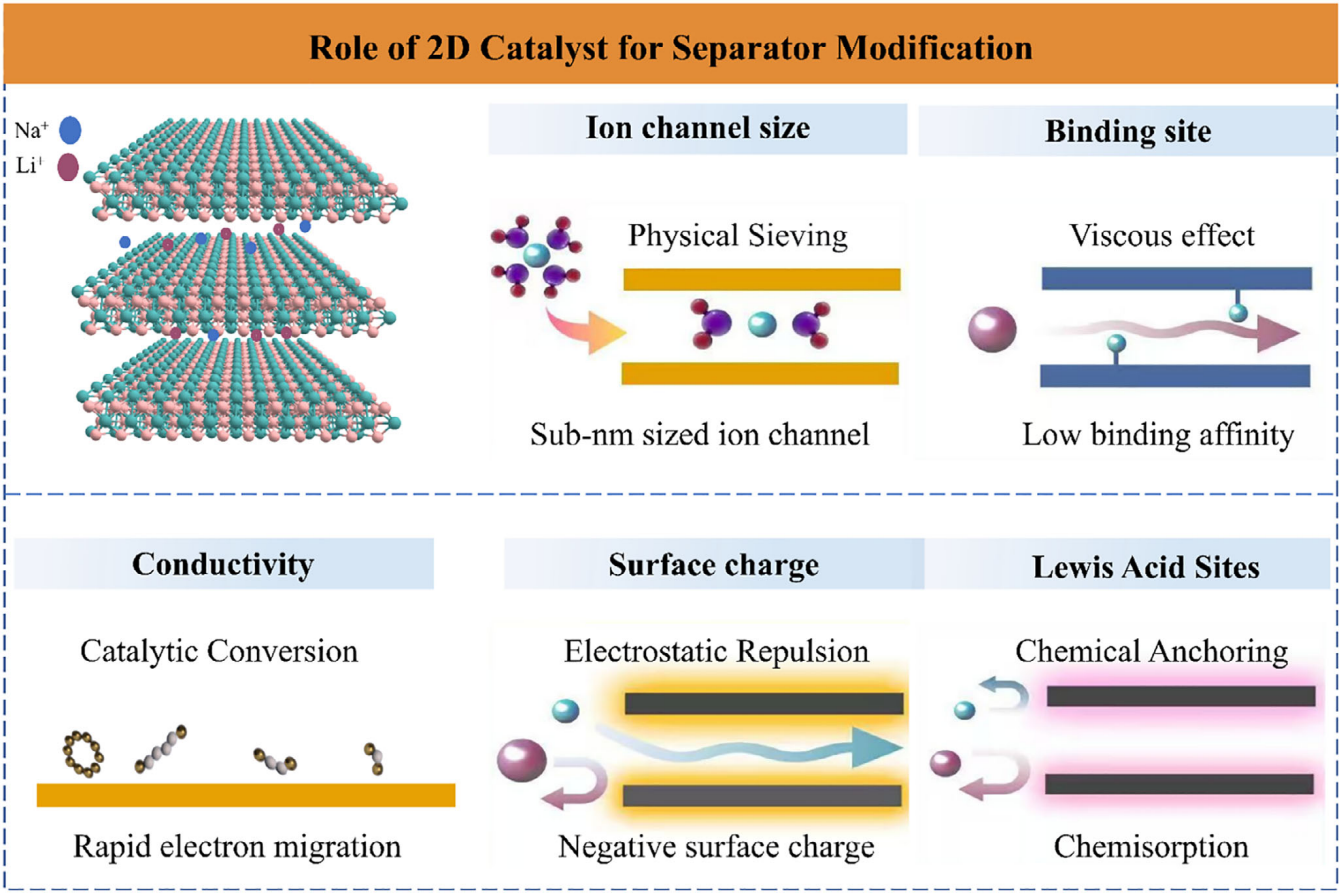
Role of 2D MBene‐modified separators in Li–S/Na–S cells. A lamellar MBene coating tailors ion transport and block polysulfide at the separator by: (i) providing sub‐nanometre ion channels for physical sieving‐Li^+^/Na^+^ pass while long‐chain polysulfides are blocked; (ii) offering low‐affinity binding sites/viscous confinement that slow crossover without immobilizing cations; (iii) conductivity that helps to catalyze the reaction; (iv)imparting negative surface charge that electrostatically repels anionic polysulfides and homogenizes ion flux; and (v) exposing Lewis‐acidic metal sites that chemically anchor (chemisorb) polysulfides near the coating. Together these effects suppress shuttle and dendrite growth and improve cycle stability.

The separator is a key component of MSBs because it must enable fast transport of metal ions between the cathode and anode while simultaneously suppressing polysulfide crossover. In high‐energy‐density configurations, this selectivity becomes even more critical: if polysulfides migrate through the separator, they trigger severe shuttle reactions that accelerate electrolyte consumption, reduce CE and discharge capacity, and promote non‐uniform metal deposition that evolves into dendritic growth. Conventional separators such as polyolefin films (PF) and glass‐fiber (GF) membranes are unable to block polysulfide penetration, resulting in low energy density [316, 317]. Although increasing separator thickness can partially impede polysulfide diffusion, it lowers gravimetric/volumetric energy density and can increase short‐circuit risk. On the other hand, separators also exhibit thermal and mechanical drawbacks: PF can undergo thermal shrinkage/softening at elevated temperatures, causing pore collapse and loss of dimensional integrity, and their limited mechanical robustness makes them more vulnerable to deformation. To overcome these coupled issues, numerous efforts have focused on structuring separators using 2D materials. As highlighted in Figure 13, 2D separators can regulate ion transport and polysulfide confinement through multiple, cooperative mechanisms. First, their sub‐nanometer interlayer creates well‐defined ion channels that provide physical sieving: small metal ions can pass, whereas larger solvated species and polysulfides are hindered; however, excessively narrow channels can raise ionic resistance, so channel size must be optimized to retain conductivity. Second, the surface charge of the 2D layers introduces electrostatic selectivity. In particular, a negatively charged surface can repel anionic polysulfides, acting as an electrostatic gate that suppresses crossover even when size exclusion alone is insufficient. Third, weak and reversible binding sites on the 2D framework can impose a viscous/drag effect, slowing polysulfide diffusion without irreversibly trapping active species, which helps mitigate shuttling while preserving reaction reversibility. Finally, Lewis‐acidic sites provide stronger chemical anchoring, up to chemisorption, enabling robust adsorption of electron‐rich polysulfide species near the cathode side. Furthermore, 2D materials increase the thermal and mechanical stability and structural integrity during repeated charge‐discharge cycles.

#### 5.3.1 2D Material Coatings to Block Polysulfides

**FIGURE 14:**
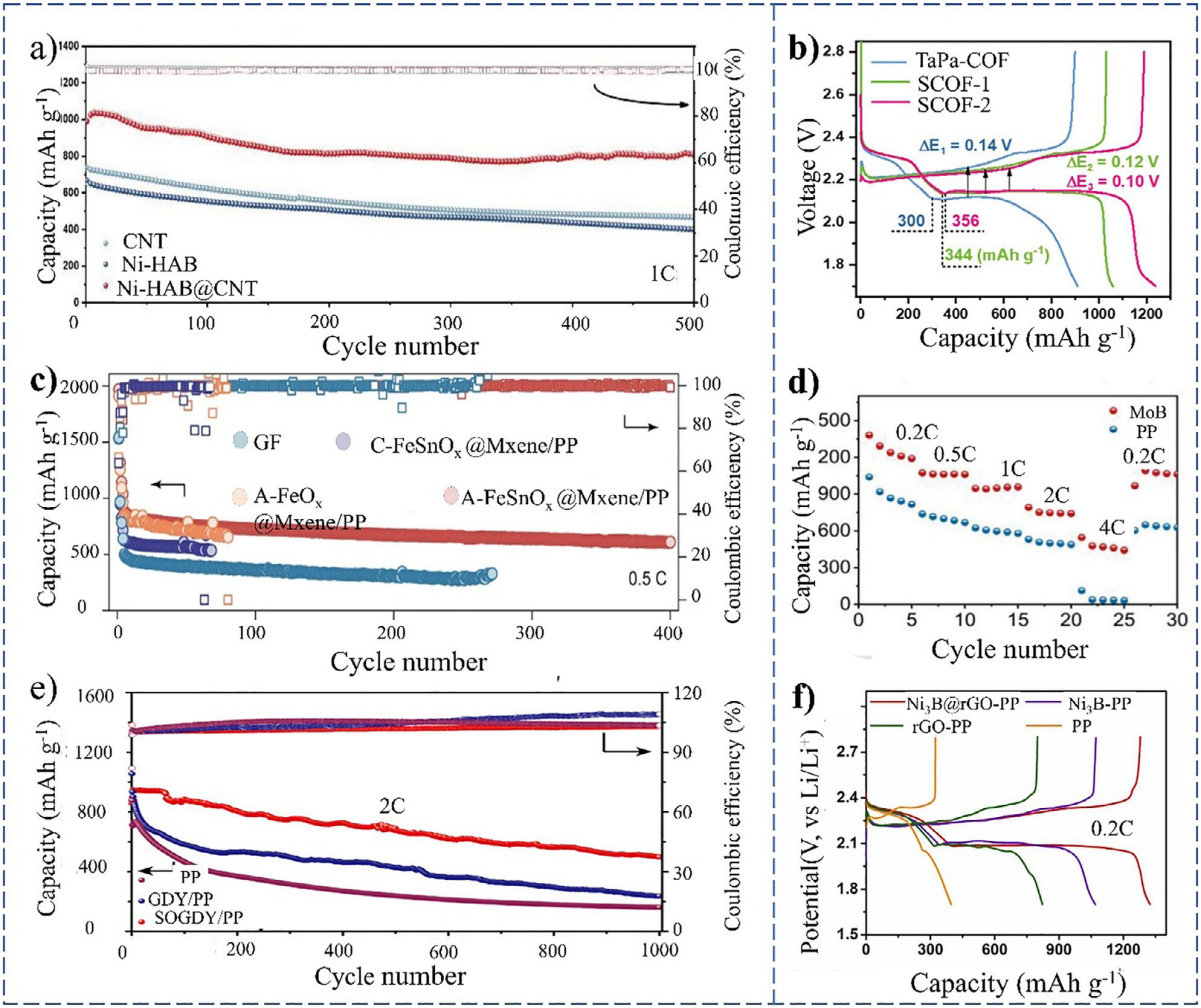
Long‐term cycling stability (a) CNT, Ni‐HAB and Ni‐HAB@CNT at 1 C (reproduced with permission from ref. [324].Copyright 2023,Wiley); (c) FeO_x_@MXene/PP, A‐FeSnO_x_@Mxene/PP at 0.5 C (reproduced with permission from ref. [327].Copyright 2024,Wiley); (e) PP, GDY/PP and SOGDY/PP at 2 C (reproduced with permission from ref. [328] Copyright 2023,Wiley); Rate performance of (b) charge‐discharge voltage profile at the current 0.1 C, (reproduced with permission from ref. [325]. Copyright 2021, Wiley); and (d) Rate performance of MoB and PP from 0.2 to 4 C (reproduced with permission from ref. [232]. Copyright 2025,Wiley); and (f) galvanostatic discharge‐charge profiles at the first cycle of Ni_3_B@rGO‐PP, Ni_3_‐PP, rGO‐PP and PP separator (reproduced with permission from ref. [329]. Copyright 2022, Elsevier).

**FIGURE 15:**
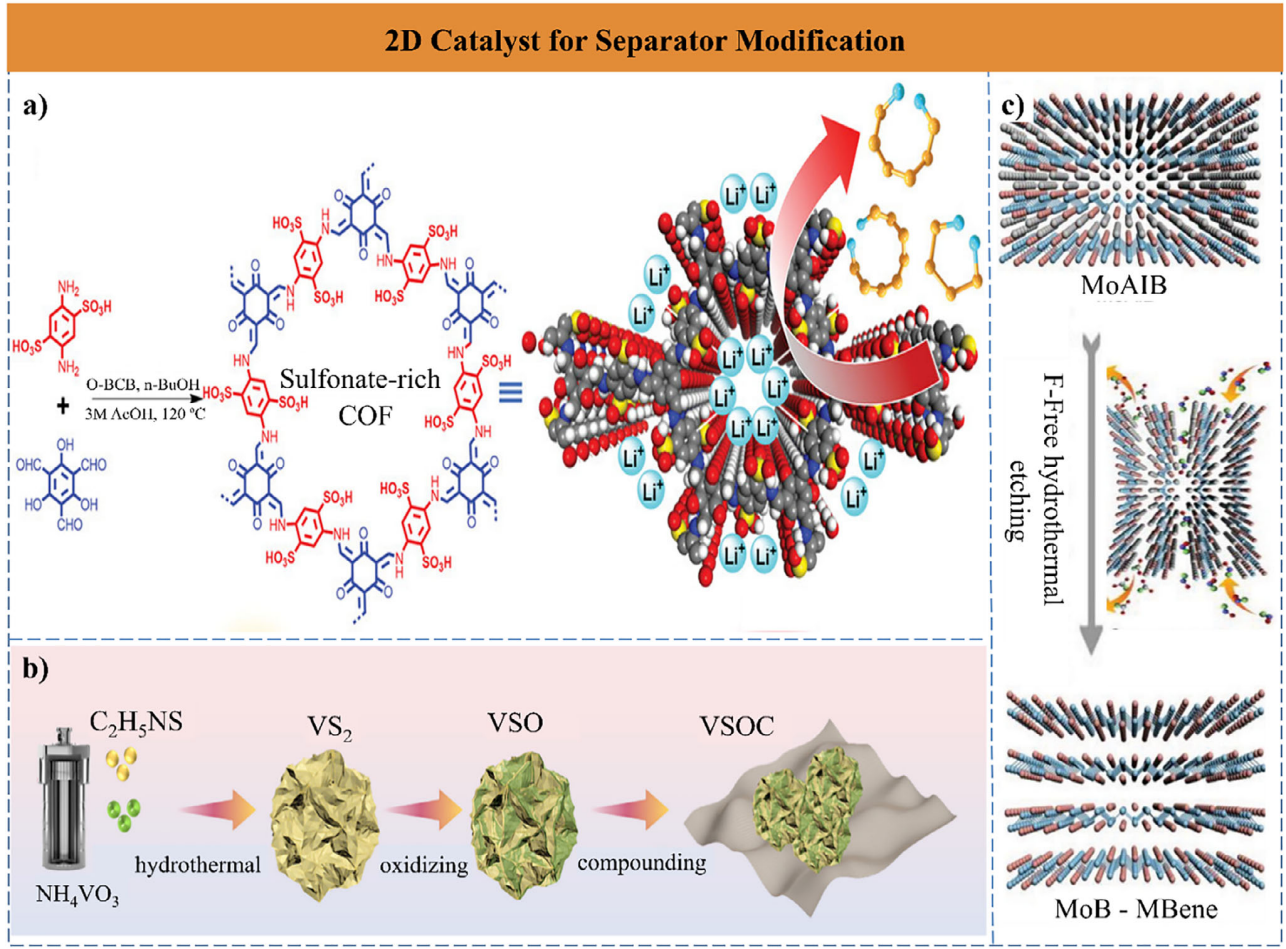
(a) Synthesis scheme of sulfonated COFs, selective permeability of Li^+^ and the blocking of polysulfides in Li–S batteries (reproduced with permission from ref. [325]. Copyright 2021, Wiley); (b) graphical illustration of VSOC synthesis (reproduced with permission from ref. [338]. Copyright 2025, American Chemical Society); and (c) schematic of a modified Li–S cell with MoB/separator (reproduced with permission from ref. [232]. Copyright 2025, Wiley).

Fabricating 2D materials on separators inhibits polysulfide migration through physical barriers and chemical adsorption. For example, graphene's hydrophobic basal plane and its abundant oxygen‐containing functional groups (e.g., hydroxyl ‐OH and carboxyl ‐COOH) foster the chemical adsorption of polysulfides and retain electrolyte permeability [318, 319, 320]. Similarly, g‐ C_3_N_4_ provide rich sites for polysulfide binding. On the other hand, 2D materials with highly ordered, uniform pore structures selectively allow the metallic cations and restrict larger polysulfide anions [139, 321]. For example, the diameters of Li and Na ions are only 1.52 and 2.04 Å, respectively, whereas the VDW diameters of higher‐order polysulfides range from 10.99 to 19.19 Å, indicating that pore sizes optimized around or slightly above the size of the metallic cations yet below the larger polysulfides provide selective filtering [322]. Similarly, intrinsic or functionalized negative surface charges on 2D materials enhance the electrostatic repulsion of polysulfide anions, further mitigating the polysulfide crossover. This repulsive interaction simultaneously promotes a uniform metallic ion flux across the membrane/separator, minimizing localized ionic concentrations and preventing uneven metal deposition and dendrite formation on the anode surface, thereby improving the lifespan of the anodic electrode [323]. For example, 2D crystalline MOFs, exhibit dense d‐sp^2^‐hybridized and display uniform sub‐nanometer pore sizes (∼8 Å). These features physically obstruct polysulfide migration through size exclusion, while the chemically rich functional group will serve as anchoring sites to enhance the polysulfide conversion kinetics. The electrochemical performance was index at Figure 14a [324]. Similarly, Wang et al. designed 2D COF as a dual‐function separator using sulfonate‐rich COF (SCOF‐2) (Figures 14b and 15a). The negatively charged ‐SO_3_^−^ groups electrostatically repel polysulfide anions, dramatically reducing polysulfide crossover to the Li anode. In addition, ‐SO_3_^−^ groups coordinate with Li^+^ and create ordered channels within the COF pores, facilitating selective and fast Li^+^ conduction. As a result, the 2D SCOF‐2 attained a low attenuation rate of 0.047% per cycle over 800 cycles at 1 C. The superior stability stems from its dual‐function mechanism: Initially, the sulfonic groups in 2D SCOF‐2 create a strong electronegative environment that electrostatically repels polysulfide anions. Secondly, 2D SCOF‐2 chemically adsorb molecular LiPSs through Lewis acid‐base interactions, effectively suppressing the shuttle effect and preventing anode corrosion. It is also worth noting that the wide interlayer spacing (4.61 Å) in 2D SCOF‐2 accelerates Li^+^ migration and promotes uniform Li deposition. As a result, it prevents active site degradation during prolonged cycling [325]. On the other hand, MXene delaminated sheet‐like structures harbor acidic Ti surface sites via Lewis acid‐base interactions. These Ti‐S bonds strongly chemisorb polysulfides, enhancing their immobilization and accelerating the sulfur redox kinetics. In an exemplary case, Wang et al. synthesized a hybrid MXene structure and incorporated it into a GF separator. MXenes were synthesized in two layers owing to their large aspect ratios. A large Ti_3_C_2_T_x_ nanosheet was used as the inner layer, followed by a small Mo_2_Ti_3_C_2_T_x_ nanoflake as the outer layer on the surface of the GF separator. Unlike other layered structures, Ti_3_C_2_T_x_ nanosheets with large inner layers effectively blocked polysulfides and chemically confined them, whereas the small Mo_2_Ti_3_C_2_T_x_ outer layer showed superior performance in polysulfide trapping and conversion, thereby accelerating the sluggish kinetic reaction. Owing to Ti_3_C_2_T_x_ superior polysulfide absorption and conversion, the cell with the modified GF separator exhibited an initial reversible capacity of 562 mAh g^−1^ and even after 500 cycles it attained 357 mAh g^−1^ at 1 C [326].

Meng et al. functionalized a PP separator with a titanium carbide (Ti_3_C_2_)‐doped cobalt phosphate nanocage (CPNC) heterostructure. Owing to the advantageous properties of MXene (surface area and functional groups), Ti_3_C_2_ prevents the aggregation of cobalt phosphate (CoP), and fabricated cells with Ti_3_C_2_/CPNC@PP||Ti_3_C_2_/CPNC@Li electrode display a maximum rate performance of 1191.0, mAh g^−1^ at 0.2 C. As a result, Ti_3_C_2_/CPNC heterostructure chemical anchoring Li_2_S_4_ and rapid conversion kinetics for LiPSs throughout prolonged cycling. The expectational stability of Ti_3_C_2_/CPNC heterostructure stems from the high conductivity of Ti_3_C_2_ and CoP he polar nature accelerate Li^+^ transport. As a result, they reduce the Li_2_S_4_ activation energy (15.7 kJ mol^−1^), limiting the accumulation of insulating discharge [330]. The prominent surface terminal groups (‐O, ‐OH, ‐F) on the MXene surfaces contribute to their hydrophilic character and their electrostatic interactions with polysulfides, further suppressing polysulfide migration [331, 332]. Similarly, Junwu Zhu and co‐workers al. designed a MOF by combining a π‐conjugated hexaaminobenzene (HAB) linker and nickel (II) ions as metal nodes. The synthesized 2D Ni‐HAB was deposited on carbon nanotubes (CNT) and used to modify the separator layer. 2D crystalline MOFs, exhibit dense dsp^2^‐hybridized Ni‐N_4_ units and display uniform sub‐nanometer pore sizes (∼8 Å). Further, Ni metal embedded in the HAB ligand acted as a Lewis acid and interacted with the soluble polysulfides. These features physically obstruct LiPSs migration through size exclusion, while the chemically rich Ni‐N centers serve as strong anchoring sites that enhance the p‐d adsorption and conversion kinetics. These designs traps LiPSs and facilitates its decomposition during sulfur reduction and evolution reactions, resulting in long‐term stability with 85.2% capacity retention even after 200 cycles at 0.2 C. The superior stability stems from the planar dsp^2^‐hybridized coordination of Ni‐N_4_ active sites. As a result, they chemically adsorb LiPSs through Lewis acid‐base interactions and accelerate their bidirectional conversion kinetics. Further, 2D Ni‐HAB sub‐nanometer pores (≈8 Å) physically block polysulfide migration without hindering Li^+^ transport. It is also worth noting that the Ni‐HAB metallic conductivity (90 ± 15 S m^−1^), along with a conductive CNT network, accelerates electron transfer, without active site degradation during prolonged cycling. Furthermore, in situ grown Ni‐HAB nanosheets anchored on CNT prevent agglomeration and preserve abundant active sites [324]. The distinctive features of 2D materials, such as the high pore size and substantial functional groups, enable strong chemical and physical interactions with polysulfides, thereby serving as effective barriers in reducing polysulfide shuttling [333, 334, 335]. A research group led by Liu et al. utilized metal selenides (Co‐Se_2_) and C_3_N_4_ to modify the PP separator. The metal selenides exhibited metal‐like conductivity and formed strong S‐Co and Li–Se bonds with LiPSs, reducing the nucleation barrier for Li_2_S. Due to the catalytic activity of CoSe_2_ and its abundant nucleation sites, a uniform deposition of Li_2_S was achieved. The high nitrogen content of g‐C_3_N_4_ facilitated the adsorption of LiPSs and enhanced their conversion, thereby effectively blocking their migration. Furthermore, owing to its layered structure, it accommodates the volume expansion caused by the formation of low‐density Li_2_S during the discharge process. The addition of g‐C_3_N_4_ to metal selenide displayed a unique electron cloud distribution and benefited from the C_3_N_4_‐CoSe_2_ heterointerface; an internal electric field was generated. The generated electric field accelerated the charge flow along with LiPSs conversion. The lithiophilic g‐C_3_N_4_‐CoSe_2_ separator regulated the uniform distribution of Li metal, circumventing the formation of Li dendrites [336]. Similarly, copper‐doped graphitic carbon nitrate (Cu‐g‐C_3_N_4_) catalysts featuring 2D lamellar structures were synthesized by the Xianyou Wang group and used to modify LSBs membranes. Cu is uniformly dispersed over the abundant triple‐s‐triazine units of g‐C_3_N_4._ Hybridization between the Cu d orbitals and sulfur p orbitals of the Cu‐g‐C_3_N_4_ catalyst promoted electrocatalytic activity by generating extra active sites and accelerating the redox kinetics of LiPSs for simultaneous S reduction and Li_2_S oxidation. Consequently, the cell utilizing Cu‐g‐C_3_N_4_‐based separator, attained a high areal capacity of 6.04 mAh cm^−2^ with an S loading of 5.10 mg cm^−2^ [337]. ling et al. constructed (VS_2_‐VO_x_/V_2_C) heterostructure to address the polysulfide shuttle in LSBs (Figure 15b). In VS_2_‐VO_x_/V_2_C heterostructure, 2D VS_2_ possesses large interlayer spacing (5.76 Å), which is beneficial for ion intercalation. Further, the metallic V_2_C provides open and conductive 2D ion‐transport channels, which reduce the Li^+^ diffusion path and improve kinetics significantly. As a result, LSB attained low polarization and showed high lithium‐ion diffusion coefficients and superior rate performance (501 mAh g^−1^ at 5C). This might be due to the low dissociation energy barrier (0.43 eV) of Li_2_S_4_ to Li_2_S conversion, hindering the accumulation of insulating discharge products. The in situ formed V‐S‐O heterointerface offers high active sites with strong bidirectional catalytic activity, while the robust V_2_C conductive network maintains structural integrity and prevents material degradation during prolonged cycling [338].

Yao et al. utilized MBene separator in Li–S cells (Figures 14d and 15c). Owing to the polar Mo and B surface sites on MBene, MBene displayed strong chemical affinity for LiPSs and reduced their crossover. In addition, the metallic conductivity of MBene and its strong Mo‐B motifs accelerate LiPSs conversion, lowering the overpotential and improving the rate behavior. Furthermore, MBene lamellar 2D sheets create a thin, tortuous barrier that hinders anion diffusion while allowing Li^+^ transport [232]. On the other hand, Jun Pu and co‐workers designed a WB‐based MBene with boron vacancies using a fluorine‐free, room‐temperature etching route. Boron vacancies expose more W active sites and shift the d‐band center, strengthening Li‐polysulfide (LiPSs) adsorption and accelerating sulfur redox kinetics. As a result, the WB‐VB modified separator shows superior stability over 500 cycles at 2 C with 0.069% capacity decay. The superior stability stems from the dual‐site interaction between W and B atoms with LiPSs. Initially, these boron atoms offer strong Lewis acid‐base anchoring through electron‐deficient B‐S bonds, while W atoms offer catalytically active sites for rapid conversion, and the introduction of B‐vacancies shifts the d‐band center of W upward, exposing more active sites and enhancing LiPS adsorption and conversion kinetics without compromising structural integrity. In situ x‐ray absorption spectroscopy confirms that the WB‐VB phase remains stable throughout cycling, with W‐B and W‐W coordination bonds maintained at 1.93 and 2.8 Å during charge‐discharge, while TEM shows the nanosheet morphology preserved after cycling with no observable phase degradation [339].

Beyond traditional polysulfide‐blocking strategies, 2D material accelerate the interfacial desolvation (Li^+^/Na^+^) prior to redox reactions [340]. For instance, polar active sites in 2D material weaken ion‐solvent interactions, while their sub‐nanometer pores physically sieve solvated ion clusters. Additionally, the formation of heterojunction interfaces promotes charge redistribution and lowers the desolvation energy barrier. For instance, Zhang et al. designed porous nitrogen‐doped carbon‐grafted cobalt phosphate (CoP) nanosheets (EPDB‐CgCP) and coated on PP separator. The EPDB‐CgCP‐coated separator limit polysulfide migration, and it also accelerates interfacial Li^+^ desolvation prior to sulfur redox reactions. As a result, N‐Co‐P active sites in EPDB‐CgCP catalyst catalyze and weaken Li+ solvent interactions. Further, its sub‐nanometer pores (≈0.2–1 nm) sieve large solvated Li+ clusters, thereby releasing more Li+. Consequently, the fabricated device showed a capacity of 3.2 mAh cm‐2 under a high sulfur loading of 5.7 mg cm^−2^ [341]. We believe that these 2D materials impede the diffusion of polysulfides (M_2_S_x_, x = 4–8) and mitigate the shuttle effect through physical confinement and catalytic conversion.

#### 5.3.2 Thermal and Mechanical Stability

**FIGURE 16:**
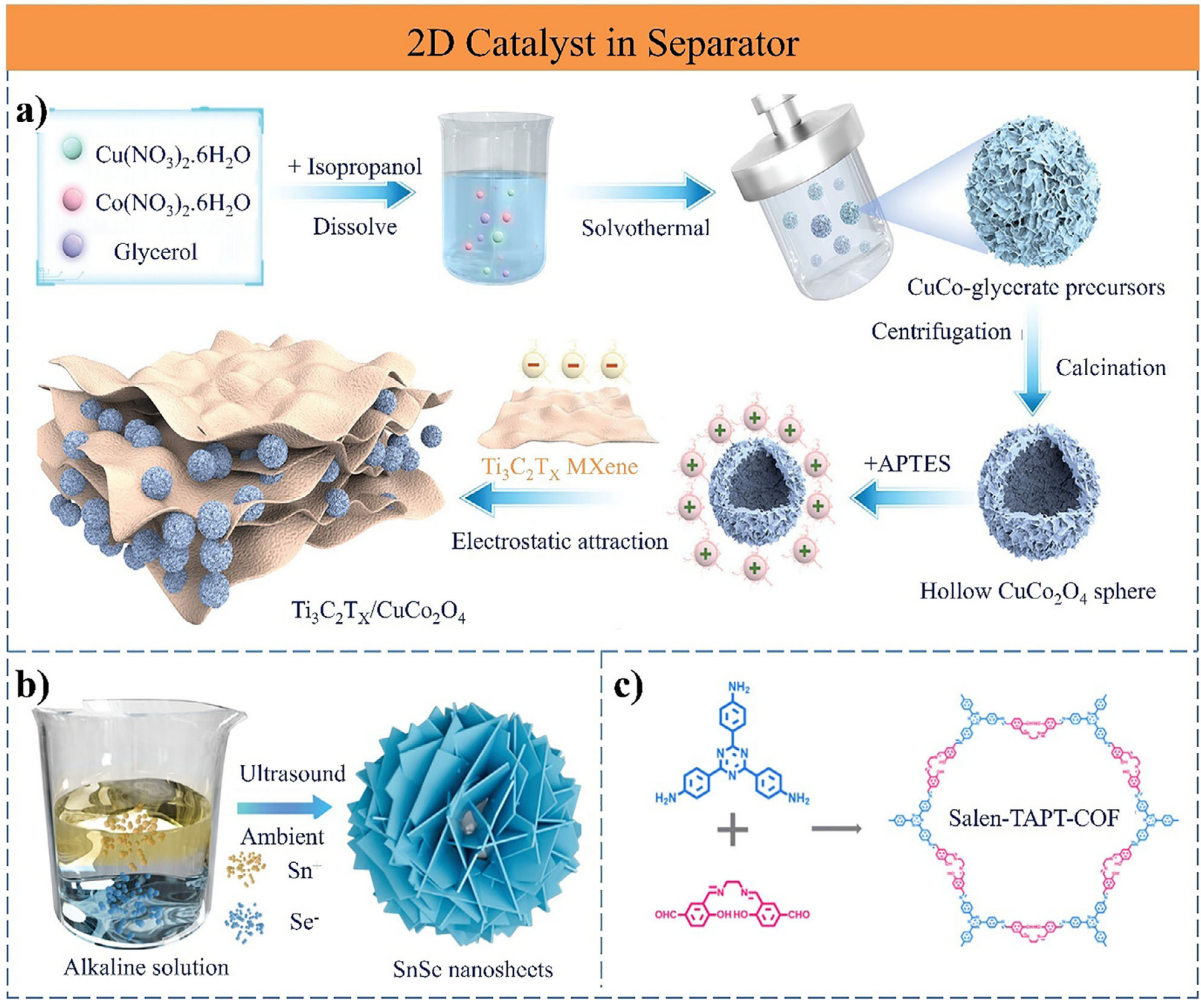
(a) Graphical representation of MXene/CuCO_2_O_4_ preparation (reproduced with permission from ref. [345]. Copyright 2025, Wiley); (b) graphical representation of incorporating SnSe nanosheets in MSBs within the separator(reproduced with permission from ref. [351]. Copyright 2024, Wiley); (c) graphical illustration of Salen‐TAPT‐COF preparation(reproduced with permission from ref. [138]. Copyright 2025, Royal Society of Chemistry).

Conventional separators often suffer from thermal shrinkage, mechanical tearing, or pore collapse under high current densities, elevated temperatures, or prolonged cycling, leading to internal short‐circuiting and thermal runaway. To address these issues, 2D material‐coated separators have emerged as a promising strategy. Tang et al. coated a graphene layer on a PP separator, which acted as a heat sink to eliminate local hotspots in the initial stage of dendrite formation. The graphene‐coated PP separator effectively prevents localized heat accumulation caused by uneven metallic ion deposition. ​This thermal‐management capability prevents dendrite growth by dissipating heat more evenly, and the graphene layer enhances the overall stability of battery performance during charge/discharge cycle. Consequently, the fabricated cell with a graphene/PP separator using Li||Cu electrodes displayed a maximum CE of > 95% after 250 cycles at 1 mA cm^−2^. In addition, the cell without the graphene‐modified separator exhibited a maximum CE of ∼65% [342]. Jiang et al. modified a PP separator using a Fe_3_S_4_/rGO composite. As a result, the fabricated cell attenuated an initial capacity of 1293 mAh g^−1^ at 0.2 C and retained 750 mAh g^−1^ after 100 cycles [343]. Similarly, Zheng et al. constructed 2D porous carbon nanosheets on a PP separator. Benefiting from the high mechanical properties of the laminar structure, the fabricated separator sustains for the long run [344]. Jianmin Zhang modified pp separator using 2D Ni_3_B@rGO. The boron site provides an active site and modulates the unique electronic structure of the Ni_3_B@rGO coating layer, which efficiently improves the polysulfide adsorption ability in Li–S batteries. Furthermore, the modified separator exhibited thermal stability at 145 °C for 3 h and demonstrated superior mechanical properties and superior electrochemical performance (Figure 14f) [329]. Researchers have explored a variety of 2D materials to improve the thermal and mechanical stability of the separator. Zhang et al. constructed Ti_3_C_2_T_x_ MXene@CuCo_2_O_4_ (MCC) composite over PE separator (Figure 16a).  The metallic conductivity of 2D MXene provide a lamellar barrier with tortuous ion pathways. This continuous pathway provides high electron transport and blocks LiPSs. Further, the anchored CuCo_2_O_4_ introduce chemisorptive sites that fasten their solid–liquid–ssolid conversion. As a result, 2D/0D heterointerfaces accelerate the Li_2_S deposition and fasten its decomposition [345]. Bae et al. fabricated a multifunctional separator composed of deoxyribonucleic acid (DNA)‐carbon nanotube (CNT) and Ti_3_C_2_T_x_ MXene hybrid‐modified PP (denoted as DNA‐CNT/MXene/PP) via a facile vacuum filtration approach. The synergistic interactions among DNA, CNT, and MXene in the modified PP separator physically hinder and chemically anchor the shuttled polysulfides and show enhanced electrolyte wettability. This coating also provides high‐temperature tolerance (even at 180°C) and flame retardancy. Consequently, the Li–S batteries with the modified PP separator showed a superior ability to operate over a wide temperature range of 25°C–100°C [346].

Theoretical calculations state that the GDY uniform pore distribution (5.24 Å) enables a fast Li ions transport channel, and also GDY has superior mechanical stability, which is anticipated to prevent thermal damage [347, 348]. In a typical example, Huang et al. described the in situ synthesis of GDY nanosheets on a commercial PP separator. Leveraging the pore size and interlayer distance of GDY, the cell with a modified separator showed a capacity of 412 mA h g^−1^ even after 500 cycles [349]. For comparison, various 2D modified separators were summarized in Table 6. To increase the mechanical stability of PP separator, Li et al. functionalized GDY with sulfonic acid. Computer simulations indicate that, to transport Li ions in GDY pores, the Li ions should undergo an exothermic process to the lowest energy state, followed by an endothermic process to overcome a migration energy barrier of 1.83 eV. In the case of SOGDY, Li ions undergo an endothermic process to overcome an energy barrier of 0.80 eV and an exothermic process. Furthermore, the absorption of LiPSs by GDY is attributed to the rich acetylene bonds, whereas in the case of SOGDY, the spatial obstruction and strongly polar adsorption sites originating from sulfonic acids (S═O and S─O) effectively inhibit the shuttling of LiPSs. As a result, the fabricated device, even at 3 C and ‐10°C, exhibited a high initial specific capacity of 804.5 mA h g^−1^ and a high final capacity of 504.9 mA h g^−1^ after 500 cycles (Figure 14e). This exceptional stability stems from its sulfonic acid groups and its pore structure (5.42 Å). Initially, the sulfonic groups foam strong polar adsorption sites (S═O and S─O) that hinder LiPS shuttling through spatial obstruction and chemical trapping. Whereas its pore structure establishes fast Li^+^ transport and prevents dendrite growth throughout prolonged cycling [328]. This result highlights the enhanced mechanical stability of the separator. Similarly, to modify the PP separator, Zhao et al. utilized MXene sheets and integrated them with amorphous 2D iron oxide (A‐FeSnO_x_) nanosheets with abundant oxygen vacancies (O_v_s) [327]. In general, oxygen vacancies alter the electronic structure of materials, resulting in unsaturated coordination sites, promoting polysulfides adsorption and sulfur conversion [350]. In this regard, Jie Zhao and their associate increased the oxygen vacancies (O_v_s) of FeO_x_ by introducing the Sn element. The A‐FeSnO_x_‐based separator enhanced the binding energy of the polysulfides and lowered the energy barrier for polysulfide decomposition. Furthermore, the addition of crystalline MXene to the A‐FeSnO_x_ catalyst formed a biomimetic amorphous/crystalline (A/C) interface, which increased the Young's modulus (4.9 GPa) of the separator and hindered dendrite growth. Consequently, the A‐FeSnO_x_@MXene‐modified PP separator could withstand the stress induced by the active material during the plating and stripping cycles. Furthermore, the A‐FeSnO_x_@MXene‐modified PP separator exhibited minimal areal density and a thickness of only 0.1 mg cm^−2^ and ∼200 nm, and exhibited a capacity of 610.3 mAh g^−1^ with an ultralow capacity decay of only 0.013% per cycle after 400 cycles at 0.5 C in Na||Na symmetric cells. The superior stability might be attributed to the increased Ovs concentration due to Sn incorporation. This increased Ovs improves the polysulfide binding energy and accelerates their kinetics, inhibiting the accumulation of insulating discharge products, As a result, A‐FeSnOx limits battery degradation. The electrochemical performance was indexed in Figure 14c [327].

**TABLE 6:** Comparison of electrochemical performance of the 2D modified separator.

Materials	Separator	Capacity mAh g^−1^ Initial/End	Cycle number/C	Capacity retention %	Refs.
Ni‐HAB MOF@CNT	PP	1310/1070	200/0.2 C	81.7	[324]
Sulfonate‐rich TpPa‐COF	PE	759.7/497	800/1 C	65.4	[325]
Ti_3_C_2_T_x_/ Mo_2_Ti_2_C_3_T_x_	GF	562/357	500/1 C	63.5	[326]
Ti_3_C_2_/CPNC	PP	928.4/1150	1150/1 C	54.1	[330]
VS_2_‐VO_x_/V_2_C	PP	1340/1109	100/0.2 C	82.9	[338]
Mxene/A‐FeSnO_x_	PP	1409.6/759.7	200/0.1 C	53.9	[327]
Wb‐Vb MBene	PP	1485/940	200/0.2 C	63.3	[339]
MoB	PP	1376/1084	100/0.2 C	78.8	[232]
C_3_N_4_‐CoSe_2_	PP	1030/715.6	500/1 C	69.5	[336]
Cu‐g‐C_3_N_4_	PP	1182.6/672.31	400/0.5 C	56.9	[337]
Ni_3_B@rGO	PP	1325.1/819	100/0.2 C	61.8	[329]
MXene/CuCO_2_O_4_	PP	1225.2/892.4	100/0.2 C	72.8	[345]
GDY	PP	1396/412	600/1 C	29.5	[349]
SOGDY	PP	804.5/504.9	500/3 C	62.8	[328]
SnSe	GF	1529/1035	50/0.1 C	67.7	[351]
Salen‐TAPT‐COF	PP	929.4/657.8	500/1 C	70.8	[138]
HB/CNT@COF	PP	1310/1116	200/0.2 C	85.2	[352]

Cabot et al. utilized 2D orthorhombic SnSe catalyst to block the polysulfide (Figure 16b). The 2D SnSe surface exposes different electronegativities of Se and Sn, which form polarized sites that strongly adsorb polysulfides and promote their catalytic conversion. These sites adsorb and convert polysulfide. Further, the redox activity of Sn^2+^/Sn^4+^ accelerate electron transfer during sulfur conversion reaction. As a result, SnSe‐modified separator exhibited an ultralow capacity decay of only 0.049% per cycle over 800 cycles at 3 C. This superior stability might be due to the combination of chemical affinity between the polar SnSe surface and polysulfides through Sn‐S bonding. Consequently, these separators trap soluble polysulfides and prevent their shuttling to the alkali metal anode. Further, the electrical conductivity (7 × 10^4^ S m^−1^) and excellent catalytic activity lowers the Li_2_S decomposition energy barrier (0.63 eV on SnSe) and Gibbs free energy for the rate‐limiting Li_2_S_2_→Li_2_S conversion. This leads to complete sulfur conversion without active material accumulation during prolonged cycling [351]. Recently, Zhu et al. fabricated a NaPSs defense system using a freestanding multifunctional COF membrane/hydroxynaphthol blue/multiwalled carbon nanotubes (HB/CNT@COF). Consequently, the negative surface charge on the separator surface inhibited the polysulfide migration into the COF layer. Simultaneously, positively charged Na^+^ ions were rapidly conducted through the COF pores. Meanwhile, the separator, with a rough morphological structure, increased its mechanical stability and provided a strong contact area with metallic Na and delayed Na exfoliation at the interface [324]. Another exemplary case was demonstrated by Wang et al. by introducing an azobenzene side group to the COF molecule. In general, nitrogen lone‐pair electrons tend to coordinate with Na^+^, facilitating Na^+^ migration with reversible coordination bonds via interfacial polymerization. Conversely, the abundance of azobenzene sites in the COF pores acts as an ionic hopping pathway, significantly facilitating Na^+^ migration. The Azo‐TbTh separator exhibited the highest Na^+^ transference number of 0.89, along with an enhanced ion conductivity of 6.9 mS cm^−1^ and the lowest resistance of 1.3 Ω compared to the GF separator. These Azo‐TbTh compounds increase the mechanical stability of the separator, along with its Na^+^ transport characteristics. As a result, this Azo‐TbTh separator displayed a capacity of 1295 mAh g^−1^ at 0.2 C and an ultralow attenuation rate of only 0.036% per cycle over 1000 cycles at 1 C. This superior stability resulted from their covalent bonds, and their electronegative surface (zeta potential = ‐36 mV) provides additional electrostatic repulsion against negatively charged polysulfide anions, effectively suppressing active material loss [352]. Qi et al. coated 2D salen COF on the separator to block the LiPSs (Figure 16c). The extended π‐conjugated systems of 2D salen COF accelerate their in‐plane electron delocalization, resulting in improved electronic conductivity. Further, the negative charge on 2D salen COF repels the polysulfide anions and suppresses their migration [138].

Liqiang Mai synthesized 2D heterostructure (MOF/MXene) to hinder and facilitate the conversion of the soluble polysulfides. The abundant Lewis acidic metal sites in MOF interact with polysulfides and catalyzes their conversion. Furthermore, the terminal g functional group on MXene exhibits strong LiPSs adsorption and forms a strong metal‐S bond, which inhibits the shuttle effect. In addition, the heterostructure‐coated PP separator exhibited strong thermal stability even after exposure to 120°C. These advancements underscore the critical role of the separator in maintaining its thermal and mechanical stability, which increases the lifespan of Li–S battery [353].

### 5.4 Electrolyte Engineering

**FIGURE 17:**
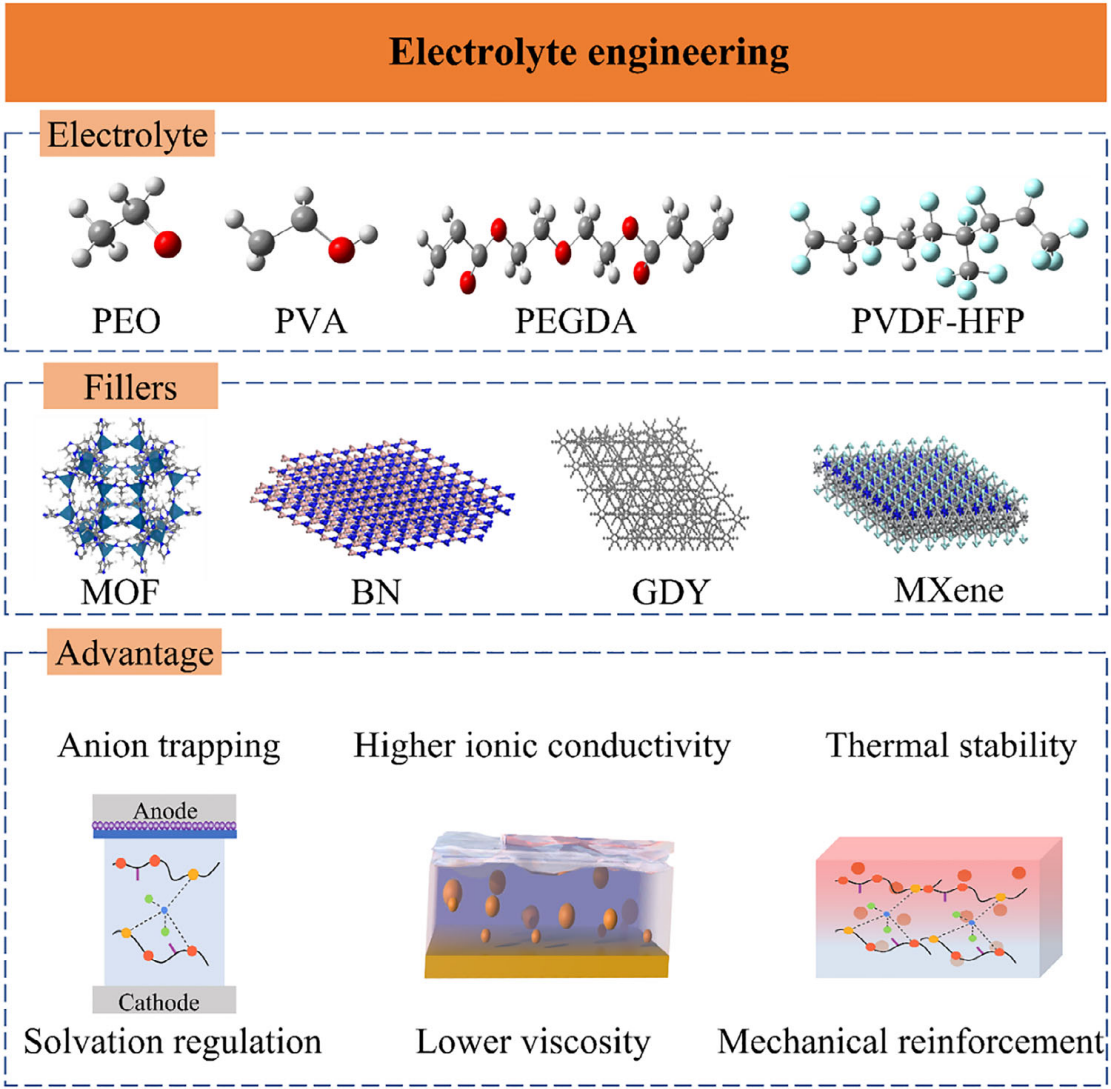
Electrolyte engineering in MSBs. Top (Electrolyte): different type of electrolyte (Solid, gel, polymer) used in MSBs. Middle (Fillers): functional additives‐ceramic perovskites, 2D h‐BN, graphene, and MXene‐like sheets‐that build fast‐ion pathways and mechanically reinforce the film. Bottom (Advantage): synergistic polymer‐filler architecture delivers uniform Li‐ion flux between anode and cathode, suppresses dendrites, lowers interfacial resistance, and enhances mechanical/thermal stability and safety; schematic at right depicts a robust laminar composite membrane.

Electrolyte plays a crucial role in mediating ion transport between the sulfur cathode and metallic anode, directly impacting energy density, cycling life, and safety. State‐of‐the‐art (SOA) electrolytes must combine high ionic conductivity, electrochemical and thermal stability, interfacial compatibility, and mechanical robustness to suppress dendrite formation and prevent electrolyte degradation [280, 281]. To date, several classes of electrolytes have emerged in pursuit of this ideal. Solid polymer electrolytes (SPEs), based on polyethylene oxide (PEO), offer high safety and good electrode adhesion. However, their low ionic conductivity and mechanical strength at ambient conditions hinder practical application. In contrast, gel polymer electrolytes (GPEs) integrate liquid plasticizers into polymer matrices, offering superior ionic conductivity and flexibility. Yet, their plasticized nature can compromise mechanical integrity, increasing the risk of dendrite growth. Lastly, ionic liquid‐based systems embedded in porous or hybrid matrices enable high ionic conductivity and electrochemical stability, forming a third class of quasi‐solid‐state electrolytes. To address these shortcomings, researchers have introduced 2D fillers to improve both ionic transport and to provide mechanical reinforcement capabilities of SPEs [354, 355, 356]. Figure 17 represents different types of electrolytes (solid, gel, polymer) used in MSBs along with various 2D Fillers. With the aid of these fillers, the battery delivers uniform Li‐ion flux between anode and cathode, suppresses dendrites, lowers interfacial resistance, and enhances mechanical/thermal stability and safety. Given the extensive scope of electrolyte systems, this section specifically focuses on the role of 2D materials as fillers in solid polymer electrolytes (SPEs) and gel polymer electrolytes (GPEs). In these systems, 2D fillers modulate ion transport, enhance mechanical properties, and improve interfacial stability

#### 5.4.1 2D Fillers for Ion Transport Modulation

GPEs offer several beneficial characteristics, including high ionic conductivity, non‐combustibility, and conformal interfaces with Na anodes. In addition, the pliability and stretchability of GPEs allow them to accommodate volume expansion. Despite their advantages, the polymer matrix in GPEs impairs mechanical integrity when plasticized with organic electrolytes, increasing the risk of dendrite formation. Additionally, the crystalline structure of the polymer matrix lowers the ionic conductivity and Na mobility and increases the internal resistance of the GPE [357]. To overcome these drawbacks, 2D fillers have been incorporated into GPE matrices. For instance, Xiang Li et al. introduced the photoinitiator benzophenone (BP) and HKUST‐1 into a PEO matrix, referred to as PHGE (PEO + 5.0 wt. % HKUST‐1). As a result, they reduced polarization and showed anion mobility within PHGE, enabling stable performance for over 1000 h. This remarkable stability stems from the high surface area (739.40 m^2^ g^−1^) of HKUST‐1 and its porous structure immobilizes anions. Further, it increased Li^+^ transference number to 0.61, and ionic conductivity (3.48 × 10^−3^ S cm‐1) without compromising the mechanical fluoride (LiF)^−^ rich SEI. As a result, they hinder dendrite growth throughout the cycle [358]. Similarly, Liu et al. added modified zeolitic imidazolate frameworks (ZIFs) in a PEO‐LiTFSI electrolyte. As a result, the PEO‐NH_3_^+^·SO_3_^−^@ZIFs exhibited an ionic conductivity of 4.133 × 10^−5^ S cm^−1^ at 25°C, and the fabricated Li||Li symmetric cell exhibited cycling stability over 4600 h at 0.1 mA cm^−2^. This superior stability stems from the combination of electrostatic interaction and hydrogen bonding. Initially, NH_3_^+^·SO_3_^−^ zwitter‐ion embed TFSI^−^ anions, which increases the Li+ transference to 0.78, which was six times higher than that of PEO‐LiTFSI. Furthermore, the synergistic combination of hydrogen bonding and electrostatic interactions between the ammonia cations and TFSI‐ anions facilitates TFSI dissociation, thereby catalyzing the formation of a lithium fluoride (LiF)‐ rich SEI. As a result, they hinder dendrite growth throughout the cycle [359]. Similarly, to reduce the crystallinity of PEO, Li et al. used CeF_3_ nanoplates as 2D fillers. The Lewis acid‐base interaction between CeF_3_ and PEO ether oxygen reduces its crystallinity. Furthermore, the exposed CeF_3_ [001] crystal faces interacted with the PEO matrix and improved Li transport. In addition, Li‐affinity [100] and Li‐repellent [001] crystal faces of CeF_3_ nanoplates synergistically dissociate the lithium bis(trifluoromethanesulfonyl)imide (LiTFSI), enabling fast Li‐adsorption/desorption and Li^+^ migration. As a result, the CeF3 filler demonstrated high stability over 8000 h in Li/Li symmetric cell [360]. Table 7 summarizes the ionic conductivity of various electrolytes with 2D fillers. Similarly, Wang et al. utilized Fluoride GDY (FGDY) as a 2D filler to enhance the ionic transport in PVDF‐HFP/LiTFSI matrices. This highly ordered 2D FGDY promotes effective LiTFSI dissociation and enhances Li‐ion conductivity (3.29 × 10^−4^ S cm^−1^) and improves the t_Li_^+^ transference number (= 0.79). As a result, PVDF‐HFP/LiTFSI matrices suppress Li dendrite formation through the in situ generation of a LiF‐rich SEI. The resulting Li/FPHL/Li symmetric cell delivered over 1000 h of stable plating/stripping. This superior stability stems from the electric potential synergies in FGDY. It is worth noting that ring‐shaped electron‐rich regions and island‐shaped electron‐deficient regions in FGDY induces LiTFSI dissociation. As a result, they absorb TFSI^−^ anions and provide rapid Li^+^ migration pathways. Further, this FGDY accelerate uniform LiF‐rich SEI that hinders Li dendrite growth. Furthermore, these FGDY nanofillers reduce PVDF‐HFP crystallinity and enhance mechanical strength (ultimate tensile stress of 2.1 MPa) through grain refinement [361]. Zheng et al. blended 2D boron nitride (BN) nanoflakes into a poly(vinylidene fluoride) (PEO‐PVDF)‐lithium bis(trifluoromethanesulfonyl)imide (LiTFSI) polymer. In general, owing to the high porosity of BN nanoflakes, more active sites are connected to the PEO matrix, reducing the crystallinity of PEO. As a result, the BN nanoflakes enhanced the ionic conductivity and mechanical properties and accelerated the heat variation in the PEO‐PVDF electrolyte [88]. Li et al. dispersed 2D Ti_3_C_2_T_x_ into a poly (ethylene oxide)/LiTFSI complex (PEO_20_‐LiTFSI). The hydrophilic terminal group of MXene improves the interaction of the PEO chain and reduces the crystallinity of the PEO matrix. As a result, the MXene‐incorporated PEO_20_‐LiTFSI electrolyte improved the ionic conductivity to 2.2 × 10^−5^ S m^−1^ at 28°C [362]. Recent studies indicate that adding succinonitrile (SN) to a PEO matrix enhances ionic conductivity by reducing PEO crystallinity, promoting lithium‐salt dissociation, and facilitating Li‐ion transport, although excessive SN weakens the mechanical properties of the electrolyte. To address this issue, Tan et al. introduced 2D γ‐Al_2_O_3_ nanosheets into the PEO‐LiTFSI‐SN matrix, where the nanosheets altered the Lewis acid‐base interactions between Li and the PEO‐SN matrix, promoted Li^+^ release, and provided favorable 2D transport pathways, thereby improving both ionic conductivity and mechanical strength. The optimized electrolyte achieved an ionic conductivity of 2.02 × 10^−4^ S cm^−1^ and tensile strength of 0.12 MPa. As a result, the Li||Li symmetric cells displayed stability over 600 h at RT, whereas Li||LFP full cells exhibited a capacity of 127 mAh g^−1^ at 0.5 C with 95% capacity retention after 50 cycles. This remarkable stability arises from the 2D γ‐Al_2_O_3_ nanosheets that provide Li^+^ transport channels along their surface. Further, these 2D nanosheets accelerate long‐range Li^+^ migration with a low diffusion energy barrier (0.75 eV). This results in reduced PEO crystallinity (lowering Tg from ‐34.0°C to −40.6°C) and promotes LiTFSI dissociation through Lewis acid‐base interactions, resulting in improved stability [363].

**TABLE 7:** Highlighting the ionic conductivity of various electrolyte with 2D fillers.

Electrolyte	2D Fillers	Ionic conductivity *S cm^−1^*	Li‐ion transference no	Electrochemical stability 60°C	Refs.
*PEO + LiTFSI*	5 wt % Mxene	7.19 × 10^−4^	*0.51*	5.2	[365]
*PEO‐SPE*	0.7 wt% phosphorene	2.74 × 10^−4^	*0.53*	5.9	[367]
PEO‐ LiTFSI	5.0 wt % HKUST‐1	3.48 × 10^−3^	0.61	5.25	[358]
PEO‐LiTFSI	0.4 wt % NH_3_^+^·SO_3_^−^@ZIF	4.133 × 10^−5^	0.78	5.0	[359]
PEO‐ LiTFSI	10 wt % CeF_3_	3.1 × 10^−4^	0.35	4.8	[360]
PVDF‐HFP/LiTFSI	2.5 wt % FGDY	3.29 × 10^−4^	0.79	4.93	[361]
PEO‐PVDF‐LiTFSI	12 wt % BN	2 × 10^−4^	0.31	5.0	[88]
PEO‐LiTFSI‐SN	0.5 wt % γ‐Al_2_O_3_	2.21 × 10^−3^	0.25	4.87	[363]
PVDF/PEO‐ LiTFSI	3.6 wt % BN	3.03 × 10^−3^	0.6	5.27	[364]
PEO‐ LiTFSI	5 wt % MXene	7.19 × 10^−4^	0.51	5.2 V	[365]
PEO‐ LiTFSI	7 wt% C‐C_3_N_4_	8.26 × 10^−4^	0.69	5.2	[366]

**FIGURE 18:**
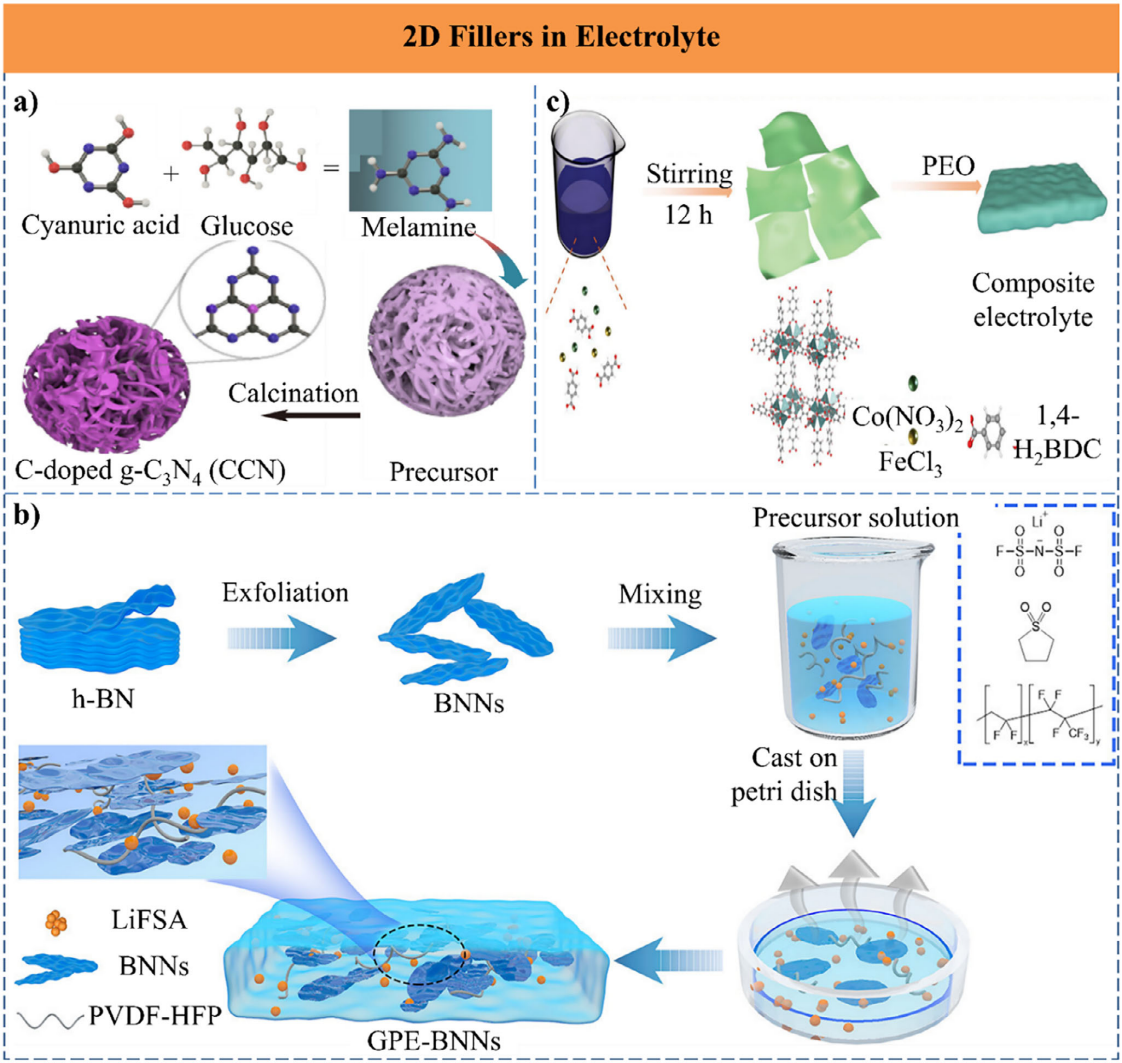
(a) graphical illustration of synthesizing carbon doped C_3_N_4_ (CCN) (reproduced with permission from ref. [366] . Copyright 2025, American Chemical Society); (b) Schematic diagram of the preparation process of 2D BNN‐added gel polymer electrolyte, GPE‐BNNs (reproduced with permission from ref. [372]. Copyright 2025, American Chemical Society); and (c) schematic diagram for preparing MOFs and composite electrolyte membrane (reproduced with permission from ref. [90]. Copyright 2023, Wiley).

Unlike crystalline polymers, amorphous polymeric chains are highly fragile and exhibit poor mechanical stability. To increase the amorphous nature of GPE, Hadis Zarrin synthesized a porous PVDF/PEO binary polymer and doped exfoliated BN nanosheets. The insulating behavior of BN prevents electron crossover and limits anion migration, thereby increasing the Li^+^ transference number. The PEO segments increased the number of amorphous chains in the GPE, resulting in an improved ionic conductivity of 3.03 × 10^−3^ S cm^−1^, whereas the PVDF component improved the stability and mechanical properties of the GPE. As a result, BN‐doped GPE electrolyte in Li||Li symmetric cell displayed cycling over 370 h at 0.2 mA cm^−2^ [364]. Thus, the incorporation of these 2D fillers increased the ionic conductivity in SPEs and GPEs by disrupting the crystalline domains of host polymers (e.g., PEO and PVDF), thereby increasing the amorphous content and enabling faster ion transport. Similarly, Su et al. utilized 2D MXene to improve the ionic conductivity (7.19 × 10^−4^ S cm^−1^) of Ionic Liquid (IL). This 2D MXene not only reduces the crystallinity of PEO but also promotes the dissociation of lithium salts and anchors anions. As a result, the MXene‐incorporated Li||Li symmetric cell exhibited cycling over 650 h at 0.1 mA cm^−2^, whereas Li||LFP full cells attained an initial capacity of 162.2 mAh g^−1^ with 95.3% capacity retention even after 120 cycles at 0.5. This might be due to the improved LiTFSI dissociation and strongly anchored TFSI^−^ anions that resulted from imidazole cations of the ionic liquid. As a result, they suppresses concentration polarization and enhance interfacial stability, enabling uniform Li deposition without dendrite formation [365]. Similarly, Wu et al. prepared C_3_N_4_ and doped carbon atom to enhance the ionic conductivity of SPE. The C‐doped C_3_N_4_ improves the Lewis acid‐base interactions with lithium. This interaction enhances the LiTFSI salt dissociation and improve their concentration. As a result, this they improved Li^+^ Transference Number (0.69), compared to pristine SPE (0.57) (Figure 18a) [366]. Overall, 2D fillers improve the ionic conductivity and enhance the mechanical strength in the electrolyte.

#### 5.4.2 2D Fillers for Mechanical Reinforcement

Beyond ion conduction, mechanical stability is critical for suppressing dendrite growth and maintaining long‐term cycling performance. Incorporating 2D fillers with high aspect ratios and intrinsic stiffness can reinforce the electrolyte framework, increase Young's modulus and tensile strength, and improve its resistance to deformation. For instance, Koduru et al. integrated GO nanosheets into a PEO/PVA composite. Compared with the PEO/PVA electrolyte, the GO‐doped PEO/PVA electrolyte increased the ionic conductivity (1.36 × 10^−6^ S cm^−1^) and mechanical properties, including Young's modulus by 1.4 times and tensile strength by 1.8 times [368]. Another exemplary case was demonstrated by Zhou et al. by introducing 2D Co‐MOF in PVDF/ LiTFSI. Due to 2D Co‐MOF Lewis acidity and 2D porous nanostructure, 2D MOF interacts with the PVDF/LiTFSI matrix. As a result, 2D Co MOF incoportaed PVDF/ LiTFSI matrix accelerated LiTFSI dissociation and reduced PVDF crystallinity. The enhanced composite solid electrolyte displayed an excellent Li^+^ transfer number of 0.70 and a high ionic conductivity of 6.26 × 10^−4^ S cm^−1^  [369] Similarly, Jiayan Luo and co‐workers utilized 2D vermiculite clay sheets to enhance the PEO properties. The integration of 2D vermiculite clay sheets decreased the crystallinity of the PEO matrix. Moreover, the high Young's modulus of vermiculite clay sheets (175 GPa) effectively inhibits the growth of Li dendrites. In addition, the tensile strength of the SPE doubled from 0.41 to 0.8 MPa. Consequently, the larger tensile modulus of the PEO matrix delayed the nucleation of Li dendrites and prevented electrode cracking [370].  Wang et al. designed solid electrolyte (PEO‐LiTFSI) with stacked 2D vermiculite (Vr) nanosheets. The incorporation of continuous 2D interlayer channels improved the PEO chain motility and LiTFSI dissociation, resulting in a superior ionic conductivity of 1.22 × 10^−5^ S cm^−1^ at 25 C. Furthermore, the strong rigidity of the vermiculite nanosheets imparted to Vr/PEO‐LCSE an excellent compressive strength of 131 MPa, which is 550% higher than that of pure PEO, resulting in improved performance of LSBs [371]. Ueno et al. tuned the mechanical and thermal properties of a sulfolane‐based GPE with few‐layer 2D boron nitride (h‐BN) nanosheets. The rigid, thermally conductive BN network reinforced the polymer matrix, smoothing the current distribution at the Li interface. Furthermore, the dispersed h‐BN sheets interact with the anion, slowing anion motion more than Li^+^ and promoting selective Li^+^ transport. As a result, 2D h‐BN fillers deliver a safer, tougher, and more Li^+^‐selective gel that mitigates dendrites and boosts cycle stability (Figure 18b) [372]. Wang et al. designed an interpenetrating polymer network by chemically grafting 2D boron nitride nanosheets and poly(ethylene glycol)diacrylate (PEGDA) using a silane coupling agent. As a result, the 2D BNN‐integrated SPE electrolyte generates vast amorphous regions for fast Li‐ion immigration and improves the ionic conductivity without sacrificing mechanical stability. Mechanical stability was retained owing to covalent coupling. These Covalent bonds at the polymer‐filler interface prevent slippage/voids and remain tough under cycling [373]. Zou et al. synthesized 2D CoFe MOF as functional fillers in PEO solid electrolytes (Figure 18c). This filler provides fast Li^+^ transport channels, and the high surface area of 2D CoFe MOF improves the polymer interaction, which helps in reducing the PEO crystallinity. Further, the electrostatic interaction of 2D CoFe MOF via the anion confinement effect boosts the Li^+^ transference number from 0.36 to 0.64. As a result, the elastic modulus and Young's modulus of 2D CoFe MOF‐incorporated electrolyte show a significant improvement compared to the pure electrolyte [90]. Xu et al. explored the properties of a PH host with a 2D HKUST‐1 filler (PH@MOF). The resulting PH@MOF electrolyte enhanced the movement of the polymer chains, thereby promoting the Na diffusion kinetics and anion anchoring owing to their negative charge. Compared with the standard PH electrolyte, the PH@MOF electrolyte exhibited an improved ion transference number (0.42 to 0.64), high ionic conductivity (9.11 × 10^−5^ to 2.52 × 10^−4^ S cm^−1^) and an electrochemical stability window (4.54–4.73 V). Furthermore, the PH@MOF electrolyte increased the tensile strength to 21.68 MPa and prolonged the nucleation time of Na dendrites, resulting in an improved specific capacity of 113.3 mA h g^−1^ at 1 C [374]. Incorporation of these fillers allows polymer chains to anchor more effectively while distributing mechanical stress, thereby minimizing electrolyte deformation and preserving interfacial contact under operational stress. Table 8 summarizes the mechanical properties of the various electrolytes with 2D fillers. Altogether, 2D fillers across electrolytes improve the ionic mobility, Li^+^/Na^+^ transference numbers, and mechanical stability.

**TABLE 8:** **S**ummarizes the mechanical properties of the various electrolytes with 2D fillers.

Electrolyte	Fillers	Mechanical properties	Li‐ion transference no	Ionic conductivity *S cm_‐_^1^*	Electrochemical stability 60°C	Refs.
PEO/PVA‐ NaIO_4_	20 wt% GO	σ_t_ = 32.15 MPa, E = 1.7 GPa	—	1.03 × 10^−7^	—	[368]
PEO‐LiTFSI	10 wt% Vermiculite sheets	σ_t_ = 0.8 MPa	0.246	1.2 × 10^−3^	5.35	[370]
PEO‐LiTFSI	60 wt% Vermiculite sheets	E = 131 MPa	0.42	1.22 × 10^−5^	4.9	[371]
PVDF‐HFP‐ LiFSA	1 wt% BN	σ_t_ = 1034 kPA	0.55	0.7 × 10^−3^	4.6	[372]
PEGDA‐LiTFSI	5 wt BN	E = 26.2 MPa,	0.49	1.05 × 10^−4^	5.5	[373]
PEO	10 wt% 2D CoFe MOF	E = 2.40 MPa MPa,	0.64	6.5 × 10^−5^	4.5 V	[90]
PH	9 wt% HKUST‐1	σ_t_ = 21.68 MPa	0.64 (Na)	2.52 × 10^−4^	4.73 V	[374]

## 6 Advanced Tools for 2D Materials

2D materials exhibit unique chemical compositions and anisotropic structures that lead to diverse electrochemical behaviors in MSBs. These variations significantly influence ion transport, sulfur conversion kinetics, dendrite suppression, interfacial stability, and so forth. Therefore, a comprehensive understanding of the underlying reaction mechanisms is paramount for optimizing the performance of MSBs. To uncover these complex mechanisms, researchers have developed advanced analytical tools, including operando techniques and ML‐driven modeling methods.

### 6.1 In Situ/Operando Characterization

2D materials with different physicochemical properties often undergo different reaction pathways in MSB components. To understand the reaction mechanisms and to monitor morphological changes in electrodes, electrolytic decomposition and other dynamic processes, researchers have employed various ex‐situ techniques [375, 376, 377]. For example, ex situ UV–Vis spectroscopy has been used to track polysulfides [378]. Generally, polysulfides exhibit distinct energy levels between the highest occupied molecular orbital (HOMO) and the lowest unoccupied molecular orbital (LUMO), allowing researchers to infer their various states through concentration measurements [379, 380]. Similarly, Scanning Electron Microscopy (SEM) and Transmission Electron Microscopy (TEM) have been used to analyze the electrode morphology [381, 382]. However, these techniques are limited in monitoring real‐time processes, which is crucial for understanding dynamic electrochemical environments. To overcome these limitations, operando techniques have been developed. For instance, operando UV‐Vis spectroscopy enables real‐time monitoring of polysulfide formation. However, at high concentrations, the solution becomes opaque, making it difficult to determine the precise oxidation states of polysulfides [383]. To address these challenges, advanced in situ methodologies have been developed. This section explores recent advances in operando techniques, which play a pivotal role in MSBs research. These methods allow researchers to monitor the entire reaction process with a high temporal resolution, particularly focusing on polysulfide conversion.

#### 6.1.1 X‐Ray‐Based Spectroscopic Techniques

**FIGURE 19:**
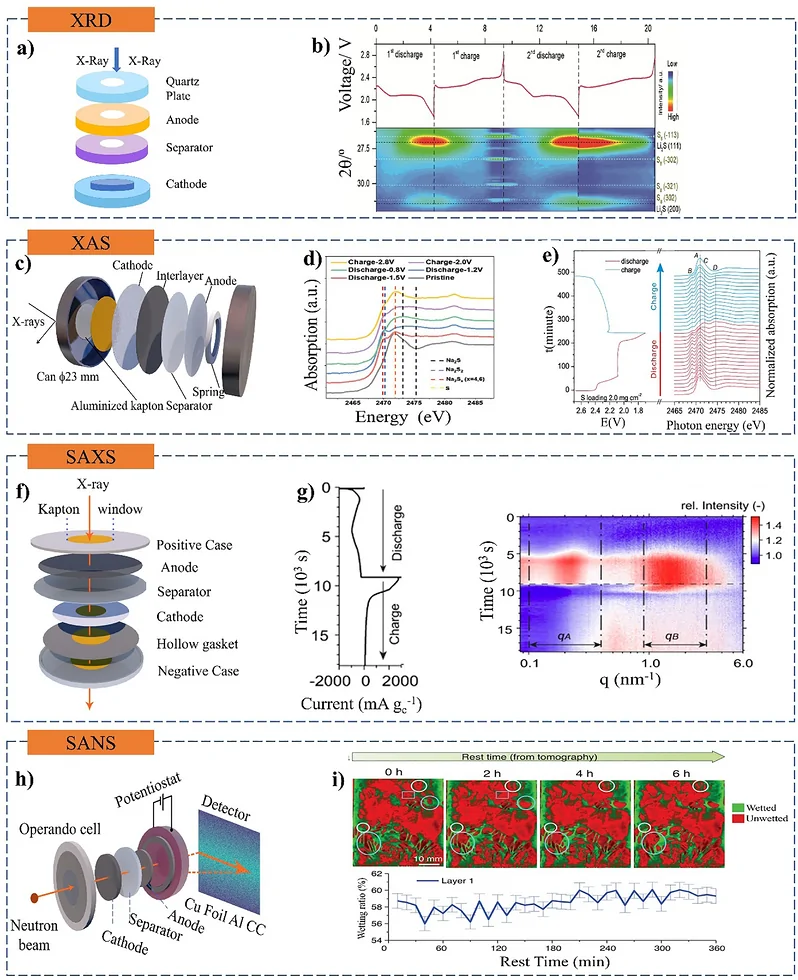
(a) Systematic view of an operando XRD, (b) operando XRD spectra during the charge/discharge charge (reproduced with permission from ref. [82]. Copyright 2022, Wiley), (c) Systematic view of an operando XAS system, (d) XAS spectra of S in various states during the first charge/discharge in MMPCS‐800@S electrode (reproduced with permission from ref. [393]. Copyright 2022, Wiley); (e) SAC doped graphene (reproduced with permission from ref. [240] Copyright 2022, American Chemical Society); (f) in situ SAXS configuration using a Kapton window, (g) analysis of internal structural changes during cycling process in C/S electrode (reproduced with permission from ref. [399]. Copyright 2022, Springer Nature); (h) operando SANS setup to probe lithiation mechanisms and (i) (top) vertical slices of the operando neutron tomograms of the Li–S pouch cell (layer 1) for wetting from 0, 2, 4 to 6 h. Marked aqua circles: locations for merged non‐wetted regions; aqua rectangles: locations for disappeared boundaries; white circles: locations for diminished non‐wetted regions and enlarged wetted regions and (bottom) their quantitative curves of wetting ratio for layer 1 during rest (reproduced with permission from ref. [400]. Copyright 2025, Wiley).

In situ x‐ray diffraction (XRD) tracks real‐time crystal structure changes in electrode materials during battery cycling, revealing phase transitions and lattice strain as ions intercalate (Figure 19a). Operando XRD is useful in sulfur batteries for detecting the formation and dissolution of crystalline S_8_ and Li_2_S phases during sulfur redox reactions. For instance, Xu et al., utilized in situ XRD on MoS_2_ nanodot porous carbon cathodes. As shown in Figure 19b, Xu et al., observed reversible transitions between crystalline Li_2_S and monoclinic S_8_ during cycling, confirming effective catalytic conversion [82]. However, soluble polysulfide intermediates are largely amorphous and evade direct detection, XRD is often paired with XAS and XRF for a complete structural and chemical picture. X‐ray photoelectron spectroscopy (XPS) is employed to monitor the surface chemistry of battery materials [384]. However, due to its limited photon energy range (1‐5 keV), it has a restricted penetration depth, confined to top few nanometers [385]. To address this, x‐ray Fluorescence (XRF) has emerged as a complementary technique. Unlike XPS, XRF operates at higher energies (20‐25 keV), enabling deeper penetration and spatial mapping of the elemental distributions in electrodes [386]. In XRF, when an atom is irradiated, inner‐shell electrons are ejected, and outer‐shell electrons fill the vacancies, emitting characteristic x‐rays. These signals provide insights into elemental composition, distribution, and chemical states [387]. In the context of MSBs, operando XRF has been widely employed to investigate the spatial distribution of sulfur species during charge and discharge cycles. For example, Siebel et al. demonstrated that Li_2_S was present in both electrode regions and remained dispersed within the electrolyte even at the end of the discharge cycle. The Li_2_S formation was confirmed through fluorescence mapping, wherein the fluorescence reduction factor (F_red_) was used to differentiate sulfur species: If F_red_ ≪ 0, Li_2_S was formed; whereas Fred ≫ 0, elemental S_8_ was present; and when Fred ≈ 0, a mixed region of different polysulfide species existed [388]. Similarly, Qing et al. utilized operando XRF to monitor morphological changes and the redistribution of sulfur and polysulfides in Li–S batteries [389]. Despite its utility, XRF suffers from limited chemical sensitivity and the inability to distinguish between elemental sulfur and intermediate polysulfide species. These shortcomings highlight the need to combine XRF with complementary analytical methods, such as x‐ray absorption spectroscopy (XAS), which provide more precise information on the oxidation states and local chemical environments of sulfur species within the battery system. XAS is a powerful technique used to investigate the local structural and electronic properties of materials at the atomic and molecular levels (Figure 19c). The effectiveness of XAS stems from its ability to utilize a broad range of photon energies, typically from 5 to 1000 eV, allowing researchers to probe material interactions. When the energy of an incident x‐ray photon exceeds a specific threshold, a core electron is excited to an unoccupied electronic state, resulting in the formation of element‐specific absorption edges (AEs). These edges are characteristic of different elements, making XAS inherently suitable for element‐selective analyses. XAS spectra are generally divided into two main regions: X‐ray Absorption Near‐Edge Structure (XANES) and Extended x‐ray Absorption Fine Structure (EXAFS). The XANES region typically spans below 20 eV and above 50 eV of the absorption edge. XANES is primarily influenced by the single‐electron and multiple‐scattering effects of the inner‐shell photoelectrons. As a result, XANES provides valuable insight about its oxidation states and local symmetry around the absorbing atom. By transitioning to the EXAFS region, the photon energies can be extended up to 1000 eV, thereby facilitating a deeper investigation of the structural characteristics of the material. EXAFS focuses on determining structural information, such as short‐range order and coordination numbers, which are critical for understanding the interactions between atoms. This information is systematically gathered through variations in the absorption intensity, which reflects the interference of photoelectron waves scattered from neighboring atoms [390, 391, 392]. In the context of MSBs, in situ x‐ray XAS is a powerful tool for distinguishing between various sulfur species by analyzing the local electronic structure and oxidation states of the sulfur atoms. Additionally, in situ XAS enables the evaluation of the electronic properties of the associated 2D host materials. A recent study by Wang et al. utilized operando XAS to track polysulfide evolution during charge‐discharge cycles by analyzing the sulfur K‐edge spectra. The absorption edge appeared at approximately 2465 eV and gradually shifted to 2480 eV. Through spectral deconvolution, the authors identified a peak at 2472 eV, corresponding to S_8_. During the discharge process, a weak signal was also observed at this energy, indicative of intermediate Na_2_S_x_ (x = 4, 6). It is worth noting that a distinct peak at 2470.2 eV was assigned to Na_2_S_2,_ and a peak at 2475.2 eV was assigned to the final discharge product, Na_2_S. Furthermore, the intensity of the Na_2_S_4_ signal periodically decreased and increased during discharge and charging cycle, respectively, indicating a reversible redox process involving transient polysulfides (Figure 19d) [393]. In a similar way, Du et al., used in situ S K‐edge XANES to track sulfur speciation in a Co‐N‐graphene (CoNG) Li–S cell. As shown in Figure 19e, Du et al., confirmed early‐stage formation Li_2_S at the onset of discharge [240]. Similarly, Nazar et al. performed linear combination fitting of XANES data using reference spectra for elemental sulfur (2472 eV) and linear polysulfides (S_x_^2−^, where 2<X <6, at ∼2470 eV). Their results revealed that elemental sulfur forms rapidly and is consumed at the expense of transient intermediates, such as S_6_^2−^ and S_4_^2−^, particularly during the early and final stages of the charge‐discharge cycles [394]. It is important to note that the absorption intensity in XAS is proportional to the number of unoccupied p states. When using the fluorescence mode, especially in high‐concentration samples, self‐absorption effects distort the spectra and impact the oxidation state measurement. Self‑absorption tends to dampen and broaden the near‑edge features, leading to an underestimation of oxidation‑state shifts and complicating quantitative analysis of redox changes. Additionally, conventional XAS is limited in its ability to capture fast dynamic processes occurring on shorter timescales [395]. To overcome these challenges, researchers have developed in situ resonant inelastic x‐ray scattering (RIXS) technique.

Resonant inelastic x‐ray scattering (RIXS) combines x‐ray absorption spectroscopy (XAS) and x‐ray emission spectroscopy (XES) to reveal atomic‐scale electronic structures. Initially, an incident x‐ray photon excites a core electron to an unoccupied state, creating a core‐hole intermediate with a lifetime of a few femtoseconds (fs). In the second step, this intermediate state undergoes radiative decay, wherein a valence or core electron fills the core hole and a characteristic photon is emitted. The energy of the emitted photon depends on the energy difference between the final and ground states, providing fine‐scale information on the chemical and electronic environment [396, 397, 398]. A notable application of operando RIXS was demonstrated by Vizintin et al. by analyzing the sulfur K‐edge during the charge‐discharge cycles of Li–S batteries. They compared the RIXS results with the XAS data and observed a pre‐edge peak at ∼2470 eV in the XAS spectrum, corresponding to LiPSs. Interestingly, the RIXS signal appeared approximately 2 eV lower, providing an enhanced resolution. In contrast to XAS, the RIXS spectra showed no interference from sulfate species originating from the LiTFSI electrolyte. This absence of overlapping signals is attributed to the fixed excitation energy, which minimizes the background noise and enhances spectral clarity [398]. Consequently, RIXS offers a distinct advantage over XRF and XAS in complex electrochemical environments.

#### 6.1.2 Structural Scattering Techniques

The incorporation of sulfur into porous carbon frameworks has emerged as a key strategy for overcoming the inherently low electrical conductivity of sulfur [401, 402]. While traditional techniques, such as Thermogravimetric Analysis (TGA) and Brunauer‐Emmett‐Teller (BET) surface area measurements, provide basic information on sulfur content and pore structure, they fall short in offering real‐time insight into the dynamic behavior of sulfur within the host material. To bridge this gap, advanced in‐ situ techniques, such as Small‐Angle x‐ray Scattering (SAXS) (Figure 19f) and Small‐Angle Neutron Scattering (SANS) have been employed [309, 391, 403, 404]. SAXS is a powerful analytical method that utilizes x‐rays to measure elastically scattered intensity as a function of scattering angle, providing nanoscale resolution of electron density variations within the sample. This technique reveals the location of sulfur impregnation within the micropores and mesopores of carbon matrices. In addition to sulfur mapping distribution, SAXS provides insights into nanoparticle aggregation and the electrolyte diffusion behavior [391]. For instance, Kim et al. used SAXS to track the migration of sulfur in a micro/mesoporous carbon electrode. Owing to the comparable electron densities of carbon (17.00 × 10^−6^ Å^−2^) and sulfur (17.90 × 10^−6^ Å^−2^), contrast analysis enabled the researcher to observe the lithiation behavior of sulfur. This behavior suggests a dual role: micropores serve as sulfur reservoirs, while mesopores act as active reaction zones in Li–S batteries [405].  Similarly, Mascotto et al. studied the sulfur distribution in carbon/sulfur (C/S) nanocomposites. SAXS analysis revealed that the inner surface area derived from scattering measurements was lower than that obtained via the BET analysis. The results indicated that sulfur preferentially occupied micropores at lower loadings but began to fill mesopores as the sulfur content increased, underscoring the critical role of pore architecture in designing efficient cathode materials for Li–S batteries [406]. Additionally, SAXS uses a complementary subset technique Wide‐Angle x‐ray Scattering (WAXS), which provides structural information. WAXS enables the investigation of crystalline structure, lattice parameters, atomic arrangements, phase identification, and surface morphology, including surface roughness [407]. Wood et al. utilized SAXS/WAXS studies to observe the formation of amorphous Li_2_S_2_ particles upon discharge in non‐aqueous Li–S batteries with liquid electrolyte solutions. This study underscores that electrode performance is constrained by mass transport through these intermediates (Figure 19h) [399].

Similar to SAXS, in situ Small‐Angle Neutron Scattering (SANS) is a versatile technique that provides valuable insights into the meso‐ and microstructures of materials (ranging from 1 to 300 nm), including particle sizes, shapes, agglomerates and nanostructured domains within battery cell components (Figure 19h) [408]. Unlike SAXS, which relies on x‐rays interacting with electron clouds, SANS uses a neutron source to probe the sample, offering unique advantages in detecting specific elements, particularly light elements such as hydrogen (H_2_) and Li, owing to their distinct nuclear interactions [409]. SANS operates through two primary scattering mechanisms: coherent and incoherent scattering. Coherent scattering results from wave interference (constructive or destructive), yielding information on the overall structural organization. In contrast, incoherent scattering arises from individual neutron‐nucleus interactions and provides insights into the dynamic behaviors of atoms and molecules. This dual capability allows SANS to simultaneously probe both the static structural features and dynamic processes within a material [386, 410]. The use of isotopic contrast in SANS further enhances its ability to resolve specific components within complex electrode architectures [411, 412]. For instance, Manke et al. utilized operando SANS to visualize Li^+^ transport in a solid‐state battery configuration (In/Li | Li_6_PS_5_Cl | S/C/Li_6_PS_5_Cl). By leveraging Li isotope contrast, they observed that Li became trapped in the cathode composite in the form of Li_2_S, which was not reconverted into elemental sulfur upon charging Li–S battery. The trapped Li was predominantly concentrated near the cathode [412]. In another study, Bradbury et al. investigated lithium‐thiophosphate‐based solid‐state Li–S batteries using operando SANS. Their 2D scattering data revealed that the reaction front propagated from the separator side toward the current collector during the initial discharge cycle. Post‐recharge 3D tomography showed residual Li near the current collector, indicating irreversible changes in the Li distribution over the charge‐discharge cycles [411]. By employing operando SANS, Lu et al. demonstrated that electrolyte accumulates in the edge areas, and electrolyte distribution in the cell can be observed even at 0 h. Further, Yan Lu distinguished unwetted regions and wetted regions. This study confirms that the poor wetting status and rather limited electrode‐electrolyte interfaces are caused by the heterogeneous distribution of electrolyte. With increasing rest time, it can be clearly seen that the unwetted areas and shapes in some local regions changed, indicating that the electrolyte redistributed. (Figure 19i) [400]. These SANS measurements are used to monitor the unwetted region and to design an efficient electrode. Together, these scattering techniques offer comprehensive insights into the nanoscale and atomic‐scale structural evolution of cathode materials during battery operation.

#### 6.1.3 In Situ Morphology Characterization Technique

The morphology of electrodes directly influences key electrochemical processes, such as electron conductivity, polysulfide conversion, and reversible capacity [413]. An optimized electrode structure significantly enhances MSBs' performance, while uneven or poorly designed morphologies cause critical failures during battery operation [414]. For instance, during electrochemical reactions, non‐uniform morphologies result in dendrite growth, cracking, and the formation of cathode electrolyte interphase (CEI) and SEI layers, which degrade battery performance [309]. In this context, in situ techniques are valuable for revealing dynamic changes during battery cycling, including surface roughening, dendrite formation, particle size evolution, and phase transformations. Understanding these structural transformations enables researchers to design electrodes that promote stable and uniform operation. Scholars have accelerated the practical development of battery technologies by leveraging insights into morphological evolution. This section discusses the recent advances in operando technique*s* used to visualize the electrode morphology.

**FIGURE 20:**
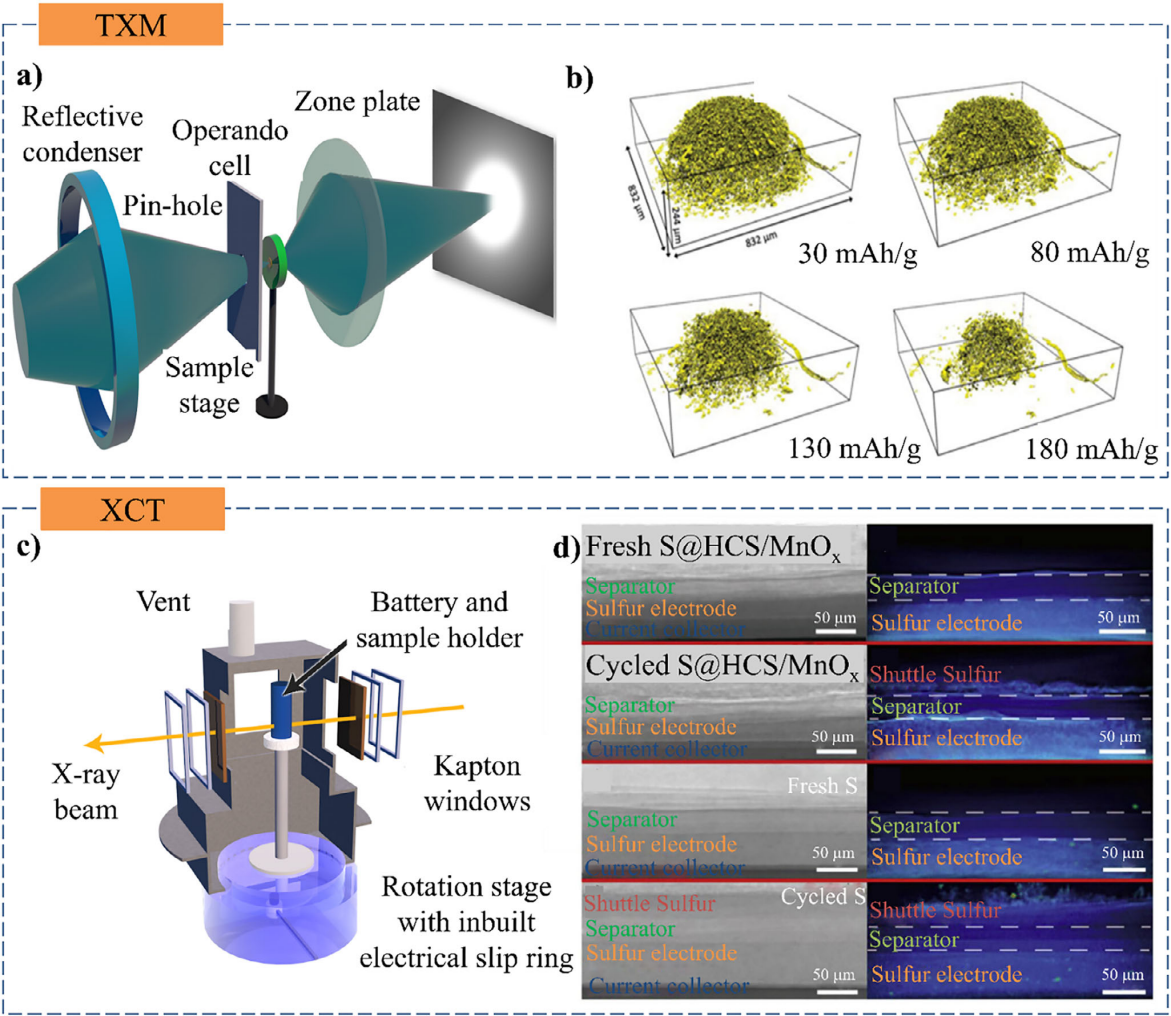
(a) Schematic representation of a TXM setup with zone plate optics for high‐resolution imaging of an operando cell, (b) volume renderings of cathode sulfur (reproduced with permission from ref. [419]. Copyright 2022, Wiley); (c) operando x‐ray tomography setup showing a rotating stage with an inbuilt electrical slip ring and x‐ray‐transparent Kapton windows for real‐time 3D visualization and (d) (Top 2) 2D radiographic and 3D volume rendering from tomography images of S@HCS/MnO_x_ electrode before and after one discharge/charge cycle at rate of 0.05 ​C, (Bottom 2) 2D radiographic and 3D volume rendering from tomography images of S electrode before and after one discharge/charge cycle of S@HCS/MnO_x_ electrode at rate of 0.05 C (reproduced with permission from ref. [425]. Copyright 2020, Elsevier).

Transmission x‐ray Microscopy (TXM) is a non‐destructive imaging technique that enables the high‐resolution (approximately 100 nm) visualization of 2D morphological changes in electrode materials (Figure 20a) [415, 416]. By measuring the absorption of transmitted x‐rays, TXM provides detailed insights into the internal structure, composition, and density of materials. Operando TXM captures volumetric changes in sulfur cathodes during the charge and discharge cycles. TXM also reveals critical morphological features, such as particle agglomeration, crack formation, void development, and phase transformations, which significantly impact battery performance. Importantly, TXM generates spatially resolved, element‐specific maps that highlight the distribution and localization of different phases within an electrode. These maps identify the active and inactive regions, assess electrode homogeneity, and diagnose the degradation mechanisms [417]. For example, Hwang et al. used TXM to demonstrate the role of polysulfide redox species in controlling Li deposition. Dense Li deposits were observed during the initial cycle. However, in the second plating cycle, a denser and more compact Li morphology formed near the copper substrate in Li_2_S||Cu cells, underscoring the critical influence of polysulfide redox reactions on achieving a high energy density in Anode‐Free Lithium‐Sulfur Batteries (AFLSBs) [418]. Jacob R. Bowen tested the cathode with different discharge currents and demonstrated the volume renderings of all sulfur particles in the cathode of the operando capillary cell. During the discharge cycle, all elemental sulfur has been converted into polysulfides and dissolved in the electrolyte. Further, Hwang et al. determined the volume of segmented S_8_ multiplied by the mass density (Figure 20b) [419]. Together, TXM is a powerful technique that demonstrates morphology changes of the electrode. Although TXM has a high resolution (10–100 nm) and high sensitivity, TXM is used to probe small sample sizes. To tackle these challenges, x‐ray Computed Tomography (XCT) has been employed.

XCT is an advanced absorption/phase‐contrast imaging technique (Figure 20c) [420, 421]. XCT captures three‐dimensional images by collecting transmitted x‐rays from multiple angles, enabling detailed visualization of internal structures. XCT is used to track electrode/particle morphology, including volume expansion, crack initiation/propagation, pore evolution, and inactive (electrochemically isolated) regions during cycling [386, 422, 423]. For example, Tonin et al. observed an increase in pit formation on Li anodes with rising current density, highlighting the link between operating conditions and surface degradation [424]. Similarly, Wang et al. utilized in situ XRT to examine volume expansion in Li–S cathode. Their study showed that S@hollow carbon sphere (HCS)/MnOx cathodes retained 70% of their structural volume and experienced only 16% volume expansion, whereas commercial sulfur cathodes displayed 40% expansion and retained 5% of their original sulfur volume (Figure 20d) [425]. In another study, Roué et al. employed XCT to evaluate the influence of various binders on electrode morphology during cycling and the discharge process. Among the various binders, Carboxymethyl Cellulose (CMC), Polyvinylidene Fluoride (PVdF), and poly (diallyldimethylammonium chloride) (PDDA), PDDA demonstrated superior initial capacity and cycling stability. Similarly, XCT analysis revealed that β‐S_8_ followed a deposition pattern in which the precipitated sulfur served as a nucleation site, forming elongated agglomerates. Notably, approximately 40% of the electrode volume was inactive at the end of the first discharge cycle [426]. Additionally, Barchasz et al. used XCT to probe structural modifications in crystalline S_8_ and lithium sulfide (Li_2_S) intermediates during the cycling process [427]. Bowen et al. employed operando XRT to monitor morphological changes, such as phase fraction evolution and particle size distribution in Li–S batteries. Their findings revealed the formation of a uniform and porous Li_2_S layer on the cathode with particle sizes below the tomographic resolution (<1 µm). As a result, scholars have observed that this layer hinders the polysulfide conversion and indicated that Li_2_S morphology is highly dependent on electrolyte formulation [428]. Through these operando techniques, scholars have identified the specific problem and designed specific materials to overcome the daunting challenges in MSBs.

### 6.2 Machine Learning for 2D Material Screening and Property Prediction

**FIGURE 21:**
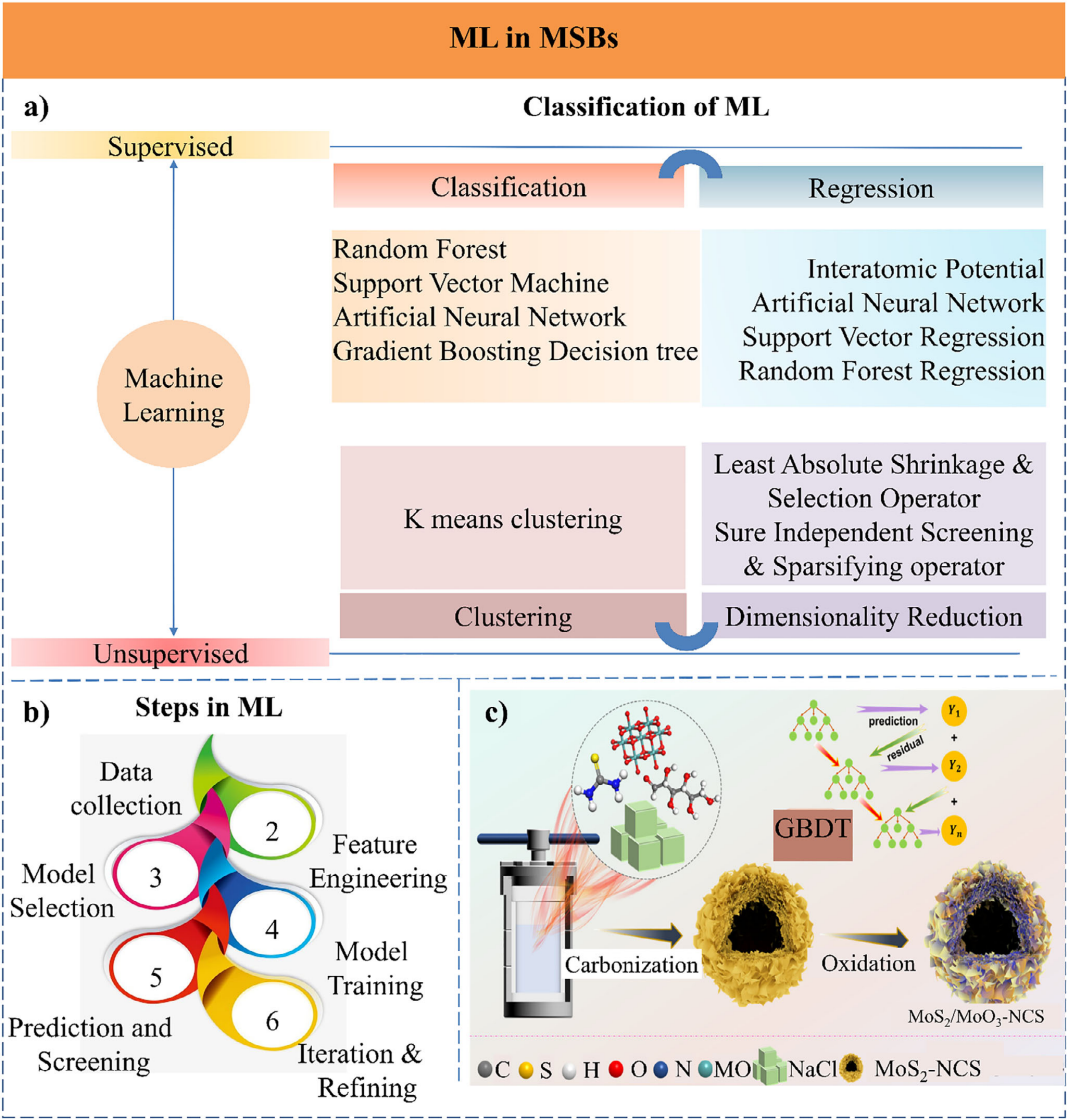
(a) ML algorithms applied to 2D materials research, (b) general steps involved in 2D materials research along with a few predictable properties, (c) graphical illustration of the preparation process of MoS_2_/MoO_3_‐NCS and the machine learning‐assisted data‐processing workflow (reproduced with permission from ref. [440]. Copyright 2025, Elsevier).

2D structures are distinguished by their exceptional electronic, physicochemical, and mechanical strengths. Exploration of their properties relies on experimental methods [158, 429, 430]. The experimental approach often requires numerous iterations, leading to a labour‐intensive trial‐and‐error process. Although experimental techniques are precise and provide a realistic representation of material characteristics, their inherent complexity is time‐consuming and hinders the rapid advancement of material discovery. In light of these challenges, with the advent of the Internet of Things (IoT), researchers have begun to utilize theoretical methods to predict the properties of 2D materials [431, 432]. ML is a subset of artificial intelligence (AI) that employs algorithms and statistical models that enable computers to perform tasks without explicit instructions. In materials science, ML has emerged as a transformative tool for accelerating the discovery, prediction, and optimization of material properties, particularly for MSB components. ML algorithms are broadly categorized into supervised models, which encompass classification and regression approaches, and unsupervised learning, which includes clustering and dimensionality‐reduction approaches. Once an appropriate algorithm is selected, essential data are generated using ML methods, such as K‐means, K‐medoids, linear discriminant analysis, decision trees, support vector machines, k‐nearest neighbors, and linear regression, which circumvent the trial‐and‐error experimentation (Figures 21a,b) [433, 434]. For instance, the bandgap of a 2D material can be predicted using graph neural networks (GNN), regression methods with the aNANt functional materials database and computational 2D materials database (C2DB) [435, 436, 437]. In GNNs, atoms in a material are represented as graph nodes, whereas chemical bonds or spatial relationships serve as edges. These models encode atomic features, such as elemental type, position, and bonding environment, to capture the complex topology and local electronic interactions of 2D lattices. Consequently, GNNs learn nonlinear relationships between the structure and properties, enabling bandgaps predictions, electrical conductivity, adsorption energies, and catalytic performance. Similarly, regression techniques identify complex nonlinear dependencies within large datasets of 2D materials, thereby improving the accuracy of structure‐property relationships [437, 438, 439].

In contrast, non‐equilibrium molecular dynamics (NEMD) simulations using interatomic potential (IP) methodology have been widely employed to assess the thermal and mechanical properties of 2D materials. For example, the thermal conductivity of TiS_2_/MoS_2_ heterojunctions and mechanical parameters, such as Young's modulus in XN_4_‐type 2D materials (X = Be, Mg, Pt) are predicted using IP approach [441, 442]. The IP‐based NEMD framework typically analyzes phonon scattering and heat flux correlation functions to evaluate the thermal conductivity (κ). It also deals with interfacial thermal resistance and anharmonic lattice vibrations, which are crucial for understanding heat transport in ultrathin layers [443]. For mechanical properties, NEMD simulations employ strain‐controlled deformation to compute Poisson's ratio, tensile strength, elastic modulus, and fracture toughness in 2D, such as InGeX_3_ (X = S, Se, Te) and the carbon Kagome lattice [424, 444]. The identification of 2D properties using the ML algorithm has saved time. In MSB systems, ML has played a pivotal role in predicting the binding energies of LiPSs on various 2D materials, thereby aiding in the identification of optimal host materials for effective shuttle‐effect suppression. Transfer learning models have been utilized to evaluate AB_2_‐type 2D materials, such as MoSe_2_ and WSe_2_, by encoding key atomic and electronic descriptors, including charge transfer characteristics, electronegativity, coordination number, and work function [439, 440, 441, 442, 443, 445, 446]. These features affect the catalytic behavior and polysulfide adsorption strength. For example, studies have shown that Li_2_S_4_ forms two Li–Se bonds with stronger binding than longer‐chain species, such as Li_2_S_6_ or Li_2_S_8_, which typically form only one [447]. Similarly, Jin and colleagues proposed a three‐step protocol called Informative Adsorption (InfoAd) to evaluate Li_2_S binding energy on 2D materials, combining high‐throughput DFT with ML to uncover structure‐property relationships. They built a database of 1255 stable 2D A_x_B_y_ monolayers (B from Group VIA/VIIA) extracted from C2DB and 2DMatPedia, calculating Li_2_S adsorption energy (𝐸_𝑎_) via GGA‐PBE DFT with Grimme D3 corrections. To classify materials by anchoring strength, they trained two ML models: XGBoost and C‐Infograph (Crystal Infograph). The XGBoost model showed that geometric features (bond lengths, bond angles, dihedral angles, space group number) outweigh elemental features in determining Li_2_S anchoring strength confirmed by polymorph pairs (CrO_2_, VS_2_, MnCl_2_, GeO_2_) where identical compositions but different crystal structures produced 𝐸𝑎​ differences up to 2.82 eV. This screening identified 44 nontoxic candidates, including MoO_2_ (𝐸_𝑎_​ = 3.44 eV), NiS (3.36 eV), TaS_2_ (3.28 eV), NiTe_2_ (3.24 eV) and TiTe_2_ (3.14 eV), all structurally stable (max atomic displacement <1.50 Å) and metallic (bandgap <0.03 eV). InfoAd thus accelerates discovery of high‐performance anchoring materials while revealing that geometry matters more than previously assumed [448]. Gong and colleagues built a high‐throughput framework combining DFT, graph convolutional networks (GCN), and physics‐informed learning to screen Li adsorption and its governing mechanism. Using a 180‐material subset of 2D metals from the Jain et al. database (VS_2_, MoS_2_, TiS_2_, NbSe_2_, TaS_2_, graphene, phosphorene and various dichalcogenides, carbides, nitrides, oxides, fluorides and phosphides), they trained a CGCNN‐based GCN potential to learn Li site energies across adsorption sites. This revealed a strong linear relationship between minimum Li adsorption energy (𝐸_𝑎𝑑𝑠_) and work function (Φ): 𝐸_𝑎𝑑𝑠_ =—Φ + 2.01 eV (𝑅_2_ = 0.77) establishing work function as the dominant descriptor. Applying this physics‐simplified model, they screened 3,499 2D metals and identified ultrahigh‐voltage materials Bi_2_F_2_ (𝐸_𝑎𝑑𝑠_ = ‐6.82 E), N_6_F_30_ (−7.48 eV), Cr_9_O_27_ (−7.10 eV) and Cr_4_P_8_O_32_ (−8.04 eV) that exceeded the prior 2D binding‐strength record of −5 eV. DFT studies confirmed these predictions, showing that the simplified physics‐based model could extrapolate beyond its training data, whereas the unsimplified model missed these candidates entirely [449].On the other hand, Zhou et al. demonstrated that MoGe_2_N_4_ and WGe_2_N_4_ anchor lithium polysulfides (LiPSs) and sulfur species, with adsorption energies of 0.72–2.67 eV, offering improved kinetics and charge‐discharge efficiency for MSB cathodes [445]. In a broader screen, an ML algorithm evaluated 1320 AB_2_‐type 2D compounds from 2DMatPedia and identified 14 promising candidates PdN_2_, TaS_2_, PtN_2_, TaSe_2_, AgCl_2_, NbSe_2_, TaTe_2_, AgF_2_, NiN_2_, AuS_2_, TmI_2_, NbTe_2_, NiBi_2_ and AuBr_2_ each showing strong Li_2_S_6_ adsorption (>1.0 eV) and excellent conductivity. Notably, this ML‐driven approach cut screening time by roughly eight years compared to conventional methods [450]. Kumar and colleagues developed an integrated ML‐DFT strategy to study N_6_‐coordinated dual‐atom catalysts (DACs) on nitrogen‐doped graphene for the sulfur reduction reaction (SRR). Their workflow, PACE (Precise and Accelerated Configuration Evaluation), pairs machine‐learned interatomic potentials (MACE‐mp‐0) with automated configuration screening to rapidly locate low‐energy adsorbate geometries. For an Fe/Cu‐doped N_6_‐graphene system with Li_2_S, PACE screened 512 unique adsorption configurations across 46 400 generated structures, leveraging GPU‐parallelized MLIP calculations before refining the top candidate with DFT. Binding energies of Li_2_S_n_ (n = 1–8) across various DACs were grouped as weak (−0.7 to −1.5 eV), moderate (−1.5 to −2.5 eV), or strong (more exothermic than −2.5 eV). The (N_3_)Fe‐Ni(N_3_) and (N_3_)Fe‐Pt(N_3_) DACs showed the best SRR performance, with adsorption energies of _‐_1.0 to _‐_2.3 eV, low free‐energy changes (ΔG ≤ 0.5 eV) for Li_2_S_2_→Li_2_S conversion and modest Li_2_S decomposition barriers (≤1.0 eV; specifically 1.0 eV and 1.08 eV, with conversion ΔG of 0.36 and 0.39 eV, respectively). Other DACs (N_3_)Fe‐Cu(N_3_), (N_3_)Fe‐Fe(N_3_), (N_3_)Fe‐Co(N_3_) showed higher decomposition barriers (1.07–1.35 eV). Several DACs, including (N_3_)Co‐Co(N_3_), (N_3_)Fe‐Mn(N_3_), (N_3_)Fe‐V(N_3_), (N_3_)Fe‐Ni(N_3_) and (N_3_)Fe‐Pt(N_3_), also showed Li‐diffusion barriers ≤1.0 eV, indicating strong catalytic activity during charging. To predict ΔG for unexplored DACs, the authors built a Gradient Boosting Regression model using interpretable descriptors (average ICOHP between TM and S, average TM‐S and Li–S bond lengths, electronegativity difference, sulfur count, and inter‐metal electron affinity), achieving MAE = 0.26 eV and test 𝑅_2_ = 0.85. Electronic structure analysis attributed the superior catalysis to frontier‐orbital coupling between adjacent metal centers acting as a “charge‐transfer highway” with optimal metal‐metal bonding and favorable d‐band positioning tuning adsorbate binding. (N_3_)Fe‐Ni(N_3_) showed a low decomposition barrier (1.0 eV), a highly exergonic overall reaction (ΔG_overall_ = −1.93 eV), and the lowest ΔG(Li_2_S_4_→Li_2_S) of 0.787 eV. This PACE‐ML‐DFT framework offers a generalizable strategy for accelerated DAC catalyst design in next‐generation Li–S batteries [451]. Wang and colleagues proposed a p‐p‐s orbital electronic coupling descriptor to guide selection of anion dopants for Li–S catalysts, combining machine learning, DFT, and experimental validation. They synthesized five anion‐doped (B, N, P, S, Te) WSe_2_/MXene catalysts via one‐step hydrothermal synthesis, with B‐WSe_2_ forming distinctive petal‐like nanostructures (∼100 nm) on the MXene surface. DFT‐calculated Li_2_S_6_ adsorption energies ranked: P‐WSe_2_ (−1.342 eV) > N‐WSe_2_ (−1.331 eV) > B‐WSe_2_ (−1.147 eV) > S‐WSe_2_ (−1.017 eV) > Te‐WSe_2_ (−1.001 eV) > pristine WSe_2_ (−0.923 eV) showing P‐WSe_2_ binds strongest while B‐WSe_2_ offers moderate adsorption. For the rate‐limiting Li_2_S_2_→Li_2_S step, free‐energy barriers followed: B‐WSe_2_ (0.626 eV) <N‐WSe_2_ (0.674 eV) <P‐WSe_2_ (0.699 eV) <S‐WSe_2_ (0.721 eV) <Te‐WSe_2_ (0.762 eV) <pristine WSe_2_ (0.772 eV), with B‐WSe_2_ showing the lowest barrier and largest equilibrium constant (7.71 × 10^−22^), indicating the fastest, most complete sulfur reduction. CI‐NEB calculations of Li_2_S decomposition barriers followed the same trend: B‐WSe_2_ (0.923 eV) <N‐WSe_2_ (1.058 eV) <P‐WSe_2_ (1.339 eV) <S‐WSe_2_ (1.380 eV) <Te‐WSe_2_ (1.381 eV) <pristine WSe_2_ (1.420 eV), confirming B‐WSe_2_’s superior ability to drive Li_2_S phase transformation and boost sulfur utilization. Arrhenius‐derived activation energies for Li_2_S oxidation further validated B‐WSe_2_ as most effective (3.99 kJ/mol vs. 21.62 kJ/mol for pristine WSe_2_) [452].

Beyond electrode materials, association rule mining, a data mining technique, has been used to identify optimal electrolyte formulations. This method analyzes co‐occurrence patterns between variables, such as solvent type, salt concentration, and additives, and correlates with battery performance indicators, such as cycle life, capacity retention, and suppression of polysulfide shuttling. Statistical metrics, such as confidence, lift, and conviction, were used to validate these correlations. A recent meta‐analysis of 207 experimental data points from 42 studies revealed that ionic liquids based on 1‐ethyl‐3‐methylimidazolium bis(trifluoromethanesulfonyl)imide (EMI‐TFSI) and n‐methyl‐n‐propylpyrrolidinium (P13) consistently offer higher specific capacities. Additionally, Li(tetraglyme) bis(trifluoromethanesulfonyl)imide (Li[G3]TFSI) emerged as a promising candidate for system‐level energy density due to its stability and compatibility with LiPSs [453]. Similarly, Su et al. utilized an ML technique to optimize a 2D heterostructure composite (MoS_2_/MoO_3_@C) (Figure 21c). The ML framework employed the GBDT method to find the material composition, heterointerface configuration, and synthesis parameters. ML technique was used to optimize a 2D heterostructure composite (MoS_2_/MoO_3_@C). The ML framework employed the GBDT method to find the material composition, heterointerface configuration, and synthesis parameters. By optimizing these parameters, Qingmei Su synthesized MoS_2_/MoO_3_@C to maximize polysulfide adsorption and conversion kinetics [440]. Overall, ML analysis highlights a multi‐faceted approach to material discovery. For electrolyte optimization, researchers have employed association rule mining, Random Forest, and Transformer models. These model helps to identify optimal combinations of solvents, salts and additives. In host material screening, a broader suite of ML algorithms including XGBoost, C‐Infograph (a graph neural network), graph convolutional networks (GCN), Random Forest, transfer learning, and various GNN architectures such as CGCNN, ALIGNN, and Geo‐CGNN has been utilized. For electrocatalyst design, GBR, PACE, Random Forest, and ML‐derived binary descriptors have been utilized. By using these ML models, scholars have circumvented the time.

## 7 Conclusions and Future Directions

2D materials represent a versatile class of functional materials that address the multifaceted challenges in MSBs, including sulfur insulation, polysulfide shuttling, dendrite growth, interfacial instability, and poor ion transport. Their advantages stem not merely from dimensionality, but rather from a synergistic combination of strong in‐plane bonding, weak interlayer interactions, tunable surface chemistry, and mechanical anisotropy. In the cathode, conductive 2D matrices, such as GDY and phosphorene, mitigate the inherent insulating nature of sulfur by providing electron pathways. Their high surface areas and tunable pore sizes accommodate large amounts of sulfur motifs and expose more active sites to accelerate the redox reactions. The functionalized surface groups in the 2D matrix (siloxene, MXene) chemically anchor polysulfides via covalent bonds or Lewis acid‐base interactions, significantly curtailing the shuttle effect. The 2D anodic host exhibited a uniform lithiophilic/sodiophilic layer that enhanced Li/Na nucleation. 2D materials with large surface areas facilitate the diffusion of metallic ions, thereby preventing dendrite growth. Furthermore, the 2D material enhanced the adhesion energy between the Li/Na metal. 2D materials with strong mechanical properties prevent electrode cracking; for instance, a high Young's modulus maintains structural integrity under stress, a high tensile strength allows the material to withstand deformation, and a negative Poisson's ratio (in materials like borophene) can improve structural stability by contracting laterally under tension, which prevents crack propagation. The interlayer spacing of 2D materials, along with surface charge, plays a significant role in regulating polysulfide migration in the separator. Furthermore, the high thermal conductivity of 2D materials prevents thermal runaway damage. 2D fillers alters the dielectric constant in the electrolyte, thereby improving the ionic conductivity and improving the mechanical properties. However, to accelerate the practical feasibility of MSBs, 2D materials must be scalable, cost‐effective, and environmentally sustainable. Although many 2D materials show excellent lab‐scale performance, they impede mass production and material consistency. Standardizing low‐cost synthesis methods is the key to the industrialization of these technologies.

### 7.1 Scalability, Cost, Environmental Impact

2D materials such as graphene, GDY, borophene, antimonene, phosphorene, MXene, germanene, siloxene, and MBene exhibit exceptional electrochemical and structural properties. However, a quantitative analysis of their synthesis routes reveals a widening industrialization gap between graphene and next‐generation 2D materials. To date, graphene was one of the most synthesized 2D materials. In general, graphene was synthesized via. Liquid‐phase exfoliation (LPE) of graphite in NMP achieves a monolayer yield of 1 wt%, improvable to 7–12 wt% with further processing, while shear exfoliation achieves ∼30% few‐layer yield from graphite in 45 min, with over 61% of flakes comprising fewer than five layers. Commercial graphene oxide costs US$100‐500/kg and graphene is the only 2D material with demonstrated ton‐scale industrial throughput; a solvent‐free dry exfoliation process introduced by NanoXplore in 2023 further reduces capital expenditure relative to liquid‐based methods. MXene (Ti_3_C_2_T_x_) is the second most scalable 2D material. Conventional HF‐based etching followed by delamination is the dominant production route, with one detailed cost analysis predicting $20.33/g at laboratory scale, a cost expected to fall substantially with reactor scale‐up, since the wet etching reaction occurs volumetrically rather than on a substrate surface, enabling direct scale‐up with reactor volume. A hydrothermal etching followed by water‐only delamination route now achieves 98% multilayer Ti_3_C_2_T_x_ yield with an electrical conductivity of 15,230 S cm^−1^, while a low‐temperature molten salt (LTMS) etching method further reduces processing time to just 5 min for diverse MXene compositions including V_4_C_3_T_x_, Nb_4_C_3_T_x_ and Mo_2_CT_x_. In sharp contrast, GDY remains severely constrained by synthesis complexity and time. The standard synthesis requires a glove‐box environment, a copper catalyst, and multi‐step deprotection procedures, and strong interlayer π‐π stacking limits yield and lateral size; solubility up to 4.3 mg ml^−1^ has been achieved by framework charge‐induced intercalation, but gram‐scale, monolayer crystalline GDY remains confined to the laboratory with no commercial large‐scale synthesis. It is also worth noting that 10 mg of GDY is about $594. Borophene presents an even more severe barrier. MBE, CVD, and UHV epitaxial growth result in low material yield and are confined to substrate‐supported films; scalable synthesis of elemental 2D materials beyond graphene still remains elusive, and persistent challenges including air instability, phase inconsistency, and device integration continue to hinder practical deployment. Phosphorene (exfoliated black phosphorus) faces the dual limitation of high exfoliation energy (_‐_151 meV per atom, substantially higher than graphite) and rapid ambient degradation during LPE; long sonication times degrade nanosheet quality, mechanical exfoliation is limited to laboratory scale, and CVD growth is technically challenging and cost‐intensive. Silicene and germanene face a fundamental structural barrier: unlike graphene, black phosphorus, silicene and germanene do not exist as bulk layered structures. It can be synthesized via MBE on metal substrates (Ag(111), Cu(111)) and demonstrated synthesis route via complex, cost‐intensive and providing only micrometer‐scale domains with no gram‐scale output. Siloxene occupies a slightly more accessible position: it can be synthesized by topochemical deintercalation of CaSi_2_ with concentrated HCl at low temperatures, producing Kautsky‐type Si_6_H_3_(OH)_3_ sheets, though the current literature still lacks definitive evidence to comprehensively characterize the coordination chemistry of these silicon nanosheets, and the method suffers from slow kinetics, safety concerns from concentrated acid, and incomplete structural control. MBene represents the youngest and least experimentally validated member of the 2D material family. Derived from MAB phases by selective etching analogous to MXene synthesis, MBenes suffer from stronger M‐B bonds than the M‐Al bonds in MAX phases, leading to frequent problems of incomplete etching, oxidation of MAB phases, and phase transformation during synthesis; there are only a few experimental reports on MBenes synthesis using top‐down or bottom‐up methods, current research is largely theoretical, and no scalable route, gram‐scale yield, or commercial cost has been reported. Taken together, these data confirm a three‐tier industrialization hierarchy among 2D materials: graphene alone operates at industrial scale with LPE yields of 7–30 wt%, costs of $100‐500 kg^−1^ and sub‐hour cycle times; MXene occupies a pilot‐scale intermediate position at $20.33 g^−1^ with 98% etching yield and 5‐minute processing time; Similarly, 10 mg of GDY required $550 and 10 mg of phosphorene and Borophene required $750 and 3550 and all remaining next‐generation 2D materials silicene, germanene, siloxene, MBene, remain at the laboratory scale with yields below 1 wt% and synthesis times ranging from hours to days under stringent inert, vacuum, or hazardous‐acid conditions. To close this gap, urgent adoption of electrochemical exfoliation with mild aqueous electrolytes (Na_2_SO_4_, H_2_SO_4_), solvent‐free mechanochemical synthesis, low‐temperature solvothermal routes and one‐pot in situ functionalization is needed across all material classes, alongside closed‐loop solvent recovery systems and rigorous lifecycle assessment (LCA) to validate environmental sustainability from raw material extraction through end‐of‐life disposal, ensuring that the commercialization of 2D‐material‐enabled metal‐sulfur batteries aligns with global energy and sustainability goals.

### 7.2 ML Integration and Industry Adoption

The practical feasibility of RT‐MSBs holds immense promise owing to their high theoretical energy densities and the natural abundance of active materials. However, several persistent challenges limit their transition from laboratory research to large‐scale industrial application. One critical limitation is that materials developed under ideal conditions show inconsistent performance under real‐world battery operating parameters owing to wide environmental factors, including temperature fluctuations and humidity. Furthermore, scaling up the synthesis often results in inconsistent material properties. Furthermore, the disparity between laboratory‐based performance poses a major barrier to industrial adoption. Addressing these multifaceted problems requires material design with respect to real‐world operational conditions and consistent properties in scalable quantities. These can be addressed by integrating ML into battery research. ML techniques accelerate material suitability assessment and predict battery behavior by optimizing the cell components and parameters under real‐world operational conditions. For instance, supervised learning algorithms predict the ionic conductivity or mechanical robustness of electrolytes based on their chemical compositions and structural features.

**FIGURE 22:**
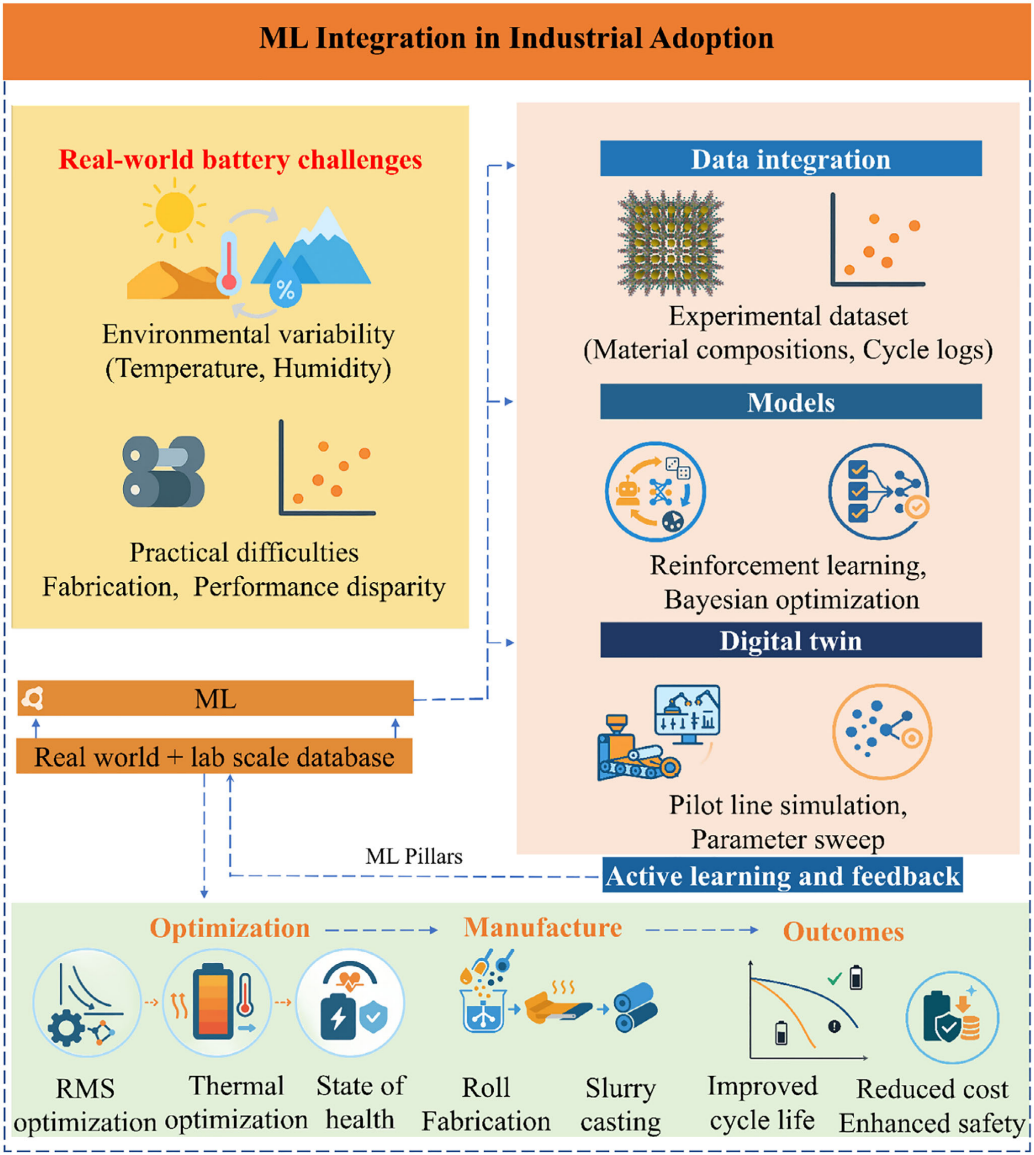
Schematic overview of practical feasibility challenges, such as material reproducibility, performance inconsistency, and scalability; integration of ML techniques for material development from lab scale to industry via supervised/unsupervised learning; industrial optimization through roll‐to‐roll manufacturing, slurry processing, and low‐cost precursors; and real‐world performance enhancement via LSTM‐based models and reinforcement learning.

Unsupervised clustering identifies suitable electrode materials that are compatible with electrolytes and exhibit synergistic behaviors, such as suppression of the shuttle effect or enhancement of SEI/CEI stability. From an industrial standpoint, ML extends beyond material screening to real‐time environmental prediction and operational optimization. Models such as Long Short‐Term Memory (LSTM) networks and Recurrent Neural Networks (RNNs) have been employed to predict real‐time temperature, atmospheric humidity, and their dynamic impact on battery performance and degradation. Similarly, Reinforcement learning and Bayesian optimization have been utilized to optimize the synthesized material in real time, offering a path toward battery management systems. The convergence of ML and battery research aligns with the growing demand for high‐throughput development and cost reduction. ML also supports digital twin models for pilot line validation, enabling manufacturers to simulate and optimize full‐cell performance before physical prototypes are produced. ML also ensures that the designed catalyst not only delivers high performance but is also compatible with roll‐to‐roll manufacturing, slurry casting, and industry‐standard fabrication. In conclusion, by coupling advanced ML models with experimental insights, researchers can bridge the gap between scientific innovation and practical RT‐MSB development. Figure 22 highlights how ML accelerates industrial adoption of batteries by linking real‐world challenges. This includes environmental variability (temperature/humidity) along with manufacturing‐driven performance disparities. By analyzing lab‐scale databases and real world problem, ML data‐driven models (e.g., reinforcement learning and Bayesian optimization and leverages digital twins for pilot‐line simulation and parameter sweeps) was chosen to enable active learning and continuous feedback from manufacturing back to optimization; together, these ML models target practical objectives including RMS/quality optimization, thermal management, SOH prediction and process improvements (roll fabrication, slurry casting), ultimately delivering longer cycle life, enhanced safety and reduced cost.

### 7.3 Emerging 2D Materials

**FIGURE 23:**
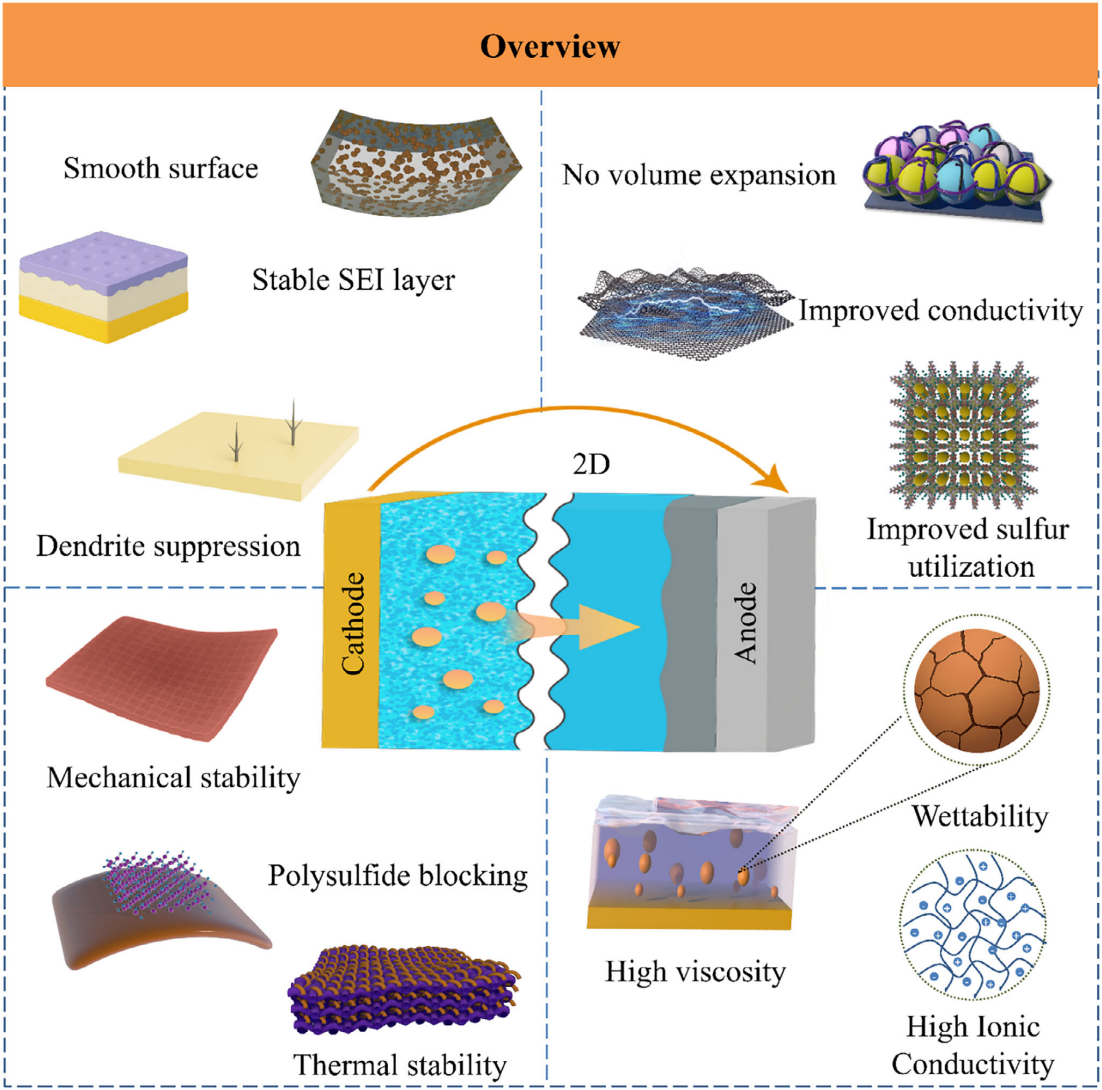
Multifunctional roles of 2D materials in MSBs. 2D materials form a stable SEI layer and suppress dendrite growth on the anode. Whereas on the sulfur cathode side, 2D hosts immobilize polysulfides, improve electronic pathways, and prevent volume expansion. When integrated into separators, 2D‐material modification can enhance mechanical/thermal robustness and improve electrolyte wettability, contributing to safer and more stable cell operation.

Figure 23 displays how 2D materials address bottlenecks of MSBs. Initially, on the anode side, 2D coatings form a more stable SEI layer smooth surface. Whereas on the sulfur cathode side, 2D materials with high surface area and polar/active sites can immobilize polysulfides (mitigating shuttling), improve electronic/ionic pathways, and prevent volume expansion. Similarly, 2D materials improve the mechanical and thermal stability of the separator and enhance the wettability. 2D fillers improves the ionic conductivity in the electrolyte. Building on this picture, to improve the energy density and longevity of MSBs, next‐generation 2D materials should be considered. At the cathode, self‐healing binders, such as supramolecular‐based binders, hydrogen‐bonded poly(urea‐urethane), or 2D conductive binder poly(3,4‐ethylenedioxythiophene)(PEDOT): polystyrene sulfonate (PSS), and so forth, should be considered. These binders utilize noncovalent interactions, including hydrogen bonds and π–π stacking, to autonomously repair mechanical cracks caused by the significant volumetric expansion of sulfur during the cycling process. The sulfonate (‐SO^3−^) groups in PEDOT: PSS further mitigate polysulfide shuttling via Lewis acid‐base interactions, while conjugated thiophene backbones maintain electronic conductivity under strain. Concurrently, 2D catalytic cathodes functionalized with sulfur‐binding moieties chemically anchor polysulfides through covalent M‐S bonds or electrostatic repulsion, enhancing sulfur utilization and redox kinetics. Bio‐inspired 2D coatings, such as polydopamine‐functionalized materials, utilize abundant catechol/quinone and amine functional groups, enabling strong polar interactions, hydrogen bonding, and chemical reactivity with polysulfides when used in separators. Additionally, the tunable interlayer spacing of these 2D materials functions as a molecular sieve, allowing Li^+^ or Na^+^ ions to diffuse freely while blocking larger polysulfide (S_x_^2−^) species. Similarly, defective 2D insulating materials can be fabricated as interlayers. These defects create chemically active sites that selectively adsorb polysulfides through weak interactions. This controlled adsorption mitigates the polysulfide shuttle effect by immobilizing soluble intermediates and maintaining rapid Li/Na‐ion transport across the interlayer. At the anode, dendrite suppression is achieved through 2D polymer hosts. This 2D polymer host, such as disulfide‐linked composites, undergoes disproportionation‐recombination reactions to repair cracks caused by plating/stripping according to the Griffith crack theory. To fortify the artificial SEI, 2D shape‐memory alloys should be considered. These materials utilize martensitic‐austenitic phase transitions to recover their structural integrity after deformation and provide resistance to electrolyte‐induced degradation. In the case of electrolyte emerging 2D fillers, such as 2D‐MOF/COF composite can be utilized to improve the ionic conductivity and transfer number. In conclusion, emerging 2D materials should be considered to improve the energy capacity and longevity of the MSBs.

## Author Contributions

**Yao‐Jie Lei**: investigation, writing – review and editing, validation, visualization. **Guoxiu Wang**: conceptualization, investigation, writing – review and editing, visualization, validation, supervision. **Hua Kun Liu**: investigation, writing – review and editing, software, formal analysis, supervision, resources, visualization, validation. **Xuebing Zhu**: investigation, writing – review and editing, validation, visualization. **Naveen Kumar T. R**: conceptualization, investigation, writing – original draft, formal analysis, validation, visualization. **Yun‐Xiao Wang**: conceptualization, investigation, funding acquisition, writing – review and editing, visualization, validation, methodology, software, formal analysis, project administration, resources, supervision, data curation. **Lei Zhang**: investigation, validation, writing – review and editing, visualization. **Weihong Lai**: investigation, conceptualization, writing – review and editing, visualization, validation, supervision.

## Conflicts of Interest

The authors declare no conflicts of interest.

## Data Availability

No primary research results, software or code have been included and no new data were generated or analysed as part of this review.
